# RNA-Seq Uncovers Association of Endocrine-Disrupting Chemicals with Hub Genes and Transcription Factors in Aggressive Prostate Cancer

**DOI:** 10.3390/ijms26125463

**Published:** 2025-06-06

**Authors:** Diaaidden Alwadi, Quentin Felty, Mayur Doke, Deodutta Roy, Changwon Yoo, Alok Deoraj

**Affiliations:** 1 Department of Environmental Health Sciences, Florida International University, Miami, FL 33199, USA; dalwa002@fiu.edu (D.A.); feltyq@fiu.edu (Q.F.); droy@fiu.edu (D.R.); 2 Diabetes Research Institute, University of Miami, Miami, FL 33136, USA; mad1188@miami.edu; 3 Biostatistics Department, Florida International University, Miami, FL 33199, USA; cyoo@fiu.edu

**Keywords:** androgen receptor, differentially expressed genes, endocrine-disrupting chemicals, environmental health risk assessment, environmental carcinogenesis, prostate cancer, RNA-seq, transcription factors

## Abstract

This study analyzes publicly available RNA-seq data to comprehensively include the complex heterogeneity of prostate cancer (PCa) etiology. It combines prostate and prostate cancer (PCa) cell lines, representing primary PCa cells, Gleason scores, ages, and PCa of different racial origins. Additionally, some cell lines were exposed to endocrine-disrupting chemicals (EDCs). The research aims to identify hub genes and transcription factors (TFs) of the prostate carcinogenesis pathway as molecular targets for clinical investigations to elucidate EDC-induced aggressiveness and to develop potential biomarkers for their exposure risk assessments. PCa cells rely on androgen receptor (AR)-mediated signaling to survive, develop, and function. Fifteen various RNA-seq datasets were normalized for distribution, and the significance (*p*-value < 0.05) threshold of differentially expressed genes (DEGs) was set based on |log2FC| ≥ 2 change. Through integrated bioinformatics, we applied cBioPortal, UCSC-Xena, TIMER2.0, and TRRUST platforms, among others, to associate hub genes and their TFs based on their biologically meaningful roles in aggressive prostate carcinogenesis. Among all RNA-Seq datasets, we found 75 overlapping DEGs, with BUB1B (32%) and CCNB1 (29%) genes exhibiting the highest degree of mutation, amplification, and deletion. EDC-associated CCNB1, BUB1B, and CCNA2 in PCa cells exposed to EDCs were consistently shown to be associated with high Gleason scores (≥4 + 3) and in the >60 age group of patients. Selected TFs (E2F4, MYC, and YBX1) were also significantly associated with DEGs (NCAPG, MKI67, CCNA2, CCNB1, CDK1, CCNB2, AURKA, UBE2C, BUB1B) and influenced the overall survival (*p*-value < 0.05) of PCa cases. This is one of the first comprehensive studies combining 15 publicly available RNA-seq datasets to demonstrate the association of EDC-associated hub genes and their TFs aligning with the aggressive carcinogenic pathways in the higher age group (>60 years) of patients. The findings highlight the potential of these hub genes as candidates for further studies to develop molecular biomarkers for assessing the EDC-related PCa risk, diagnosing PCa aggressiveness, and identifying therapeutic targets.

## 1. Introduction

Prostate cancer (PCa) is the second leading cause of cancer-related death in men and the most commonly diagnosed cancer in the United States. Consequently, the incidence of PCa cases diagnosed has risen from 3.9% to 8.2% over the last decade [1]. In 2022, the number of estimated new cases in the USA was 268,490 (rated first at 27% among the top 10 cancers), and assessed deaths were numbered at 34,500 (rated second at 11% among the top 10 cancers) for PCa in men [1,2,3]. The normal prostate gland consists of basal and luminal cells enclosed by fibromuscular stroma. During the development of PCa, the ratio of luminal to basal cell percentages is significantly changed, with the luminal cells including more than 99% of the cancer [4]. The growth of PCa is facilitated by the hormones interacting with the androgen receptor (AR), whose dysregulated gene expressions are linked to the development of PCa with a Gleason score ≥ (4 ± 3) [5]. AR consists of four domains: (1) N-terminal, (2) DNA-binding, (3) hinge region, and (4) ligand-binding that binds with androgens, such as Testosterone and Dihydrotestosterone (DHT) [6]. Normal physiological functions, prostate carcinogenesis, and androgen deprivation therapy (ADT) affect AR-mediated signaling, which also plays a significant role in treating PCa [7]. AR signaling (AR activity) can stimulate or suppress the growth of PCa based on the level of androgen, which has resulted in the development of the ADT technique [7,8]. Present PCa examinations, including computed tomography (CT), digital rectal examination, magnetic resonance imaging (MRI), and ultrasound detection, overlook middle- and high-risk lesions in tissue, and these diagnostic methods exhibit limitations in the biochemical detection of PCa recurrence [9,10]. Detection of PSA is also prone to false positives resulting from benign prostatic hyperplasia, and its levels are frequently altered by different triggers (inflammation or sexual activity), which result in overdiagnosis [9,11,12,13]. Distinct protein expressions and genes between malignant and normal prostate tissues have been recorded, which provided resources for diagnostic, prognostic, and biomarker identification [10,14,15,16,17,18,19,20,21,22,23,24,25,26]. Epigenetic modifications, gene mutations, and microRNA expression in carcinogenesis have also been researched for biomarker findings [14,15,16,17,18,19,20,21,22,23,24,25,26]. Many researchers have discovered gene expression profiles that can help elucidate the PCa prognosis by analyzing single or multiple microarray datasets. For example, BUB1, CDK1, and EZH2 [14], EPCAM, TWIST1, CD38 and VEGFA [15], PTEN (Phosphatase and Tensin Homolog) [16], ISG15 and CST2 [17], BUB1, KIF2C, CDC20, and PBK [18], LMNB1, TK1, RACGAP1 and ZWINT [19], CKS2, TK1, and RRM2 [20], FGFR1, FGF13 and CCND1 [21], BZRAP1-AS1 and KRT8 [22], MP2, PPARG and PRKAR2B [23], ABCC4 and SLPI [24], CENPA, KIF20A and CDCA8 [25], and MCM4, CENPI, and KNTC1 [26] genes have been suggested to be potential biomarkers. Recently, there has been a significant increase in public RNA sequencing datasets (RNA-seq), which can be analyzed for gene set enrichment (GSE).

However, most of the publicly available microarray and RNA-seq-based datasets comprise clinically valuable samples or experimental types, often lacking information on the environmental exposure effects of single or multiple factors. The availability of high-throughput gene microarray data and RNA-seq data from various cancer cell types, treatments, and cancer tissue specimens, along with comparative toxicogenomic datasets, provides opportunity to simultaneously fill knowledge gaps in the molecular pathology of cancers due to environmental exposures at both the transcriptional and translational levels [27,28]. The prognosis of PCa can be significantly influenced by EDCs (or xenobiotics) in the environment. EDCs have been linked with poor PCa prognosis [29,30,31,32,33]. EDCs may act as agonists or antagonists for AR; they can directly bind to AR, alter gene expression, interfere with steroidogenesis, or induce epigenetic changes. Our earlier studies demonstrated the association of environmental phenols and parabens with patient-reported PCa diagnoses [34], with 22 environmental chemicals, including 17 EDCs, potentially impacting PCa carcinogenesis and its aggressiveness [35]. EDCs have also been shown to significantly influence DNA mutations, chromosomal abnormalities, and inflammation [5,36].

This study aims to capture the range of heterogeneity present within prostate cancer cells. It includes a comprehensive analysis of the GSE of 15 RNA-seq datasets, which combine prostate and PCa cell lines. The pool represents RNA seq data from primary PCa cells, PCa cells with high Gleason scores that suggest the level of PCa aggressiveness, and PCa cells of different racial origins. These cell lines were either treated with androgen modulators, such as R1881 (an androgen agonist), used to investigate the impact of the TFs, or exposed to xenobiotic EDCs. The pooled GSE enabled the evaluation of molecular signatures associated with EDC exposure and the prognosis of aggressive PCa. By examining comprehensive RNA-seq data and GSE, the objective of the study is to understand the potential roles of EDC-associated hub genes and their TFs in the aggressiveness of prostate cancer (PCa). These genes and their TFs may serve as biomarkers for assessing environmental health risks associated with EDC exposures. The findings may also pave the way for further research to validate their potential as biomarkers to improve the prognosis of aggressive prostate cancer cases.

## 2. Results

### 2.1. Discovery of Differentially Expressed Genes (DEGs) in RNA-Seq for PCa

Fifteen expression data series (GSE) RNA-seq datasets (GSE70466, GSE151290, GSE128749, GSE120660, GSE13587, GSE136272, GSE218556, GSE64529, GSE5590, GSE128339, GSE109021, GSE200879, GSE103512, GSE104131, GSE200167) included 41 platforms for PCa that were normalized using DESeq2, and the post-normalized value is demonstrated by all by 41 box plots of all datasets as shown in Figure S1. Our data contained 364 total samples of cell lines: 272 tumors and 92 normal prostate cell samples. The RNA-seq datasets were divided into four groups: cell lines treated with R1881 treatment, cell lines exposed to EDCs (Bisphenol A, DHT, Methyl testosterone, Estrogen, Testosterone propionate, DDE, and triclosan), cell lines with various Gleason scores and cells with MYC (TF) functional analysis, and cell lines with different ages and races. A total number of 10,241 DEGs were obtained from RNA-seq datasets assessed in the analysis based on selected criteria of the cutoff standards of adjusted *p*-value < 0.05 and [log2FC] > +2, [log2FC] < −2 as illustrated in volcano plots from OriginPro 9.1 shown in Figure S2.

**Figure 1:**
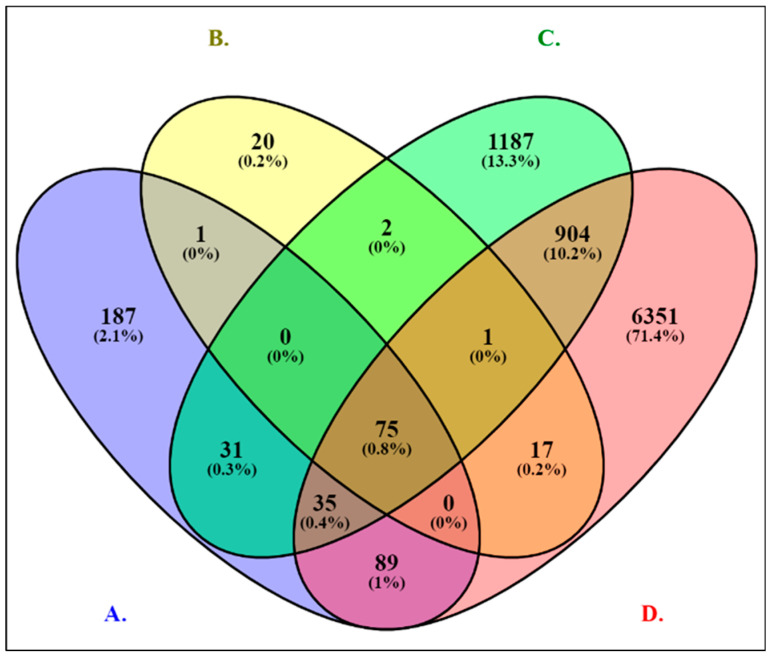
Venn diagram analysis of 4 groups of DEGs in PCa cell lines across RNA-seq datasets represented in blue (**A**), yellow (**B**), green (**C**), and red (**D**) panels. Panel (**A**) displays 418 DEGs common to cell lines treated with R1881 treatment (AR agonist). Panel (**B**) shows 116 DEGs shared among cell lines with EDC exposures, which also included AR modulators. Panel (**C**) illustrates 2235 DEGs shared between Gleason scores and cells overexpressing MYC (TF) for functional analysis. Panel (**D**) represents 7472 DEGs overlapping between age and race. A total of 75 (0.8%) overlapping genes are among the RNA-seq datasets.

In the next step, we generated a Venn diagram of four groups of overlapping genes from RNA-seq datasets of PCa cell lines with (1) R1881 treatments (418 genes), (2) exposure datasets (116 genes), (3) Gleason scores + MYC (TF) (2235 genes), and (4) age/race (7472 genes). This result is shown in Figure 1. To obtain the most significant overlapping DEGs from all RNA-seq datasets, we generated a Venn diagram showing 75 (0.8%) genes, including 37 upregulated and 38 downregulated genes. These identified 75 genes were considered for further analysis.

### 2.2. RNA-Seq DEG Enrichment Analysis: GO, KEGG, and TFs

The top five Gene Ontology (GO) terms in biological process (BP), molecular function (MF), and cellular component (CC) and Kyoto Encyclopedia of Genes and Genomes (KEGG) terms in each category were recognized from functional analysis of upregulated and downregulated genes, as shown in Tables S1 and S2. The most significant enrichment function analysis terms for upregulated genes are cell division for BP, cyclin-dependent protein kinase holoenzyme complex for CC, and cyclin-dependent protein serine/threonine kinase regulator activity for MF, as shown in Table S1. Moreover, the most significant enrichment functional analysis terms for downregulated genes are cell division for BP, spindle for CC, and ATP binding for MF, as shown in Table S2. The KEGG pathway functional analysis indicated that these upregulated and downregulated genes, as shown in Tables S1 and S2, were significantly enriched in cancer, prostate cancer, cell cycle, and p53 signaling pathways. TF prediction analysis was performed by database for annotation, visualization, and integrated discovery (DAVID.6.8). In Table 1, we can see the top 10 TFs associated with upregulated and downregulated DEGs. The five most significant TFs, namely MYC, NFY, STAT, CEBP, and YY1, were found to be associated with upregulated genes, while MYC, NFY, NFAT, TATA, and IRF7 were identified as the significantly associated TFs for the downregulated genes. Their significance was determined using a False Discovery Rate (FDR) and *p*-value < 0.05, as shown in Table 1. MYC (MYC Proto-Oncogene, BHLH Transcription Factor) and NFY (Nuclear transcription factor Y) were the most significantly associated TFs with regulated genes.

### 2.3. Protein–Protein Interaction (PPI) Networks and Module Selection Analysis

**Figure 2:**
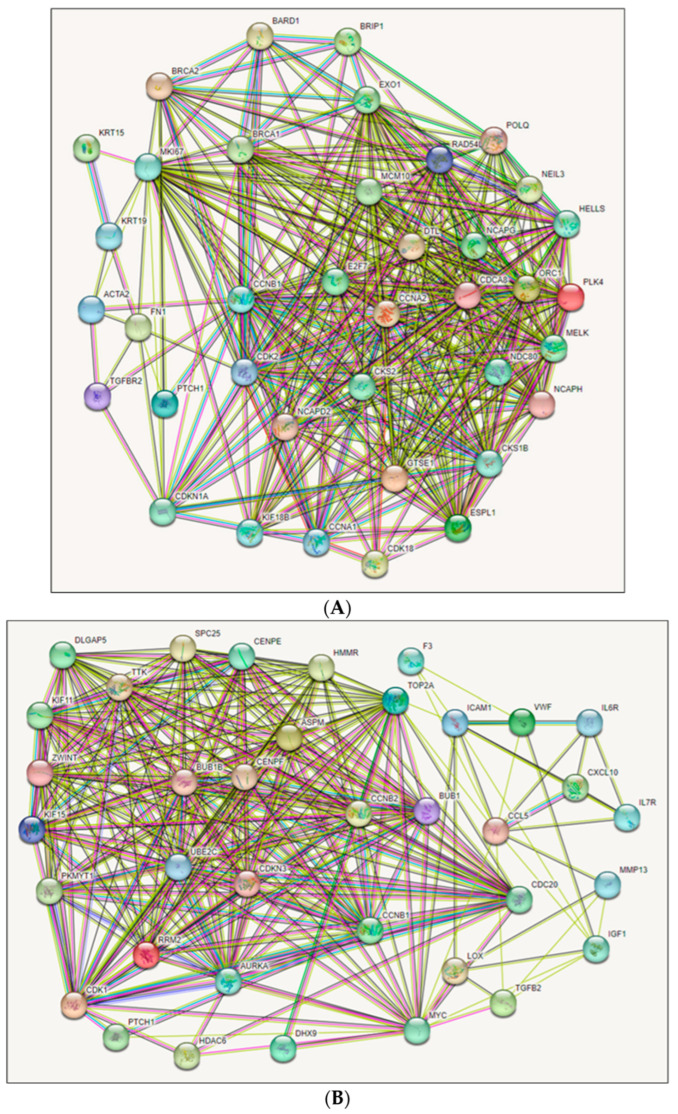
PPI network analysis of 75 DEGs using STRING. (**A**) features a network of 38 upregulated genes (nodes) with 366 edges, an average node degree of 19.3, and a local clustering coefficient of 0.78. (**B**) shows a network of 37 downregulated genes with 282 edges, an average node degree of 15.2, and a local clustering coefficient of 0.88. These networks are derived from RNA-seq datasets with PPI enrichment *p*-value < 1.0^−16^.

PPI networks are critical in comprehending gene functions. Therefore, we constructed PPI networks for DEGs for 38 upregulated and 37 downregulated genes separately to analyze them using the Retrieval of Interacting Genes/Proteins (STRING) database tool. For upregulated genes, there were 38 nodes (genes), 366 edges, an average node degree of 19.3, and a local clustering coefficient of 0.78, as shown in Figure 2A. For downregulated genes, there were 37 nodes (genes), 282 edges, an average node degree of 15.2, and a local clustering coefficient of 0.88, as shown in Figure 2B. Both networks expressed PPI enrichment at *p*-value < 1.0^−16^, which was statistically significant. PPI network modules were generated by Molecular Complex Detection (MCODE). There are four modules of the PPI network identified from RNA-seq. For upregulated genes, Module-1 was connected with a score of 16.3 and contained 25 nodes/genes and 612 edges, and Module-2 was associated with a score of 2.4, including 3 nodes/genes with 3 edges, as shown in Supplementary Figure S3A. ACTA2 and FN1 were identified as clustered and TGFBR2 as seed (highlighted with square shapes) in the clustering analysis in Module-2. In terms of downregulation, Module-1 was connected with a score of 17.6 and contained 23 nodes/genes and 413 edges, and Module-2 was associated with a score of 4.0, including 7 nodes/genes with 18 edges, as shown in Figure S3B. TGFB2, CCL5, CXCL10, ICAM1, IGF1, and IL6R are described as clustered and IL7R as seed (highlighted with square shapes) in the clustering analysis in Module-2. The genes recognized in the Module-2 network, containing three (Figure S3A) and seven (Figure S3B) clustered proteins, were represented as significant genes for the subsequent analysis. The MCODE research for Module-2 of the top clusters containing essential proteins is underlined in the node and responsible for generating the clusters.

### 2.4. Hub Gene Identification

**Figure 3:**
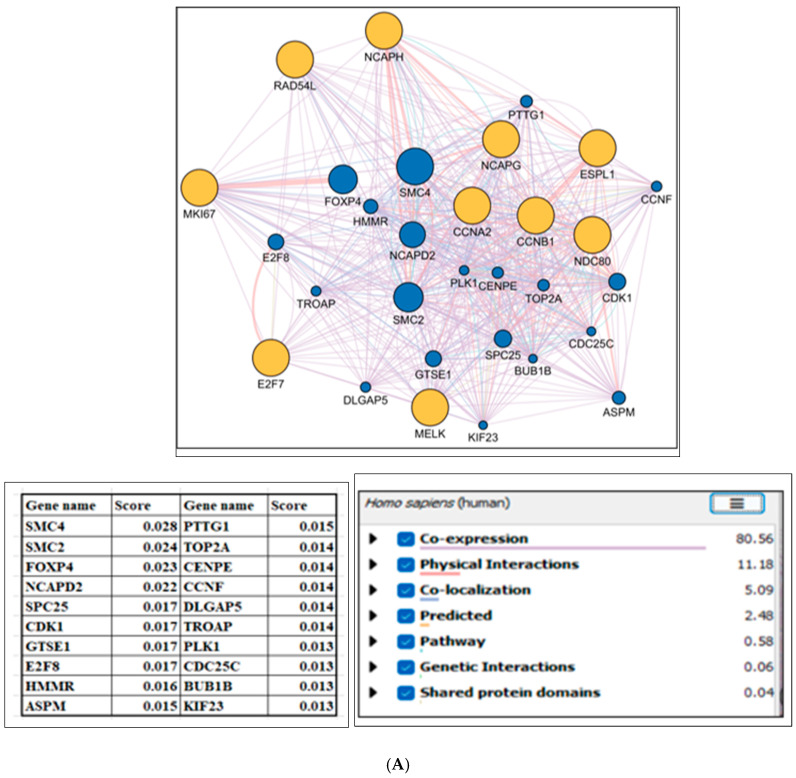

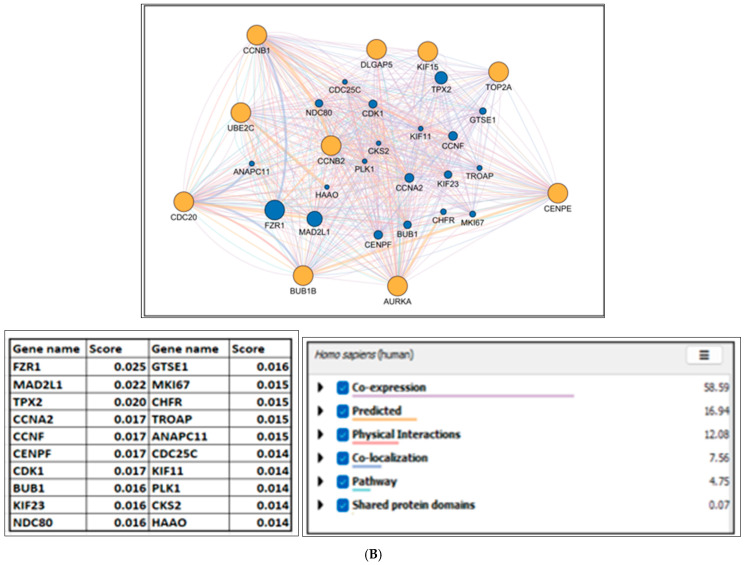
The figure illustrates the GeneMANIA-generated networks of hub genes (orange circles), their associated genes (blue circles), and the weight percentages of various interaction types (physical, co-expression, co-localization, genetic interaction, pathway, and shared protein domain). (**A**) shows the networks of 20 upregulated hub genes, and (**B**) shows 20 downregulated hub genes, each ranked and sized according to their positive real-valued link weight score.

Hub genes were identified based on the CytoHubba topological measures MCC (maximal clique centrality), DMNC (density of maximum neighborhood component), MNC (maximum neighborhood component), Degree, EPC (edge percolated component), and (MCC ꓵ DMNC ꓵ MNC ꓵ Degree ꓵ EPC), as illustrated in Table 2 and Table 3. The hub genes were screened from the PPI network utilizing the CytoHubba tool’s degree rank technique. The topological data of the top 10 upregulated group of genes (NCAPH, RAD54L, E2F7, MKI67, NDC80, MELK, ESPL1, CCNA2, NCAPG, CCNB1) and top 10 downregulated group of genes (TOP2A, BUB1B, CCNB2, AURKA, UBE2C, CCNB1, KIF15, DLGAP5, CENPE, CDC20) are visualized in networks, as shown in Supplementary Figures S4A and S4B, respectively. To distinguish the identified genes based on CytoHubba topological measures, they were categorized into upregulated and downregulated hub gene groups for further analysis. In the next step, to explore the potential mechanism of gene interaction networks in PCa, we used GeneMANIA to generate gene-related PPI networks. The network of hub genes is displayed in an orange circle, and associated genes are expressed in a blue circle, as shown in Figure 3. GeneMANIA indicated that the 10 upregulated hub genes are linked with 20 genes, which are ranked based on their respective scores (SMC4:0.028, SMC2:0.024, FOXP4:0.023, NCAPD2:0.022, SPC25:0.017, CDK1:0.017, GTSE1: 0.017, E2F8:0.017, HMMR:0.016, ASPM:0.015, CKS2GTSE1:0.015, TOP2A:0.014, CENPE:0.014, CCNF:0.014, DLGAP5:0.014, TROAP:0.014, PLK1:0.013, CDC25C:0.013, BUB1B:0.013, KIF23:0.013) (Figure 3A). Similarly, downregulated hub genes are linked with 20 genes (FZR1:0.025, MAD2L1:0.022, TPX2:0.020, CCNA2:0.017, CCNF: 0.017, CDK1:0.017, CENPF:0.017, BUB1:0.016, KIF23:0.016, NDC80:0.016, GTSE1:0.016, MKI67:0.015, CHFR:0.015, TROAP:0.015, ANAPC11:0.015, CDC25C:0.014, KIF11:0.014, PLK1:0.014, CKS2:0.014, HAAO:0.014) as shown in Figure 3B.

### 2.5. Genetic Alteration and Expression of Hub Genes

**Figure 4:**
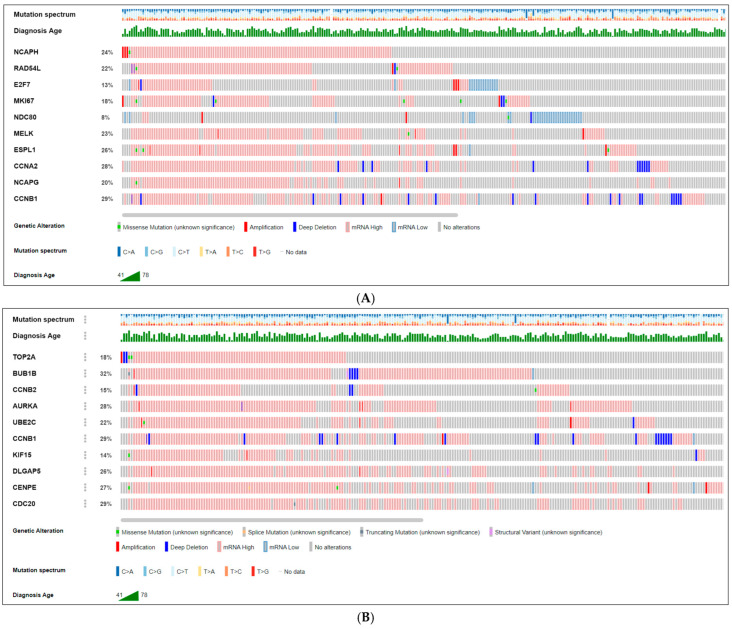
Genetic alteration analysis of hub genes from CBioPortal. The figure summarizes genetic alterations in upregulated (**A**) and downregulated (**B**) hub genes based on data from CBioPortal, encompassing 26 studies with 10,998 samples. Genetic alteration: red is amplification, blue is deep deletion, light red is high mRNA, light blue is low mRNA, and gray is no alteration. Mutation spectrum: adenine (A), cytosine (C), guanine (G), and thymine (T).

**Figure 5:**
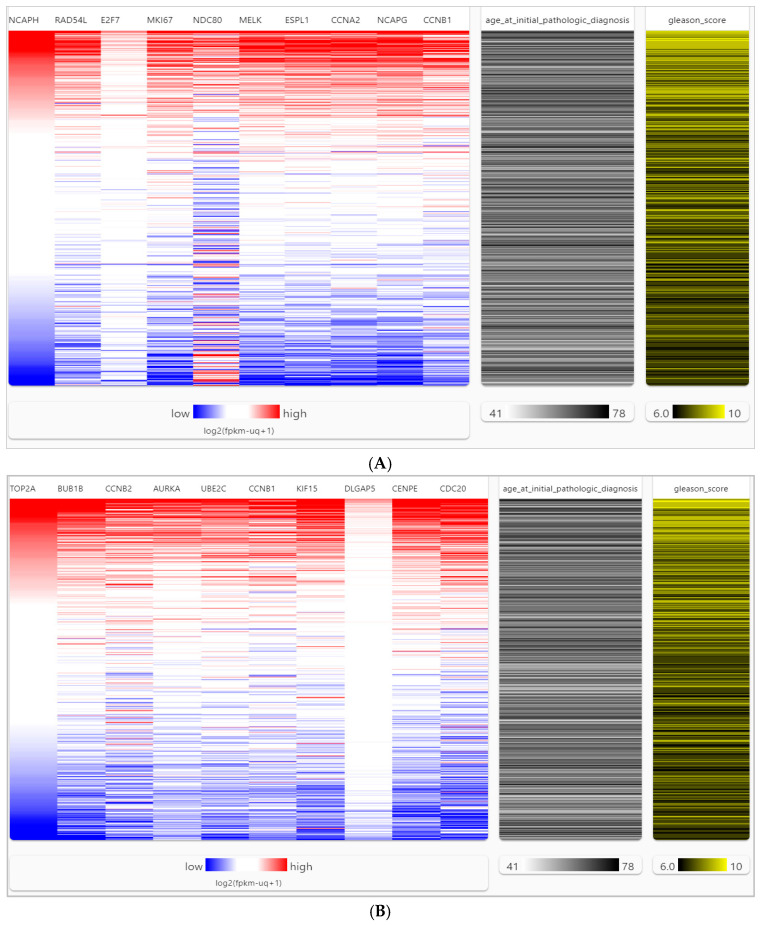
Heatmap analysis of 20 hub genes from TCGA, ICGC, and GTEx. The heatmap visualizes the expression of 20 hub genes, with data sourced from TCGA, ICGC, and GTEx via UCSC-Xena. Color saturation levels correspond to maximum and minimum log2-transformed expression values (red for high expression, blue for low). Gleason scores range from 6 (black) to 10 (yellow), and ages range from 41 (dark gray) to 78 (light gray). Panel (**A**) represents the 10 upregulated and Panel (**B**) represents the 10 downregulated groups of hub genes.

We analyzed the genetic alterations (such as missense mutations, amplifications, deep deletions, high and low mRNA expressions, and no alterations) of each hub gene in predicted networks using the Oncoprint element of the cBio Cancer Genomics Portal (cBioPortal) across 26 PCa studies. This analysis included a total of 10,998 samples. It was found that some genetic alterations affected the upregulated hub genes in a certain percentage of PCa samples. The percentage of affected samples is shown in parentheses next to each upregulated hub gene. The affected upregulated hub genes are CCNB1 (29%), CCNA2 (28%), ESPL1 (26%), NCAPH (24%), RAD54L (22%), MELK (23%), NCAPG (20%), MKI67 (18%), E2F7 (13%), and NDC80 (8%). A visual summary of genetic alterations is presented in Figure 4A. Similarly, some genetic alterations affected the downregulated hub genes in a certain percentage of PCa samples. The percentage of affected samples is shown in parentheses next to each downregulated hub gene. The affected downregulated hub genes are BUB1B (32%), CDC20 (29%), CCNB1 (29%), AURKA (28%), CENPE (27%), DLGAP5 (26%), UBE2C (22%), TOP2A (18%), CCNB2 (15%), and KIF15 (14%). A visual summary of genetic alterations is presented in Figure 4B. Upregulated BUB1B and downregulated CCNB1 hub genes exhibit mutations in 32% and 29% of PCa samples, respectively, indicating their high prevalence. Furthermore, genetic alterations such as high amplification and deletion coincide with these mutations in the PCa samples of the oldest age groups, as depicted in Figure 4. The differential expression of hub genes in PCa and normal prostate tissues was analyzed using the University of California Santa Cruz Xena (UCSC-Xena) database (accessed on 17 November 2022) with data obtained from TCGA (The Cancer Genome Atlas), MSK (Memorial Sloan Kettering Cancer Center), ICGC (International Cancer Genome Consortium), GDC (Genomic Data Commons), and GTEx (Genotype-Tissue Expression), as shown in Figure S5. The gene expression was measured using RNA-seq RSEM, and the norm_count was used for analysis. The data was log2(fpkm-uq + 1) z-transformed, with the Y unit as log2(norm_count + 1), including Y data linear transform by subtracting the mean and dividing std dev (z-score). The analysis confirmed that 20 hub genes between PCa and normal prostate tissues were significantly altered in PCa tissues (*p*-value < 0.05) as shown in violin plots of upregulated and downregulated genes in Figure S5. Hierarchical clustering in a heatmap of hub genes could distinguish PCa samples from normal samples, as shown in Figure 5. The heatmap of the hub genes in PCa clusters showed significant expression compared to the non-cancer groups by UCSC-Xena (Figure 5A,B). Also, the hub genes were highly expressed as shown by a high Gleason score (Figure 5). The results were adjusted, and the color parameters were modified: max color 100% saturation of log2(norm_count + 1) = 12.6 (red) and min color 100% saturation of log2(norm_count + 1) = 2.89 (blue). After comparing PCa samples with normal tissues, the results indicated that upregulated (CCNB1, CCNA2, ESPL1, NCAPH, RAD54L, MELK, NCAPG) and downregulated (BUB1B, CDC20, CCNB1, AURKA, CENPE, UBE2C, TOP2A, CCNB2) genes were associated with PCa carcinogenesis, high Gleason scores ≥ (4 + 3), and age ≥ 55 years.

### 2.6. Hub Gene and Immune Cell Infiltration Estimations

The tumor-infiltrating immune cells are shown to be associated with prognosis and response to different functions and compositions in cancer, including in various stages of PCa [10]. To investigate the relationship between hub gene expressions and tumor-infiltrating immune cells in PCa tissues, we utilized the Tumor Immunity Evaluation Resource (TIMER2.0) database (accessed on 27 November 2023) to analyze the association of hub gene expressions and the levels of various immune cell infiltrations. The results indicate a positive association (*p* < 0.05) between all the upregulated groups of hub genes and the following immune cell populations: CD8+ T cells (RAD54L, E2F7), CD4+ T cells (NCAPH, RAD54L, E2F7, NDC80, MELK, ESPL1, MKI67, CCNA2, NCAPG), B cells (MELK, NDC80), neutrophils (NCAPH, RAD54L, E2F7, NDC80, MELK, ESPL1, MKI67, CCNA2, NCAPG, CCNB1), and macrophages (NCAPH, RAD54L, E2F7, NDC80, ESPL1, CCNA2). These results are illustrated in Figure S6A. The results also indicate that all the downregulated groups of hub genes were positively associated (*p*-value < 0.05) with the following immune cells: CD4+ T cells (TOP2A, CCNB1, AURKA, UBE2C, KIF15, RAD54L, CENPE, CDC20), neutrophils (TOP2A, CCNB1, CCNB2, AURKA, UBE2C, CCNB1, KIF15, RAD54L, CENPE, CDC20), and macrophages (TOP2A, AURKA, KIF15, CENPE). These findings are depicted in Figure S6B. We discovered that several hub genes were significantly associated with immune cells based on partial Spearman’s correlation and a *p*-value <0.05. The genes for neutrophils included NCAPH (0.344, *p*-value = 5.5 × 10^−13^), E2F7 (0.391, *p*-value = 1.18 × 10^−16^), ESPL1 (0.355, *p*-value = 2.25 × 10^−12^), MKI67 (0.298, *p*-value = 5.3 × 10^−10^), CCNA2 (0.277, *p*-value = 8.93 × 10^−9^), TOP2A (0.279, *p*-value = 6.43 × 10^−10^), NCAPG (0.1.93, *p*-value = 7.19 × 10^−5^), CCNB1 (0.198, *p*-value = 4.71 × 10^−5^), BUB1B (0.225, *p*-value = 3.4 × 10^−6^), CCNB2 (0.154, *p*-value = 1.6 × 10^−3^), AURKA (0.201, *p*-value = 3.79 × 10^−5^), KIF15 (0.287, *p*-value = 2.5 × 10^−9^), CENPE (0.376, *p*-value = 1.99 × 10^−15^), and CDC20 (0.178, *p*-value = 2.62 × 10^−4^). For CD4+ T cells, the genes were NDC80 (0.315, *p*-value = 5.29 × 10^−11^), CCNA2 (0.272, *p*-value = 1.64 × 10^−8^), UBE2C (0.132, *p*-value = 7.15 × 10^−3^), KIF15 (0.258, *p*-value = 9.62 × 10^−8^), and DLGAP5 (0.257, *p*-value = 1.01 × 10^−7^). Lastly, for macrophages, the gene was ESPL1 (0.311, *p*-value = 9.3 × 10^−11^). The results also showed that NCAPH, MELK, CCNA2, NCAPG, and KIF15 gene expressions were associated with overall higher infiltration of immune cells to PCa. However, they negatively correlated with CD8+ T cells (Figure S6). The results demonstrate the influence of identified hub genes on the immune microenvironment of PCa.

We utilized TIMER2.0 on TCGA, ICGC, GTEx, and GDC to analyze the differential expression of hub genes in relation to prostate cancer progression. To determine the association of these genes with overall survival (OS), we used Kaplan–Meier curves, as shown in Figure S7. For survival analysis, we used a model that included immune cell infiltrations, gene expression, and the TIMER algorithm. The results indicated that all genes of the upregulated and downregulated groups were significantly associated (*p* < 0.05, Hazard Ratio, Z-score) with immune cell infiltrations (CD8+ T cells, CD4+ T cells, B cells, and macrophages), except for CCNA2, as demonstrated in Figure S7A,B. Specifically, RAD54L, E2F7, MKI67, NDC80, and MELK from the upregulated group and TOP2A, BUB1B, CCNB1, UBE2C, and CCNB1 from the downregulated group were found to be the most highly associated with immune cell infiltrations.

### 2.7. GlueGO and GluePedia

GlueGO and GluePedia were employed to visualize the enrichment results for hub genes. It was discovered that specific hub genes are associated with numerous pathways, potentially regulating the PCa prognosis. The results show that the genes of the upregulated group (CCNB1, ESPL1, MKI67, NDC8) are associated with regulation of chromosome segregation, negative regulation of sister chromatid segregation, mitotic sister chromatid separation, metaphase/anaphase transition of the mitotic cell cycle, and regulation of mitotic metaphase/anaphase transition, as shown in Figure S8. In addition, the results also indicate that genes of the downregulated group (BUB1B, CDC20, CCNB1, AURKA, CENPE, DLGAP5, UBE2C, and TOP2A) are associated with positive regulation of mitotic nuclear division and ubiquitin–protein ligase activities, which are involved in the regulation of mitotic cell cycle transition, chromosome separation, the establishment of chromosome localization, mitotic sister chromatid segregation, anaphase-promoting complex, metaphase/anaphase transition of the mitotic cell cycle, regulation of mitotic metaphase/anaphase transition, and negative regulation of ubiquitin–protein ligase activity involved in the mitotic cell cycle, as shown in Figure S8.

### 2.8. Hub Gene Redundancy and Dependency Analysis

Based on the Cancer DepMap results, eight genes are considered essential in both CRISPR/Cas9 and RNAi, and four genes are unique and essential only in CRISPR/Cas9. Four of these genes (NDC80, CCNA2, NCAPG, ESPL1) from the upregulated group are common to both CRISPR/Cas9 and RNAi and can be potential diagnostic biomarkers and therapeutic targets. Meanwhile, four other genes (CCNB1, MKI67, NCAPG, E2F7) from the downregulated groups are unique to CRISPR/Cas9, which can also be potential diagnostic biomarkers and therapeutic targets. These findings are shown in Figure S9A,B. The predictability analysis has shown that certain genes are highly important for the Core Omics module. The genes that belong to the upregulated group, including NCAPH (3.2%), E2F7 (4.7%), NDC80 (5.9%), and NCAPG (2.1%), as well as the genes from the downregulated group, such as TOP2A (2.8%), BUB1B (3.1%), AURKA (1.0%), UBE2C (2.5%), and DLGAP5 (1.7%), appear to be essential genes. The predictability analysis has identified NDC80 (5.9%) and UBE2C (13.2%) as the genes with the highest relative importance; these findings are shown in Figure S10.

### 2.9. Enrichr and Hub Gene Enrichment Analysis

Enrichr (v3.1) and ggplot2 R packages (v 3.5.1) were used to highlight the functions and pathways of hub genes. The results showed that the upregulated genes in PCa were involved in the cell cycle, oocyte meiosis, the p53 signaling pathway, the FoxO signaling pathway, mitotic sister chromatid segregation, positive regulation of chromosomal regulation, condensed chromosomes, and cyclin-dependent protein serine/threonine kinase regulator activity (Figure S11A). The downregulated genes were associated with the cell cycle, mitosis, mitotic metaphase/anaphase transition, positive regulation of mitotic nuclear division, and microtubule cytoskeleton microtubule binding (Figure S11B).

### 2.10. Regulatory TFs and Their Associated Network with Hub Genes

**Figure 6:**
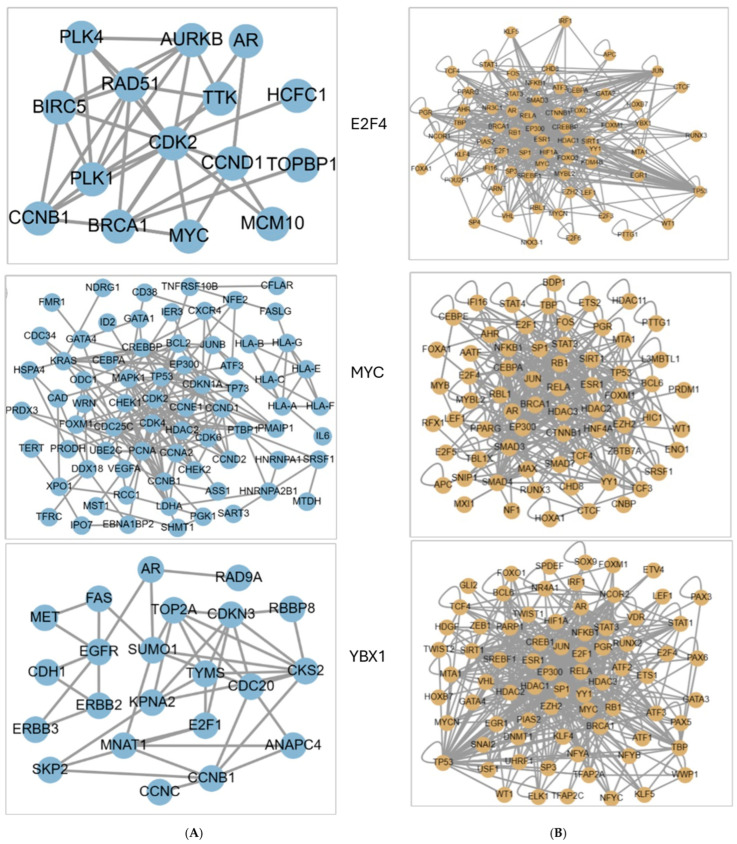
Network construction of protein-encoding TF genes using TRRUST V.2 database. (**A**): Functional E2F4, MYC, and YBX1 gene networks in HumanNet, constructed through modified Bayesian integration. (**B**): Protein–protein interaction (PPI) networks involving E2F4, MYC, and YBX1, mapped using iRefIndex.

The TRRUST V.2 database was used (accessed on 27 November 2023) to identify the critical regulatory TFs associated with hub genes. Table S3 displays the top ten TFs associated with hub genes of the upregulated group, which are E2F4, MYC, RBL1, E2F3, ARNT, YBX1, IRF1, ESR1, E2F1, SP1, and TP53. In contrast, Table S4 shows the top ten TFs associated with genes of the downregulated group, which are YBX1, MYC, E2F4, MED1, E2F3, E2F1, TP53, OTX2, PTTG1, and IRF1. YBX1, MYC, and E2F4 are the three unique TFs associated with both the upregulated and downregulated groups of genes. The HumanNet functional gene network validated protein-encoding genes constructed by a modified Bayesian integration and revealed that E2F4 is associated with 26 genes, 6 of which were discovered in this study (AR, AURKB, CCNB1, CCND1, MYC, TOPBP1) (Figure 6A). Similarly, MYC is associated with 113 genes; 5 genes (CCNA2, CCNB1, CDC20, CCND2, UBE2C) were discovered in this study, and YBX1 is associated with 33 genes; 6 genes (AR, CCNB1, CDC20, CCND2, E2F1, TOP2A) were discovered in this study, as shown in Figure 6A. Figure 6B displays the PPI network through iRefIndex among E2F4, MYC, and YBX1. The diseases/pathways associated with E2F4, MYC, and YBX1 are malignant neoplasm of the prostate (DOID:10283) and prostate carcinoma (DOID:10286) and show the highest significance (*p*-values 6.53^−11^ and 5.20^−11^, respectively), as shown in Table S5. The KEGG pathways and diseases connected with the specified E2F4, MYC, and YBX1 (KEGG term: Term ID: *p*-value) are as follows: (cell cycle: hsa04110: 2.20^−9^), (pathways in cancer: hsa05200: 9.05^−9^), (FoxO signaling pathway: hsa04068: 3.95^−6^), (prostate cancer: hsa05215: 2.14–6), (PI3K-Akt signaling pathway: hsa04151: 5.68^−6^), and (p53 signaling pathway: hsa04115: 1.92^−5^). This is shown in Table S6.

### 2.11. Comparison of Phenotypes for Hub Genes and TFs

The results from Phenolyzer demonstrated that the MYC (TF) has the most significant associations with PCa cases, and the hub genes of the upregulated group (RAD54L, E2F7, CCNB1, CCNA2) and the downregulated group of genes (TOP2A, CDC20, UBE2C, BUB1B, CCNB2, AURKA) as shown in Figure S12. SIGnaling Network Open Resource (SIGNOR 3.0) was used to construct a causal network by connecting the hub genes that have been associated with PCa and mutated in patients, as shown in Figure S13. The results show 27 Seed Entities with the most genomic alterations in PCa and included hub genes (AR, UBE2C, TOP2A, UBE2C, CCNA2, CENPE), TFs (MYC, E2F4, YBX1), and signaling pathways (PI3K-AKT, PIP3, P53, FoxO).

## 3. Discussion

PCa is one of the most common cancers and the principal cause of cancer mortality in men in the USA [1,2,3]. However, until now, confirmed biological/molecular biomarkers, molecular pathways, and signaling pathways underpinning PCa prognosis and development have yet to be completely understood [14,15,16,17,18,19,20,21]. In addition, the complex interacting risk factors for PCa include age, race/ethnicity, family history of PCa, Gleason score, diet, and environmental pollutants such as EDCs mimicking hormones of the endogenous endocrine system [23,24,25,26,27,28,29,30,31,32,33,34,35,36,37,38,39]. There are only a handful of molecular pathological studies that investigate gene–environment interaction to assess the contributions of environmental risk factors, including EDC or xenobiotic exposures, that render PCa cases more aggressive. Our earlier findings have implicated environmental phenols, parabens, and EDCs with a set of hub genes and their TFs in poor PCa prognosis using NHANES and the comparative toxicogenomic databases [34,35]. Therefore, in this study, we expanded the scope to validate the hub genes and their TFs by applying available PCa RNA-seq datasets. RNA-seq datasets offered a real-time snapshot of interacting genes and their TFs with the environmental risk factors, including EDCs, in the aggressive PCa pathways. We included 15 RNA-seq datasets from PCa cell lines representing synthetic androgen R1881 treatment, exposure to EDCs, the role of MYC (TF), high-grade Gleason scores ≥(4 + 3), different ages, and race/ethnicity. Using our approach, we have attempted to capture the heterogeneity of prostate cancer cells comprehensively. This helped us unravel the association of selected hub genes and secondary genes and associated TFs, as well as their alignment in the carcinogenic signaling pathways leading to aggressive PCa prognosis.

We collected gene expressions from RNA-seq dataset profiles from the Expression Omnibus database/National Center for Biotechnology Information (GEO/NCBI) database, (accessed on 27 November 2023) which contained 364 samples: 272 tumors and 92 normal prostate tissue samples. A total number of 10,241 DEGs were obtained; the overlapping genes among the GSE cell lines + R1881, EDC exposure datasets, Gleason scores + MYC (TF), and age/race/ethnicity are 418, 116, 2235, and 7472 genes, respectively. The most significant common genes were numbered at 75 (37 upregulated and 38 downregulated), which were determined as hub genes by CytoHubba topological measures for further analysis (Table 1 and Table 2). The GO enrichment analysis of upregulated and downregulated DEGs demonstrated that GO terms BP, MF, and CC were principally involved in cell division, mitotic cell cycle phase transition, DNA repair, mitotic sister chromatid segregation, and cellular response to ionizing radiation [40]. In addition, mitotic failure can potentially be a significant mechanism of immediate DNA damage [41]. These DEGs also included the selected EDC-associated hub genes from our earlier studies [34,35]. In the KEGG pathway analysis, the top five connected pathways aligned with cancer, prostate cancer, the cell cycle, the p53 signaling pathway, and the FoxO signaling pathway. KEGG enrichment analysis reveals that DEGs are involved in the main pathways of cancer, prostate cancer, the cell cycle, the p53 signaling pathway [42], and the FoxO signaling pathway [43]. Consequently, all the above functional analyses and alignment of DEGs in pathways verified that our results are associated with the progression of PCa. The most significant TFs identified for upregulated and downregulated genes are Nuclear transcription factor Y (NFY) and MYC [35]. We demonstrate that MYC overexpression significantly decreases AR transcriptional signaling, which impacts the targeted genes controlled by the AR protein in prostate luminal cells [44,45].

We visualized numerous genomic alteration possibilities of the hub genes across a set of PCa samples utilizing OncoPrint employing a query for alterations. Most of the alterations were identified as amplifications and deep deletions (Figure 4). Recurrent abnormalities in genes and pathways related to PCa cancer were identified. The most expected mutations are in CCNB1 (29%) and BUB1B (32%). This suggests that amplification- and deletion-based mutations were associated with hub gene overexpression in PCa tissues, and modifications were noticed in the main domains of hub genes. Higher expression levels of CCNB1 in PCa tissues may indicate its influence on poor prognosis and its role as a better candidate as a biomarker for aggressive PCa [46]. BUB1B, identified as one of the EDC-associated hub genes, is an essential mitotic checkpoint kinase determined as the top-scoring kinase by RNA transcription. BUB1B is a protein that plays a crucial role in cell division, specifically ensuring accurate chromosome segregation during mitosis. It is an element of the mitotic spindle checkpoint. This mechanism prevents cells from dividing when there are errors or abnormalities in the alignment of chromosomes on the spindle fibers [47,48]. Chromosomal instability, caused by mutations or dysregulation by EDC exposure to genes like BUB1B, can contribute to the progression or poor prognosis of PCa.

It is understood that cancers are formed not only by cancer cells but also by stroma and immune cells [49]. Immune cells play a crucial role in the development and progression of PCa. PCa can trigger different immune responses in the body, and the interactions between immune and cancer cells are complex. If detected, immune cells such as T cells can identify and attack cancer cells. The immune cells can infiltrate the PCa. Their presence within the cancer microenvironment can have both positive and negative outcomes. They can help prevent cancer growth by identifying and attacking cancer cells. On the other hand, cancers can create mechanisms to evade immune detection, leading to an insufficient immune response [18]. Lately, lymphocytic immune cell infiltration in PCa tissue has been presented as an immune mechanism of PCa progression. Therefore, targeted immune cell movements can be a possible early diagnostic opportunity for PCa. Our study indicates a significant positive prognostic influence of upregulated (NCAPH, RAD54L, E2F7, NDC80, MELK, ESPL1, MKI67, CCNA2, NCAPG) and downregulated (TOP2A, CCNB1, CCNB2, AURKA, UBE2C, CCNB1, KIF15, RAD54L, CENPE, CDC20) genes associated with the majority of the tumor-infiltrating immune cells, also indicating the above genes as potential biomarkers which are primarily expressed in PCa cells. The current study revealed that an increase in neutrophils, macrophages, and T cell populations was also associated with RAD54L, E2F7, MKI67, NDC80, MELK, TOP2A, BUB1B, CCNB1, UBE2C, and CCNB1 in PCa cell lines. These increased immune cells connected with the above genes are speculated to be involved in the progression of PCa and the OS of the patients (Figure S7).

The above results are compatible with other research findings [10,49]. GlueGO and GluePedia enrichment analysis results showed that CCNB1, ESPL1, MKI67, NDC8, BUB1B, CDC20, CCNB1, AURKA, CENPE, DLGAP5, UBE2C, and TOP2A genes were associated with cell cycle and apoptosis, activation of the p53 signaling pathway, tumor suppression, and cell cycle arrest [50,51], alongside a mitotic checkpoint serine kinase that has been documented in PCa [52]. Furthermore, some of these immune cells’ infiltration-associated hub genes and TFs have been shown to be EDC-associated [34,35].

The redundancy analysis showed that the genes NDC80, CCNA2, NCAPG, ESPL1, OP2A, BUB1B, AURKA, and CDC20 may be prognostic indicators of PCa. These eight genes are also differentially expressed in PCa cell lines by protein-level validation. The nine genes NCAPH, E2F7, NDC80, NCAPG, TOP2A, BUB1B, AURKA, UBE2C, and DLGAP5 are essential in both CRISPR/Cas9 knockout and the RNAi predictability of relative importance, suggesting that the above genes are unique and have potential to be diagnostic biomarkers for PCa cell lines [53]. The enrichment analysis for hub genes for PCa shows that the upregulated genes were mainly affected in the cell cycle, oocyte meiosis, the p53 signaling pathway, and the FoxO signaling pathway, which are all closely associated with cancer and PCa [40,41,49]. The downregulated hub genes were particularly enriched in the Cell cycle, mitotic cell cycle, regulation of mitotic metaphase/anaphase transition, and positive regulation of mitotic nuclear division, which are reported in different cancers, including PCa [54,55].

The key regulatory TFs identified from the TRRUST V.2 database and associated with both upregulation and downregulation of hub genes are YBX1, MYC, and E2F4. In PCa, MYC is overexpressed in earlier phases of the disease and functions as a critical driver of disease progression and tumorigenesis [54]. MYC overexpression is marked in up to 40% of metastatic PCa patients [55] and is connected with poor survival [56]. E2F4 is enriched in differentiated and nonproliferating cells and recreates essential functions in suppressing proliferation-associated hub genes [57]. The level of E2F4 protein was increased significantly in the nuclei of PCa cells [58] and acted as a transcriptional suppressor by reducing TGF-β in the survival expression in PCa epithelial cells [59]. YBX-1 controls AR expression, transcriptionally associated with a splicing mechanism [60]. Thus, YBX-1 seems to be an effective target to inhibit the growth of PCa even at the AR expression stage, where novel AR-targeting agents and medicines are inefficient [61]. The identified YBX1, MYC, and E2F4 TFs were associated mainly with DOIDs (malignant neoplasm of the prostate and prostate carcinoma) and KEGG pathways for disease-enriched cell cycle, pathways in cancer, the FoxO signaling pathway, prostate cancer, the PI3K-Akt signaling pathway, and the p53 signaling pathway, which are considered to be associated with cancer prognosis. Causal interaction analysis of hub genes in DisNor showed 27 Seed Entities with the most genomic alterations in PCa impact. The most significant findings are validated with our results for genes UBE2C, TOP2A, UBE2C, CCNA2, and CENPE, as well as TFs (MYC, E2F4, YBX1) and signaling pathways (PI3K-AKT, PIP3, P53, FoxO).

While acknowledging the limitations of our research, we interpret our results with caution. As with our previous studies [34,35], the current study also relies on mining public databases for bioinformatics analysis, and the data quality was not evaluated. Our approach to using the publicly available RNA-seq data allowed us to maximize the sample size and capture the heterogeneous mix of cells representing different origins, stages, and EDC treatments. However, these datasets may be susceptible to batch effects, platform-dependent variability, and RNA library preparation protocols. Publicly available datasets may vary in annotation consistency and quality control, which may have an effect on downstream data processing. We integrated multiple bioinformatics platforms, such as cBioPortal, UCSC Xena, TIMER2.0, and TRRUST, which are essential for building a robust and biologically meaningful analysis. However, using multiple tools increases the risk of redundancy, overfitting, or inconsistent interpretations if not handled carefully. As demonstrated, each tool had a distinct focus to provide non-redundant data types, which support complementary insights rather than duplication. For example, cBioPortal was used for clinical and genomic alteration data (e.g., mutations, CNVs, survival); UCSC-Xena was used for multi-omics visualization (expression, methylation, clinical features); TIMER2.0 was used for immune cell infiltration analysis using transcriptomic data; and TRRUST was used to map the curated transcription factor–target gene regulatory networks. This analysis enabled us to distinguish the type of evidence from each source based on its strength, without overlapping functions.

Our research strongly suggests the potential roles of EDC-associated hub genes in aggressive PCa pathways. By integrating different RNA-seq datasets, we unraveled hub genes and their interactions in a heterogeneous pool of PCa samples, leading to the aggressive PCa form. This observation reinforces the potential of the identified EDC-associated hub genes and their associated TFs as predictive biomarkers for assessing the environmental health risks of EDC exposures and as potential targets for aggressive PCa diagnosis and treatment. We found that some EDC-associated hub genes and TFs aligned with PCa pathways repeatedly appeared in our previous and current analyses [34,35]. This observation positions these hub genes as strong biomarker candidates for EDC risk assessment studies or clinical prospective studies. Addressing the translation of identified hub genes and TFs into clinically actionable biomarkers or therapeutic targets within the context of patient samples or functional assays is beyond the immediate scope of the current manuscript. However, this will be a crucial direction for future research.

## 4. Materials and Methods

We used comprehensive RNA-seq datasets and integrated bioinformatics and statistical procedures to analyze the datasets to investigate the hub genes and TFs based on their function roles(s) in regulating biological pathways and PCa prognoses.

### 4.1. Omics Data Acquisition

To acquire multiple GSE omics datasets for RNA-seq data related to PCa, we accessed and downloaded datasets from GEO/NCBI (http://www.ncbi.nlm.nih.gov/geo, accessed on 15 May 2023 [62]. The datasets incorporated in this research were established on controls following certain benchmarks: (1) the datasets included RNA-seq data only, (2) the origin of the samples was normal prostate or PCa tissue, (3) studies were conducted exclusively in Homo sapiens samples, (4) studies clearly described the sample selection protocol, (5) datasets had publicly available raw data, (6) the dataset included more than six samples (at least three controls and three cases), (7) samples were not treated with medication, radiation, or chemotherapy before sampling, (8) samples were from the USA and European countries, and (9) studies used Affymetrix, Illumina, or Agilent platforms. The following RNA-seq datasets that fit the criteria were identified and downloaded from GEO: GSE70466 [63], GSE151290 [64], GSE128749 [65], GSE120660 [66], GSE135879 [67], GSE136272 [68], GSE218556 [69], GSE64529 [70], GSE5590 [71], GSE128339 [72], GSE109021 [73], GSE200879 [74], GSE103512 [49], GSE104131 [75], and GSE200167 [76]. The characteristics of RNA-seq databases, including platforms, sample size, and description information in this study, are shown in Table 4. The RNA-seq databases were downloaded from GEO for 364 samples, including 272 tumors and 92 normal prostate tissue samples.

### 4.2. Data Processing

DEGs in PCa and control samples were determined by employing GEO2R/DESeq2 (http://www.ncbi.nlm.nih.gov/geo/geo2r, accessed on 15 May 2023) and the R package DESeq2 package (v 1.40.2) [77]. The Benjamini and Hochberg FDR methods were implemented in acquiring DEGs for multiple RNA-seq datasets [78]. We used significance cutoffs to identify the DEGs. The selection threshold for the DEGs was specified based on |log2FC| (fold change) ≥ 2 and *p*-value < 0.05. The RNA-seq datasets of each microarray dataset and across the 15 GSE profiles were normalized for distribution by DESeq2 [77]. We utilized OriginPro 9.1 (scientific data analysis and graphing software) [79] to create volcano plots of the DEGs of up- and downregulated genes. Furthermore, Venn diagrams of the overlapping DEGs were output by using the Venny 2.0 (https://bioinfogp.cnb.csic.es/tools/venny/, accessed on 15 June 2023) [80] and InteractiVenn (http://www.interactivenn.net/, accessed on 15 June 2023) [81] web-based tools.

### 4.3. Statistical Analysis

The GEOquery/DESeq2 package for the R platform was utilized to download the RNA-seq datasets, and the GEOquery R package merges GEO data into R data and R packages (Version. 3.6.0) [82]. Each of the 41 RNA-seq datasets was submitted for normalization, background correction, and summarization. Therefore, the following tools were utilized: Affymetrix-derived datasets—affy [83]; Illumina-derived datasets—lumi; and Agilent with R-package (Version 3.6.0) limma, [84,85]. DESeq2 conducts comparisons on actual submitter-supplied processed data utilizing the R packages GEO-query and linear models for RNA-seq dataset analysis (limma) from the Bioconductor project. Bioconductor provides access to statistical and graphical methods for analyzing genomic data based on the R programming language. The adjusted *p*-values and FDR (Benjamini and Hochberg) were utilized to balance the results of statistically significant DEGs and to reduce the possibility of false-positive errors.

### 4.4. Identification of DEGs and Pathway Enrichment Analysis

DAVID.6.8 (https://david.ncifcrf.gov/, accessed on 15 July 2023) is a pathway enrichment and functional analysis tool for high-throughput sequencing of genomic and proteomic datasets that include BP, CC, and MF [86,87]. DAVID.6.8 was utilized based on the KEGG (https://www.genome.jp/kegg/, accessed on 15 July 2023) [88] and GO (http://www.geneontology.org, accessed on 15 July 2023) [89] analyses of the identified genes, with *p* ≤ 0.05 considered as the cutoff criterion for determining enrichment. Together, the GO and KEGG pathway analyses were used to examine BP, MF, and CC associated with DEGs, their potential enrichment, and their processes in PCa pathways. For creating PPI networks of the DEGs, we used the STRING database (http://string-db.org/, accessed on 15 July 2023) to develop the primary PPI networks (version 11.5 metasearch engine) [90]. The parameter analysis criteria in STRING were a degree of confidence of 0.40, human species, local gene fusion databases, a clustering coefficient of 0.78, and a PPI enrichment *p*-value < 1.0^−16^. STRING functions as an online access point for analyzing associations between mixed genes/proteins on a genome-wide scale, Gene Ontology, clustering, and centralities, which is valuable for understanding the PPI network and functions.

### 4.5. Biological System Analysis and Module Network Mining

Cytoscape software (version 3.10.0), an open-source platform (https://cytoscape.org/, accessed on 15 June 2023) was utilized for module network analysis and selection. Cytoscape provides complex omics datasets through analysis and visualization functions of genes/proteins through enormous and dynamic apps. Also, Cytoscape apps include a network of biological functionality and specialized programming approaches for producing complex and evolving workflows [91]. Therefore, PPI networks were obtained from STRING and imported into the Cytoscape for network and module analysis [92]. Networks were analyzed regarding clustering and GO, aiming to discover the most topologically relevant nodes/genes and molecular pathways associated with PPI networks [93]. A Cytoscape plug-in, Molecular Complex Detection (MCODE), was used to discover significant clustering modules and PPI networks to select intensively connected ones [94]. The following previously defined criteria in MCODE were implemented to determine significant modules in PPI networks: degree cutoff = 2; node score cutoff = 0.2; K-Core = 2; max depth = 100 [95]. To discover the most hub genes in the PPI networks and modules, we implemented the Cytoscape plug-in CytoHubba. CytoHubba scored and ranked the nodes using algorithms based on eleven topological analysis methods for duplicated measurements to strengthen the observation of the interactions [96]. In our study, we retained results with precise prediction regarding MCC, DNMC, EPC, degree, and MNC [35,96]. In the next step, to generate an interaction network to assess hub gene/protein lists from identified modules, GeneMANIA was used to create and rank hub genes and associated genes for functional experiments and molecular interaction networks [97]. GeneMANIA is established on co-expression networks, physical interactions, genetic interactions, co-localization pathways, and predicted and shared protein domain information [98,99]. The top 20 overlapping hub genes based on these five topological methods from CytoHubba were selected for additional bioinformatics analysis utilizing GeneMANIA, which includes a comprehensive set of datasets from BioGRID, GEO, I2D, and Pathway Commons, in addition to nine organisms distinguishing functional genomics datasets [97,98,99]. In our study, we selected all networks and Homo sapiens, as defined previously [35]. Hub genes and TFs identified from the RNA-seq dataset will be implemented for further validation, verification, and evaluation/analysis on various bioinformatics data tools for signature-related genes in the prognosis of PCa.

### 4.6. Genetic Alteration in Hub Genes

The cBioPortal, V 5.3.13, is an open-access online tool (http://www.cbioportal.org, accessed on 30 July 2023) integrating the datasets from large-scale genomic projects including, but not limited to, TCGA, MSK, and ICGC, a resource for examining a multidimensional cancer genomics dataset [100]. The cBioPortal was utilized (according to online instructions) to investigate the visualization and comparison of genetic alterations of the hub genes involved in the signature. The PCa datasets from 26 studies containing 10,998 samples were chosen to analyze the genomic profile changes, retaining mutations, gene alterations (GISTIC: genomic identification of significant targets in cancer), and microarray mRNA expression (Z-scores). Genetic alteration co-occurrence and mutual exclusivity between each enquired hub genes were defined by *p* value, log2 odds ratio, and q value, where a *p* value < 0.05 was considered significant. Oncoprints describing genomic alteration mutations and the number of samples were generated employing a heatmap through cBioPortal Oncoprint [101,102].

### 4.7. Visualizing the Heatmap and Hub Gene Expressions

UCSC-Xena (http://xena.ucsc.edu/ accessed on 30 July 2023) is a cancer genomics visualization tool, and datasets in UCSC-Xena include but are not limited to TCGA, ICGC, GTEx, and GDC. UCSC-Xena was used for hierarchical clustering heatmaps and gene expression of hub genes [103]. For heatmaps, highly expressed genes were calculated by (log2(norm_count + 1), Gleason score [rated from 6 (dark yellow) to 10 (light yellow)], and age [rated from 41 (light gray) to 10 (dark gray)]. Gene expressions were calculated by log2(fpkm-uq + 1) and z-transformed based on the RNAseq RSEM norm_count, and *p* values < 0.05 were viewed as significant [35,103].

### 4.8. Hub Genes’ Association with Immune Cell Infiltration

We used the TIMER2.0 database (http://timer.cistrome.org/, accessed on 30 July 2023) to construct reliable immune infiltration estimations. TIMER2.0, utilizing an R-package (3.6.0), integrates six algorithms that have been systematically benchmarked, including TIMER, xCell, CIBERSORT, MCP-counter, quanTIseq, and EPIC [104]. TIMER2.0 is a comprehensive resource for identifying immune cell infiltration in cancers utilizing RNA-seq expression profiling datasets [105,106]. In the current study, hub genes’ expression was associated with five kinds of immune infiltration (CD4+ T cells, CD8+ T cells, B cells, neutrophils, and macrophages), which were correlated and assessed in the “Gene” module of “TIMER.” In addition, survival analysis was performed by TIMER2.0 for hub genes and immune infiltration cells by applying the following criteria:

Model Survivor analysis = Immune Infiltrates + Gene Expression + TIMMER (algorithm).

Kaplan–Meier curve parameters included a split expression of patients at 50%, a split infiltration of patients at 50%, and time in months. Cox proportional regression hazard mode was used for the model section, and infiltration level was divided into low and high groups with identified HRs; a *p*-value < 0.05 was deemed statistically significant [104,105,106].

### 4.9. ClueGO and CluePedia: Functional Enrichment Analysis

ClueGO (Version 2.5.10) and CluePedia (Version 1.5.10) were employed for functional enrichment analysis and pathway annotation networks for hub genes and TFs. ClueGO and CluePedia present the possibility of estimating enrichment analysis in two-sided tests established based on the hypergeometric distribution and utilized in combination with Bonferroni tests with a significance of *p* < 0.05. ClueGO combines GO terms and KEGG/BioCarta pathways, constructs a functionally categorized GO pathway, and visualizes the network [107,108]. It can analyze one or two lists of genes and comprehensively visualize functionally grouped terms. The parameter was restricted to facilitate the figure and only demonstrate the key pathway.

### 4.10. Redundancy Analyses of Hub Genes

We utilized Cancer DepMap (https://depmap.org/portal/, accessed on 30 July 2023) to determine the functional hub genes critical for PCa prognosis and survival. Cancer DepMap is established on the RNAi and CRISPR-Cas9 knockout databases [109,110]. CRISPR/Cas9 is a gene-editing technology that applies to direct RNA to match a selected target gene and Cas9 enzyme (CRISPR-associated protein-9, an endonuclease), which generates a double-stranded DNA break, allowing transformations to the genome [111]. RNA interference (RNAi) is a technique for silencing gene expression, which is the consequence of the degradation of RNA into short RNAs that activate ribonucleases to target mRNA [112]. DepMap includes cancer types based on their contribution to cancer mortality and their cancer cell line representation in the documented dataset. In our study, we utilized DepMap to (1) conduct redundancy analysis for hub genes, (2) identify the essential function of indicated hub genes in PCa total cell line survival, and (3) identify the CERES dependency score of hub genes in PCa cell lines.

### 4.11. Hub Gene Enrichment Analysis—Enrichr

Numerous experimentally verified online TF networks are available to evaluate TFs’ interaction with hub genes. This study used Enrichr (https://maayanlab.cloud/Enrichr/, accessed on 30 July 2023) to obtain BP, CC, MF, and KEGG pathways and identify TFs [113,114]. Enrichr includes WikiPathways, Chip Enrichment Analysis (ChEA), Reactome, and the BioCarta database [115]. The ggplot2 R package was employed to generate figures for Enrichr analysis. *p*-value < 0.05 was selected as the criterion. 

### 4.12. TF Association Network with Hub Genes

TRRUST Version 2 (https://www.grnpedia.org/trrust/, accessed on 30 July 2023) is an available database of human TFs, gene interactions, and regulatory networks [116]. The TRRUST V.2 database was utilized to obtain the TFs regulating hub genes through BP, KEGG, and disease pathways. The species was selected as *Homo sapiens*, and a *p*-value <0.05 was set as the significance threshold [117]. Moreover, we implemented a functional network of the most significant TFs associated with hub genes via HumanNet, which includes a probabilistic functioning gene network of 19,000 validated protein-encoding genes of Homo sapiens. Each relation in HumanNet has a linked log-likelihood score (LLS) that calculates the probability of an interaction designating a true functional connection between two genes [118]. Finally, we created a PPI network of interactions with the most significant TFs associated with hub genes through iRefIndex, which is a compressed PPI database containing molecular interactions derived from various sources such as BioGRID, BIND, DIP, and HPRD [119]. 

### 4.13. Causal Association and Protein Interactions and Phenotypes with DEGs

Phenolyzer (https://phenolyzer.wglab.org/, accessed on 30 July 2023) was used to perform a cross-sectional comparison of hub genes and phenotypes, which would provide indicators for measuring expression across cancers [120]. Phenolyzer was used to research various forms of PCa—prostatic neoplasms, familial PCa, prostatic hyperplasia, and PCa neoplasia—which are clinical phenotype terms that are essentially related to each other. Then, we implemented SIGNOR 3.0 (https://signor.uniroma2.it, accessed on 30 July 2023), a public dataset of static maps that capture causal information and interactions that can be tailored, pruned and developed to create dynamic and predictive models [119]. Each signaling association is annotated with an effect of up- and downregulation and with the mechanisms in which indices (e.g., transcriptional activation, binding, and phosphorylation) generate the regulation of the target proteins/genes. SIGNOR 3.0 guides several exterior resources that characterize the use of the data in SIGNOR 3.0 to extract biologically suitable information in three biological parts: Disnor (disease pathways and disease-associated genes), Myo-REG (cell signaling interactions), and CancerGeneNet (genes repeatedly mutated in hallmark cancer phenotypes) [121,122].

## 5. Conclusions

In conclusion, the interactions among hub genes, TFs, and environmental risk factors, particularly endocrine-disrupting chemicals (EDCs), reveal a complex interplay that emphasizes the multifactorial nature of prostate cancer (PCa) development and prognosis. To our knowledge, this represents one of the first comprehensive RNA-seq analyses, integrating data from 15 gene expression enrichment (GSE) datasets focused on PCa. These include PCa cell lines treated with synthetic androgens, exposed to selected EDCs, and utilized to explore the role of MYC (a TF) in carcinogenic processes. The dataset captures a heterogeneous range of prostate and PCa cells, including various ages and races, as well as high-grade Gleason scores of ≥7 (4 + 3). Our study employed advanced bioinformatics tools to analyze gene expression profiles through GSE, focused on PCa-related pathways. We validated hub genes (NCAPG, MKI67, CCNA2, CCNB1, CDK1, CCNB2, AURKA, UBE2C, BUB1B) and their associated TFs (MYC, E2F4, YBX1) within the aggressive PCa pathway, particularly among older patients. Survival analysis, aligned with high Gleason scores and age, indicates that these hub genes and their TFs are essential to the cellular signaling pathways implicated in aggressive PCa prognosis. These findings underscore the potential of these hub genes as candidates for future studies aimed at developing molecular biomarkers for predicting and assessing EDC-related PCa risk, diagnosing PCa aggressiveness, and identifying therapeutic targets.

## Figures and Tables

**Table 1:** List of top 10 TFs associated with the number of DEGs identified from RNA-seq datasets for PCa.

Category	#	TFs	# of Genes	*p*-Value	FDR ^2^
Upregulated UCSC_TFBS ^1^	1	MYC	28	6.48 × 10^−5^	0.011
	2	NFY	21	1.03 × 10^−3^	0.086
	3	STAT	33	5.52 × 10^−3^	0.222
	4	CEBP	31	7.13 × 10^−3^	0.222
	5	YY1	34	9.82 × 10^−3^	0.243
	6	EVI1	17	1.16 × 10^−2^	0.266
	7	HSF1	22	1.13 × 10^−2^	0.266
	8	CDPCR3HD	26	1.40 × 10^−2^	0.266
	9	STAT5A	26	1.68 × 10^−2^	0.266
	10	PBX1	19	1.58 × 10^−2^	0.269
Downregulated UCSC_TFBS	1	MYC	22	1.60 × 10^−2^	1.00
	2	NFY	19	2.70 × 10^−2^	1.00
	3	NFAT	23	2.90 × 10^−2^	1.00
	4	TATA	20	3.10 × 10^−2^	1.00
	5	IRF7	28	6.20 × 10^−2^	1.00
	6	MEF2	18	6.60 × 10^−2^	1.00
	7	FREAC3	18	9.10 × 10^−2^	1.00
	8	ISRE	23	9.80 × 10^−2^	1.00
	9	CDP	19	6.80 × 10^−2^	1.00
	10	NF1	18	9.90 × 10^−2^	1.00

^1^ UCSC_TFBS: University of California, Santa Cruz—transcription factor binding sites. ^2^ FDR: False Discovery Rate.

**Table 2:** Ten hub genes were selected based on CytoHubba topological measures for upregulated genes by associating the five most precise and predicted topological measures in the PPI network.

MCC	DMNC	MNC	Degree	EPC	MCC ꓵ DMNC ꓵ MNC ꓵ Degree ꓵ EPC
NCAPH	NCAPH	NCAPH	NCAPH	NCAPH	NCAPH
RAD54L	RAD54L	RAD54L	RAD54L	RAD54L	RAD54L
E2F7	E2F7	E2F7	E2F7	E2F7	E2F7
MKI67	MKI67	MKI67	MKI67	MKI67	MKI67
NDC80	NDC80	NDC80	NDC80	NDC80	NDC80
MELK	MELK	MELK	MELK	MELK	MELK
ESPL1	ESPL1	ESPL1	ESPL1	ESPL1	ESPL1
CCNA2	CCNA2	CCNA2	CCNA2	CCNA2	CCNA2
CKS2	GTSE1	ASPM	CKS2	GTSE1	
ORC1	CHFR	FZR1	ORC1	CHFR	
PLK1	DTL	DTL	ANAPC11	DTL	
MCM10	DTL	MCM10	PLK1	MCM10	
HELLS	HELLS	ANAPC11	HELLS	HELLS	
NCAPG	CCNB1	NCAPG	NCAPG	NCAPG	NCAPG
CCNB1	NCAPG	CCNB1	CCNB1	CCNB1	CCNB1
PLK4	ANAPC11	PLK4	PLK4	FZR1	
HAAO	EXO1	EXO1	FZR1	SMC4	
HAAO	SPC25	FOXP4	FZR1	EXO1	
MCM10	DTL	FOXP4	PLK1	MCM10	
CKS2	GTSE1	ASPM	CKS2	GTSE1	

**Table 3:** Ten hub genes were selected based on CytoHubba topological measures for downregulated genes by associating the five most precise and predicted topological measures with the PPI network.

MCC	DMNC	MNC	Degree	EPC	MCC ꓵ DMNC ꓵ MNC ꓵ Degree ꓵ EPC
TOP2A	TOP2A	TOP2A	TOP2A	TOP2A	TOP2A
CCNB1	CDC20	ASPM	GTSE1	ASPM	
CDC20	BUB1B	CDC20	CDC20	CDC20	CDC20
BUB1B	HMMR	BUB1B	BUB1B	BUB1B	BUB1B
HMMR	TTK	HMMR	HMMR	GTSE1	
RRM2	TTK	RRM2	RRM2	RRM2	
CDK1	BUB1	GTSE1	CDK1	CDK1	
TTK	CENPF	TTK	TTK	GTSE1	
BUB1	CDKN3	BUB1	BUB1	BUB1	BUB1
CENPF	PKMYT1	CENPF	CENPF	CENPF	CENPE
CDKN3	CCNB2	CDKN3	CDKN3	CDKN3	
CCNB2	KIF15	CCNB2	CCNB2	CCNB2	CCNB2
KIF15	AURKA	KIF15	KIF15	KIF15	KIF15
AURKA	KIF11	AURKA	AURKA	AURKA	AURKA
KIF11	DLGAP5	KIF11	KIF11	KIF11	
DLGAP5	ZWINT	DLGAP5	DLGAP5	DLGAP5	DLGAP5
ZWINT	CENPE	ZWINT	GTSE1	ZWINT	
CENPE	SPC25	CENPE	CENPE	CENPE	
UBE2C	UBE2C	UBE2C	UBE2C	UBE2C	UBE2C
ASPM	GTSE1	ASPM	ASPM	GTSE1	

**Table 4:** Overview of the specified RNA-seq dataset linked with PCa included in the study.

Group					Cell Lines			
	#	GEO Profile	Platform	Annotation Platform	Total	PCa	Control	References
Cell lines treated with R1881 treatment	1	GSE70466	GPL16791	Illumina HiSeq 2500	6	3	3	[63]
	2	GSE151290	GPL16791	Illumina HiSeq 2500	16	8	8	[64]
	3	GSE128749	GPL11154	Illumina HiSeq 2000	11	5	6	[65]
	4	GSE120660	GPL16791	Illumina HiSeq 2500	21	12	9	[66]
	5	GSE135879	GPL16791	Illumina HiSeq 2500	12	6	6	[67]
	6	GSE136272	GPL16791	Illumina HiSeq 2500	12	6	6	[68]
Cell lines with EDC exposure	7	GSE218556	GPL24676	Illumina NovaSeq 6000	6	3	3	[69]
	8	GSE64529	GPL11154	Illumina HiSeq 2000	6	3	3	[70]
	9	GSE5590	GPL2986	Illumina HiSeq 2500	6	3	3	[71]
	10	GSE128339	GPL8842	Illumina NovaSeq 6000	34	24	10	[72]
	11	GSE109021	GPL10558	Illumina HiSeq 2000	18	15	3	[73]
Cell lines with Gleason scores and Cells with MYC overexpression	12	GSE200879	GPL32170	Illumina HiSeq 2500	124	115	9	[74]
	13	GSE103512	GPL13158	Illumina NovaSeq 6000	57	50	7	[49]
Cell lines between Age and Race	14	GSE104131	GPL16791	Illumina HiSeq 2500	29	16	13	[75]
	15	GSE200167	GPL24676	Illumina NovaSeq 6000	6	3	3	[76]
Total Samples					364	272	92	

GEO profile description: GSE70466: normal prostate cells vs. androgen-sensitive LNCaP. GSE151290: LNCaP controls vs. LNCaP-R1881. GSE128749: LNCaP-R1881 vs. LAPC4-R1881. GSE120660: PC3-R1881 vs. VCaP-R1881, PC3-R1881 vs. LNCaP-R1881, and LNCaP-R1881 vs. VCaP-R1881. GSE135879: PC3 controls vs. PC3-R1881, LAPC4 controls vs. LAPC4-R1881, VCaP controls vs. VCaP-R1881, and LNCaP controls vs. LNCaP-R1881. GSE136272: VCaP controls vs. VCaP-R1881 and LNCaP controls vs. LNCaP-R1881. GSE218556: controls vs. cells treated with DHT. GSE64529: Control LNCaP cells vs. DHT-treated LNCaP cells. GSE5590: Normal human prostate + zinc and PC3 + zinc. GSE128339: Normal prostate cells vs. control, PC3 adenocarcinoma, and PC3 adenocarcinoma + Genistein. GSE109021: Identification of AR with exposure conditions (Bisphenol A, DHT, Methyl testosterone, Estrogen, Testosterone propionate, DDE, and triclosan) for LAPC-4 cells (examined in antagonist mode) treated with R1881. GSE200879: Control cells vs. PCa Gleason scores. GSE103512: Control cells differing in age of PCa cases. GSE104131: Control cells differing in race/ethnicity of PCa (African American men (AAM) and European American men (EAM)). GSE200167: MYC (TF) in the chromatin interactions of PCa (22Rv1 controls vs. 22Rv1 MYC).

## Data Availability

The datasets generated during the current study are available from the corresponding author upon reasonable request.

## Supplementary Materials

The following supporting information, Figures S1–S13 and Tables S1–S6, can be downloaded at: https://www.mdpi.com/article/10.3390/ijms26125463/s1.

## Abbreviations

ADT	Androgen Deprivation Therapy
AR	Androgen Receptor
BP	Biological Process
cBioPortal	cBio Cancer Genomics Portal
CC	Cellular Component
ChEA	Chip Enrichment Analysis
CT	Computed Tomography
DAVID	Database for Annotation, Visualization, and Integrated Discovery
DEGs	Differentially Expressed Genes
DHT	Dihydrotestosterone
DOIDs	Disease Ontology Identifiers
DNMC	Degree, Density of Maximum Neighborhood Component
E2F4	E2F Transcription Factor 4
EDCs	Endocrine-Disrupting Chemicals
EPC	Edge Percolated Component
FDR	False Discovery Rate
FPKM	Fragments Per Kilobase of transcript per Million
GDC	Genomic Data Commons
GO	Gene Ontology
GEO	Expression Omnibus database
GISTIC	Genomic Identification of Significant Targets in Cancer
GTEx	Genotype-Tissue Expression
ICGC	International Cancer Genome Consortium
KEGG	Kyoto Encyclopedia of Genes and Genomes
KM	Kaplan–Meier
MCC	Maximal Clique Centrality
MCODE	Molecular Complex Detection
MF	Molecular Function
MNC	Maximum Neighborhood Component
MRI	Magnetic Resonance Imaging
MSK	Memorial Sloan Kettering Cancer Center
MYC	MYC Proto-Oncogene, BHLH Transcription Factor
NFY	Nuclear transcription factor Y
NCBI	National Center for Biotechnology Information
NHANES	National Health and Nutrition Examination Survey
PCa	Prostate Cancer
PEI	Pathway Enrichment Analysis
PPI	Protein–Protein Interaction
PSA	Prostate-Specific Antigen
SIGNOR	SIGnaling Network Open Resource
STRING	Retrieval of Interacting Genes/Proteins
TCGA	The Cancer Genome Atlas
TFs	Transcription Factors
TIMER 2.0	Tumor Immunity Evaluation Resource 2.0
TRRUST	Transcriptional Regulatory Relationships Unraveled by Sentence-based Text mining
UCSC_TFBS	University of California, Santa Cruz—transcription factor binding sites
UCSC-Xena	University of California Santa Cruz—Xena
YBX1	Y box binding protein 1
