# Supplementary material for: RNA-Seq Uncovers Association of Endocrine-Disrupting Chemicals with Hub Genes and Transcription Factors in Aggressive Prostate Cancer
Source: Int J Mol Sci. 2025 Jun 6;26(12):5463. doi: 10.3390/ijms26125463 (PMC12192892; doi:10.3390/ijms26125463)
Supplement: Supplementary file 1 [file ijms-26-05463-s001.zip › ijms-3661655-supplementary.pdf]

Appendix A: Supplementary materials

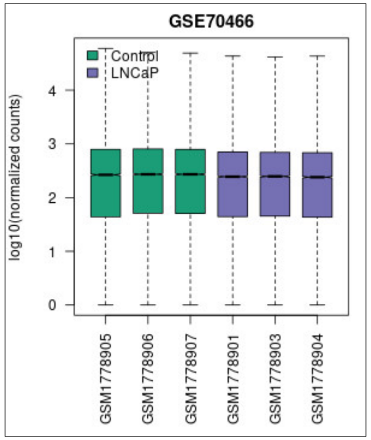

GSE70466: Normal vs. LNCaP

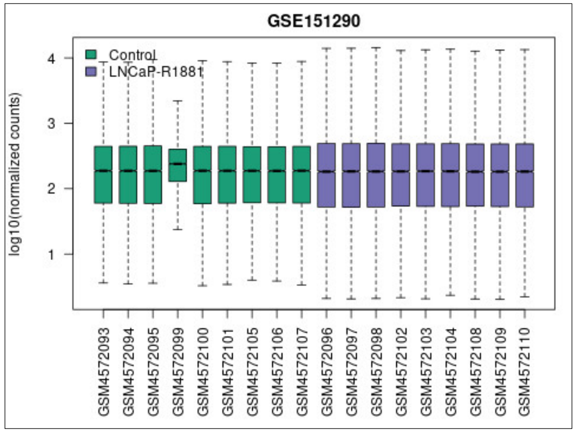

GSE151290: LNCaP vs LNCaP-R1881

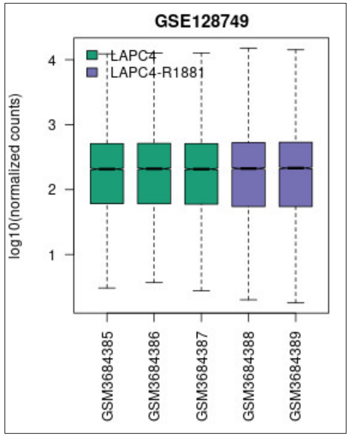

GSE128749:LAPC4 vs.LAPC4+R1881

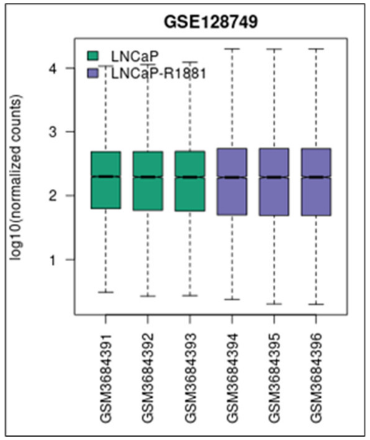

GSE128749: LNCaP vs LNCaP-R1881

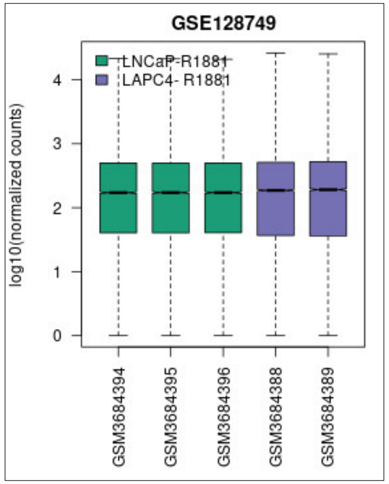

GSE128749:LNCaP-R1881vsLAPC4- R1881

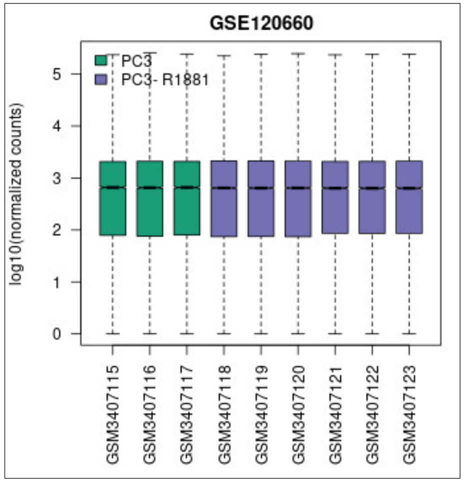

GSE120660: PC3 vs PC3- R1881

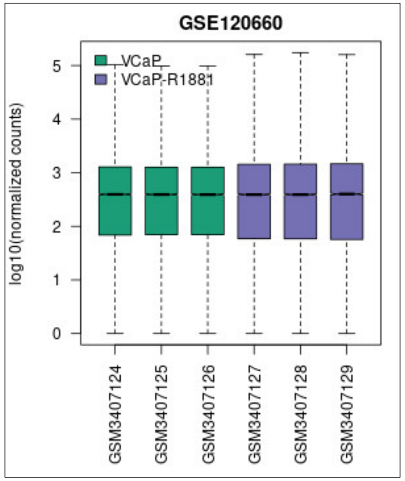

GSE120660: VCaP vs VCaP-R1881

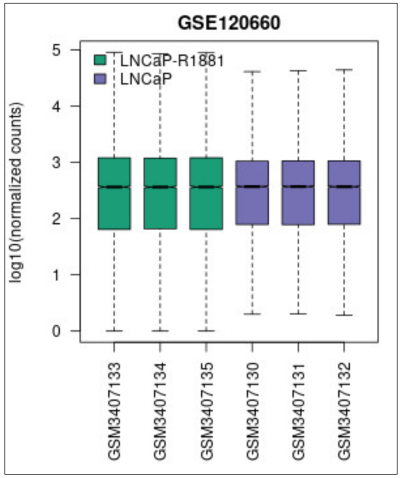

GSE120660: LNCaP vs LNCaP-R1881

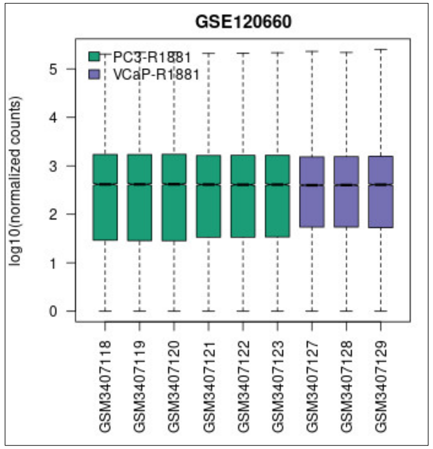

GSE120660: PC3-R1881 vs VCaP-R1881

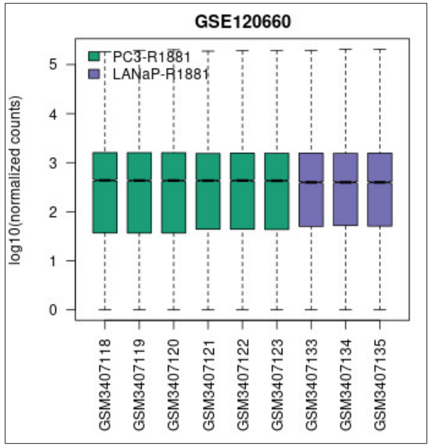

GSE120660: PC3-R1881 vs LNCaP-R1881

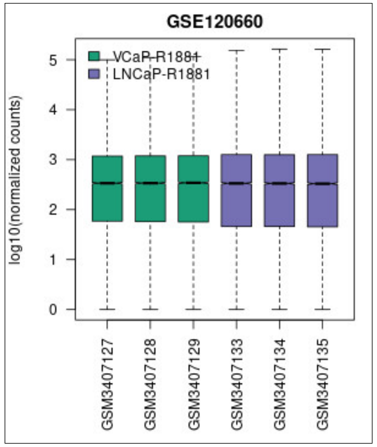

GSE120660: VCaP-R1881 vs LNCaP-R1881

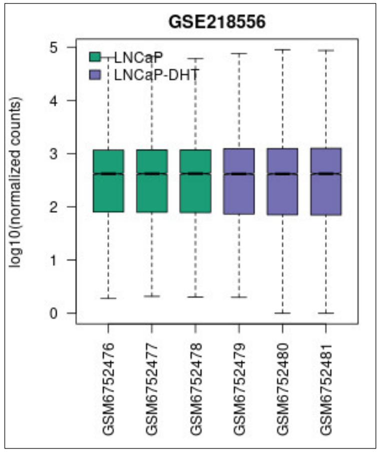

GSE218556: LNCaP vs LNCaP-DHT

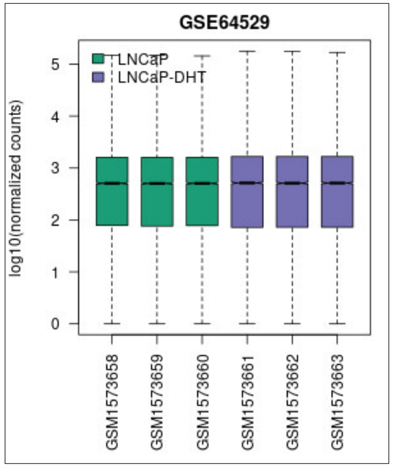

GSE64529: LNCaP vs LNCaP-DHT

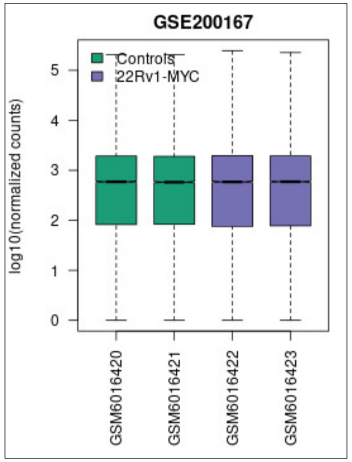

GSE200167: 22Rv1 - Controls vs 22Rv1-MYC

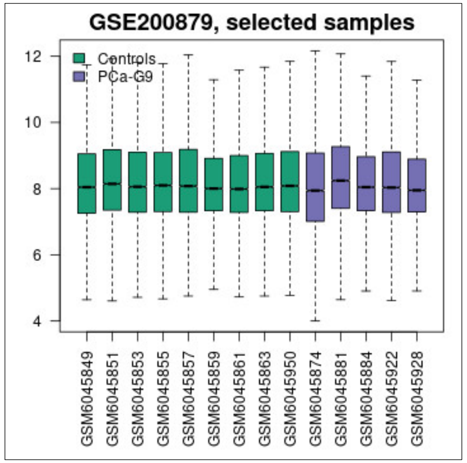

GSE200879: Normal (9) vs PCa-G9

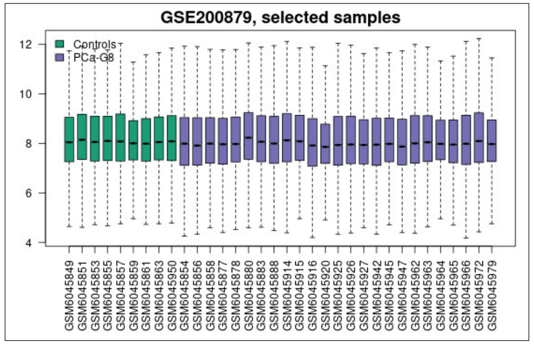

GSE200879: Normal (9) vs PCa-G8

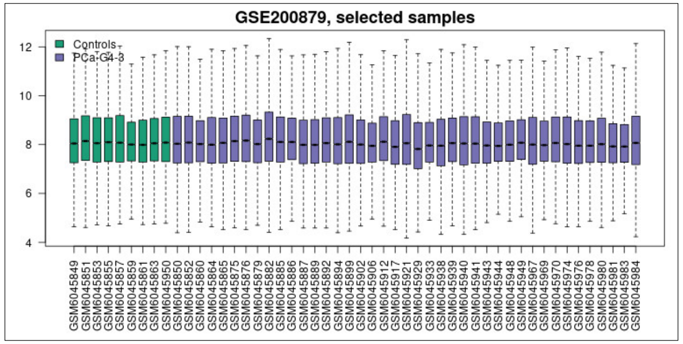

GSE200879: Normal (9) vs PCa-G7(4+3)

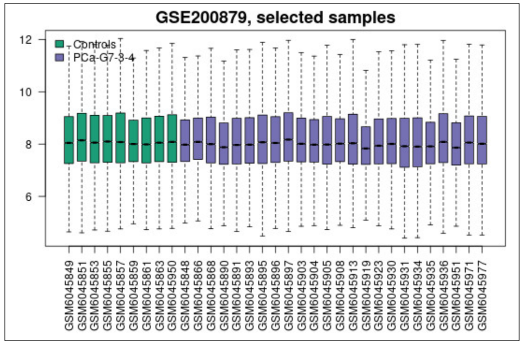

GSE200879: Normal (9) vs PCa-G7(3+4)

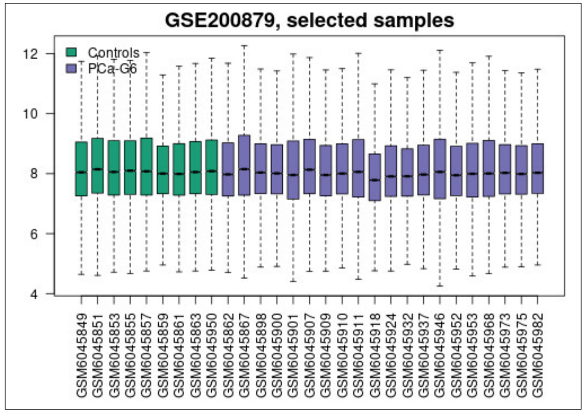

GSE200879: Normal (9) vs PCa-G6

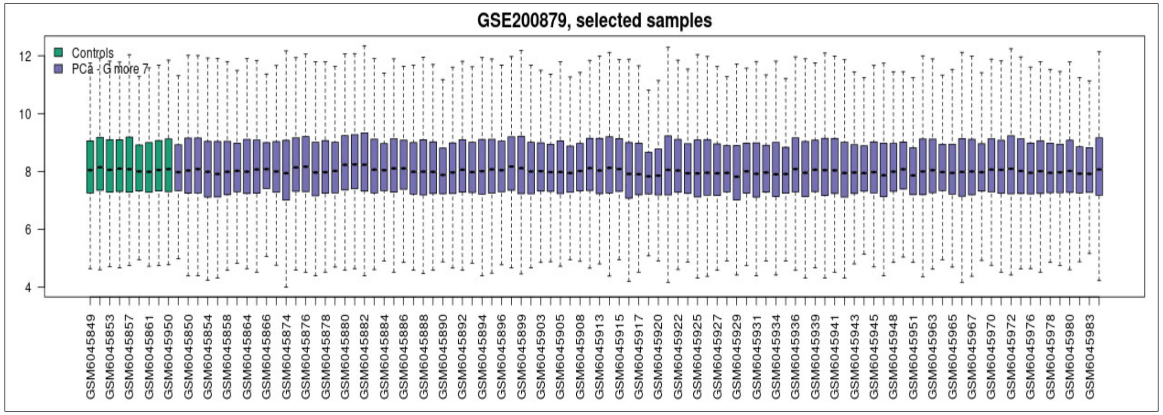

GSE103512: Normal vs PCa (≥70)

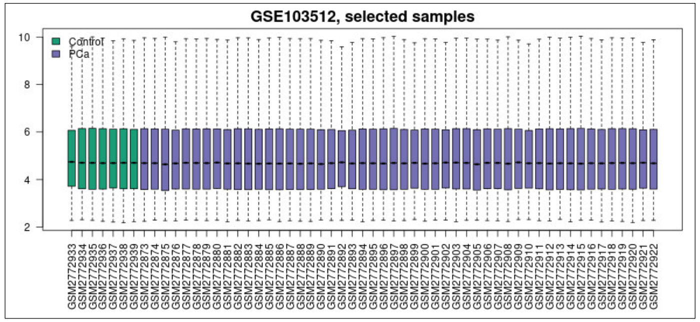

GSE103512: Normal vs PCa

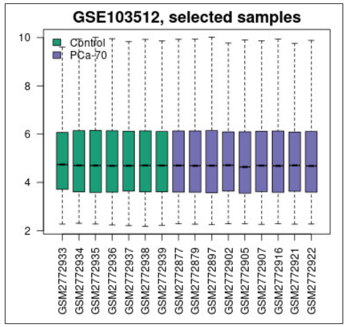

GSE103512: Normal vs PCa (≥70)

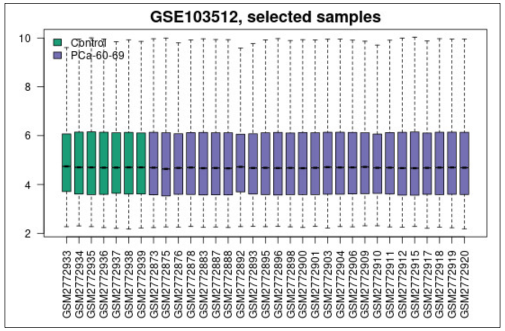

GSE103512: Normal vs PCa (60-69)

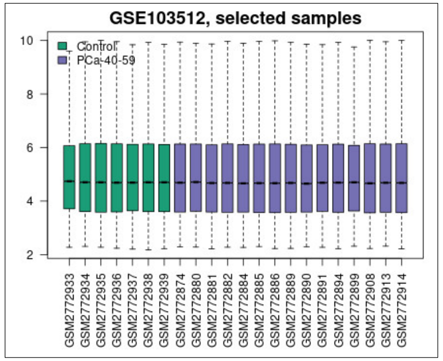

GSE103512: Normal vs PCa (40-59)

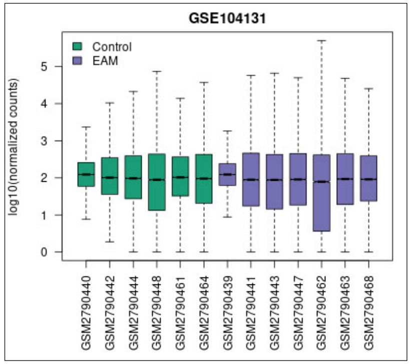

GSE104131: Control vs EAM

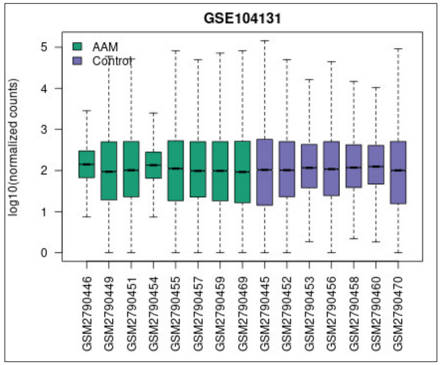

GSE104131: Control vs AAM

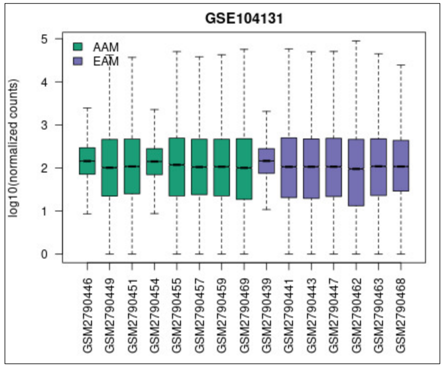

GSE104131: EAM vs AAM

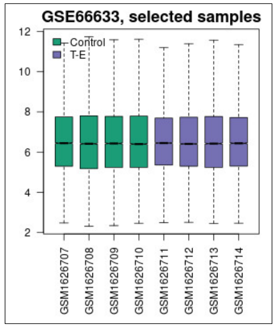

GSE66633: Control vs T&E

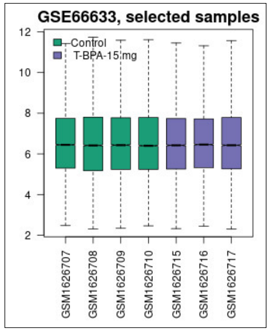

GSE66633: Control vs T&BPA(15 mg)

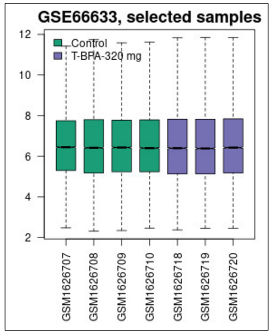

GSE66633: Control vs T& BPA(320 mg)

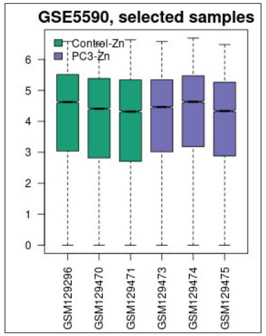

GSE5590: Control-Zn vs PC3-Zn

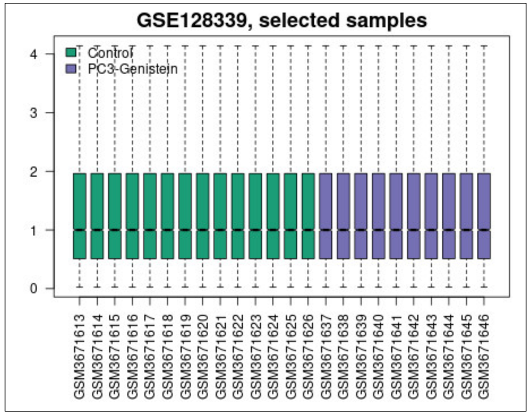

GSE128339: Control vs. PC3+ Genistein

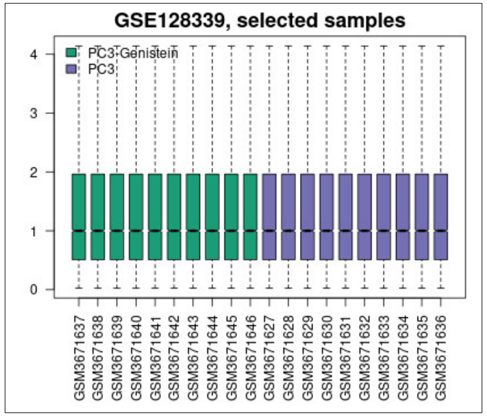

GSE128339: PC3 vs. PC3+ Genistein

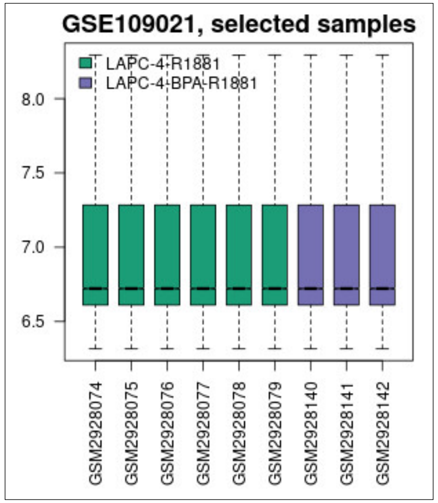

GSE109021: LAPC-4 (R1881) vs. LAPC-4 (BPA +R1881)

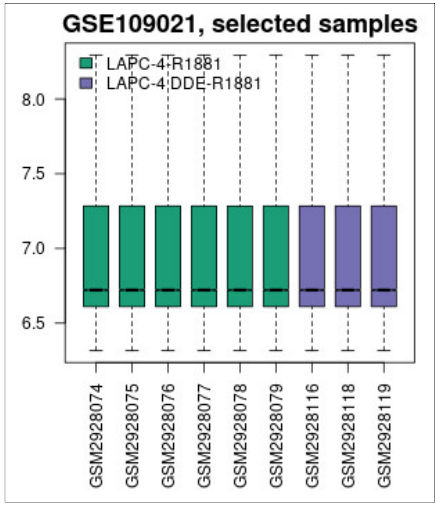

GSE109021: LAPC-4 (R1881) vs. LAPC-40 (DDE +R1881)

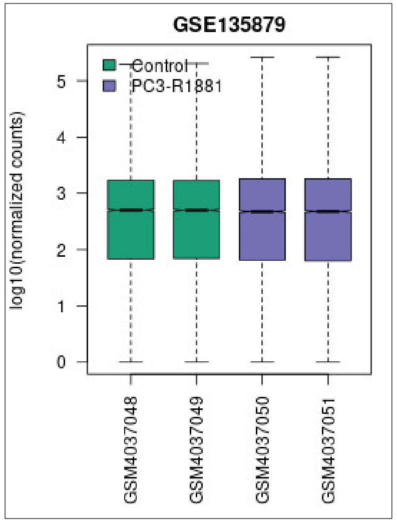

GSE135879- PC3 Controls vs PC3-R1881

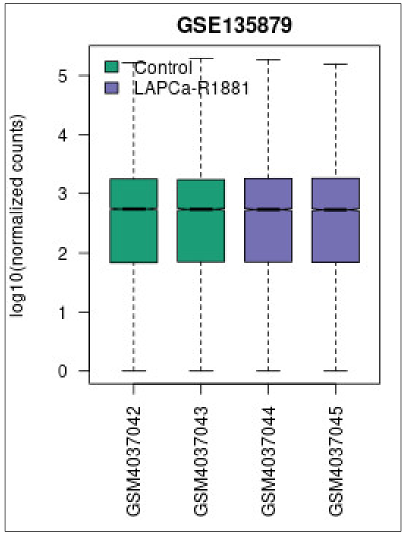

GSE135879 - LAPC4 Controls vs LAPC4-R1881

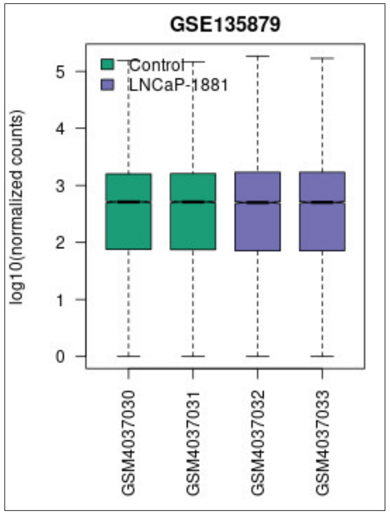

GSE135879 - LNCaP Controls vs LNCaP-R1881

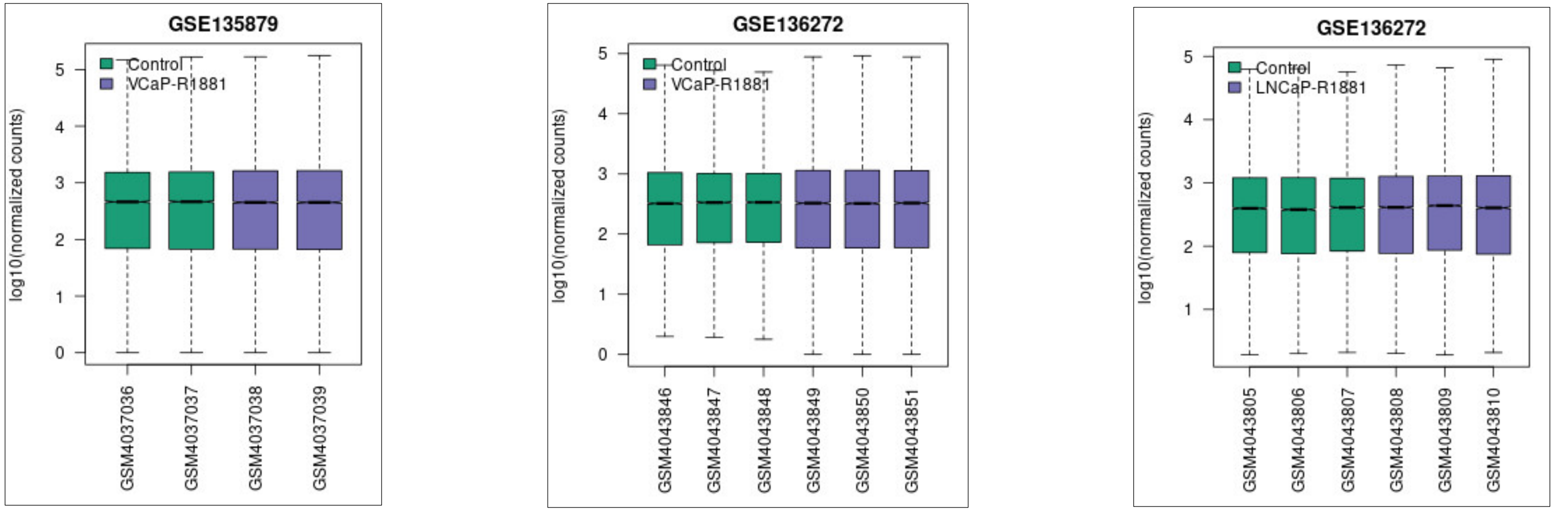

GSE135879 - VCaP Controls vs VCaP-R1881 GSE136272 - VCaP Controls vs VCaP-R1881 GSE136272 - LNCaP Controls vs LNCaP-R1881

Figure S1: Boxplots for data standardization of the values of the selected RNA-seq databases Samples. Green boxplots represent data before normalization, and blue boxplots represent normalized data (Quantitative normalization).

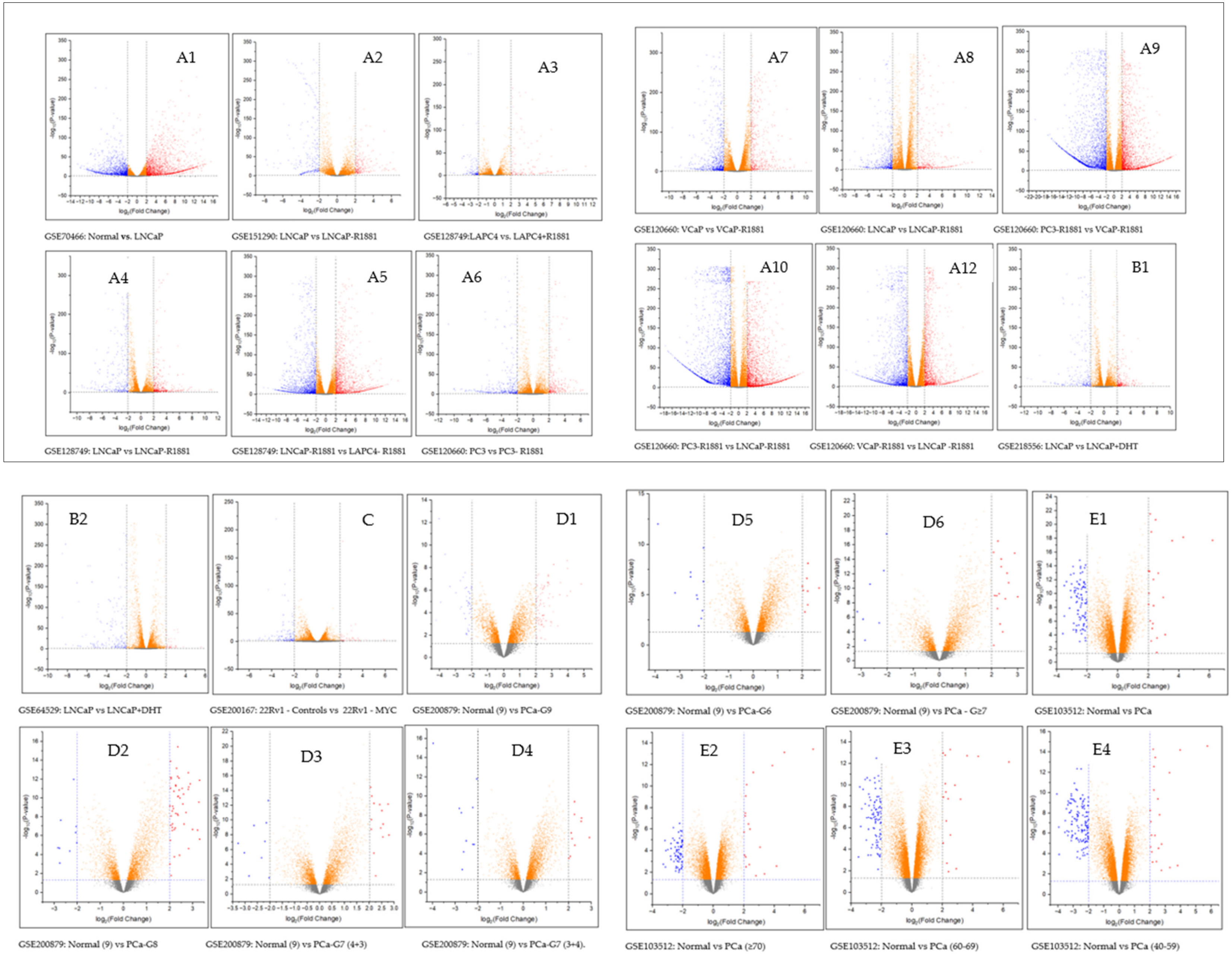
A1
GSE70466: Normal vs. LNCaP
A2
GSE151290: LNCaP vs LNCaP-R1881
A3
GSE128749:LAPC4 vs. LAPC4+R1881
A7
GSE120660: VCaP vs VCaP-R1881
A8
GSE120660: LNCaP vs LNCaP-R1881
A9
GSE120660: PC3-R1881 vs VCaP-R1881
A4
GSE128749: LNCaP vs LNCaP-R1881
A5
GSE128749: LNCaP-R1881 vs LAPC4- R1881
A6
GSE120660: PC3 vs PC3- R1881
A10
GSE120660: PC3-R1881 vs LNCaP-R1881
A12
GSE120660: VCaP-R1881 vs LNCaP -R1881
B1
GSE218556: LNCaP vs LNCaP+DHT
B2
GSE64529: LNCaP vs LNCaP+DHT
C
GSE200167: 22Rv1 - Controls vs 22Rv1 - MYC
D1
GSE200879: Normal (9) vs PCa-G9
D5
GSE200879: Normal (9) vs PCa-G6
D6
GSE200879: Normal (9) vs PCa - G≥7
E1
GSE103512: Normal vs PCa
D2
GSE200879: Normal (9) vs PCa-G8
D3
GSE200879: Normal (9) vs PCa-G7 (4+3)
D4
GSE200879: Normal (9) vs PCa-G7 (3+4).
E2
GSE103512: Normal vs PCa (≥70)
E3
GSE103512: Normal vs PCa (60-69)
E4
GSE103512: Normal vs PCa (40-59)

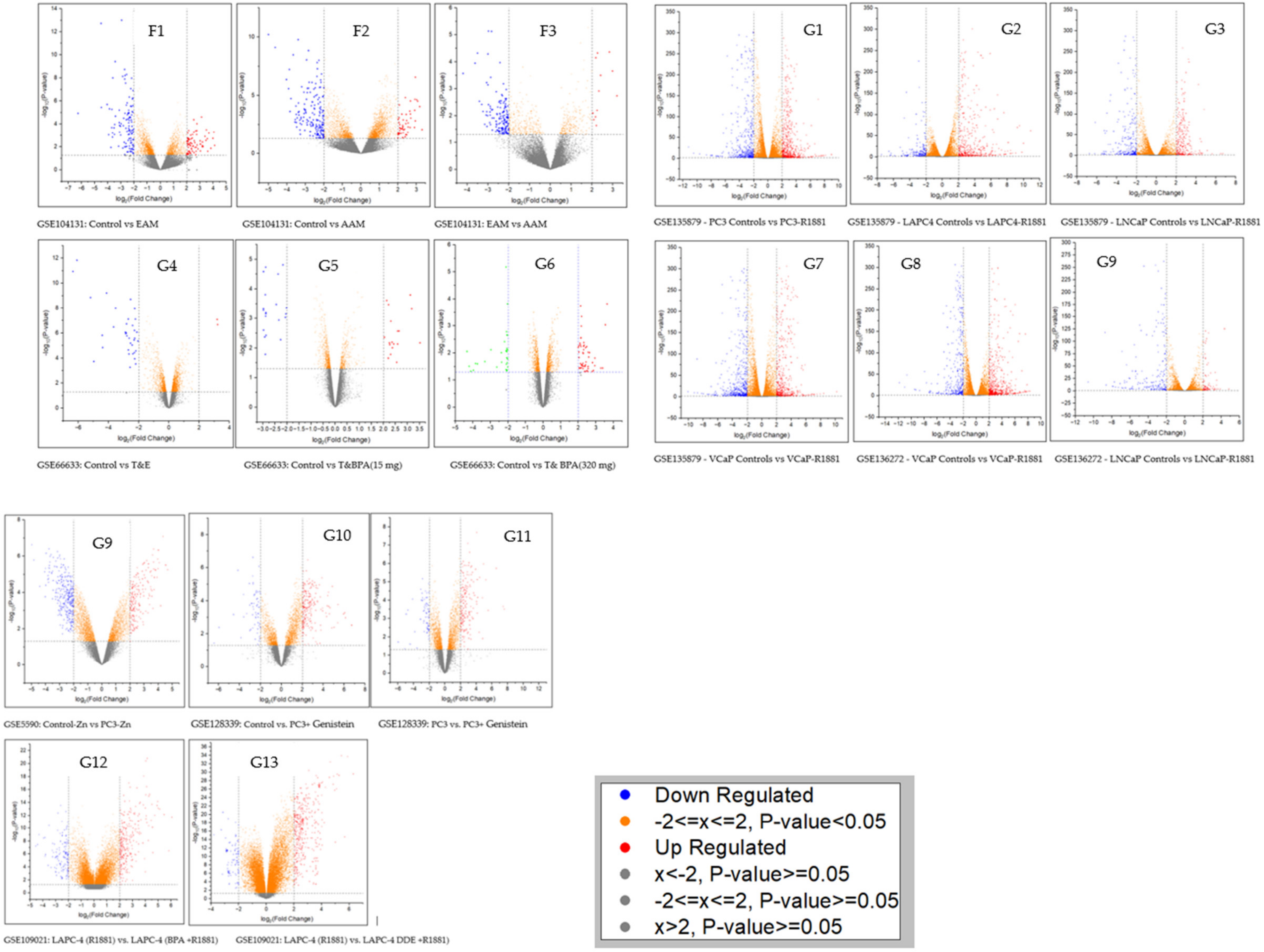

Figure S2: Analysis of Differentially Expressed Genes (DEGs) Using Volcano Plots in RNA-Seq Databases. This figure presents volcano plots (A-G) illustrating the identification of DEGs. Upregulated genes are depicted in red, downregulated genes in blue, and genes without significant expression changes are in orange. The selection criteria of the cutoff standards of adjusted p-value < 0.05 and [log2FC] > +2, [log2FC] < -2 from OriginPro 9.1.

Table S1: Enrichment analysis revealed the top five Gene Ontology (GO) terms and KEGG pathways associated with the 38 upregulated genes in Biological Processes (BP), Cellular Components (CC), Molecular Functions (MF), and KEGG pathways for prostate cancer (PCa).

	BP	Term	Count	P-Value	Genes
1	GO:0051301	Cell division	10	3.37×10^{-8}	CCNA2, CCNA1, HELLS, CCNB1, KIF18B, CKS2, NCAPG, CDCA8, NDC80, CKS1B.
2	GO:0044772	Mitotic cell cycle phase transition	5	1.17×10^{-7}	CCNA2, CCNA1, CCNB1, CKS2, CKS1B
3	GO:0006281	DNA repair	6	1.79×10^{-5}	BARD1, POLQ, BRIP1, EXO1, RAD54L, BRCA1, BRCA2
4	GO:0000070	Mitotic sister chromatid segregation	4	4.40×10^{-5}	KIF18B, ESPL1, CDCA8, NDC80
5	GO:0071479	Cellular response to ionizing radiation	6	6.05×10^{-5}	BARD1, CDKN1A, BRCA1, BRCA2
	CC	Term	Count	P-Value	
1	GO:0000307	Cyclin-dependent protein kinase holoenzyme complex	6	1.30×10^{-8}	CCNA2, CCNA1, CDKN1A, CCNB1, CKS2, CKS1B
2	GO:0005634	Nucleus	28	4.25×10^{-8}	CDKN1A, MAX, NCAPG, MCM10, BRCA1, MKI67, BRCA2, BRIP1, CCNB1, ORC1, EXO1, RAD54L, E2F7, BARD1, PLK4, CDK18, HELLS, PTCH1, NDC80, GADD45G, CCNA2, CCNA1, NEIL3, KIF18B, ESPL1, BIN1, KRT15, DTL
3	GO:0005654	Nucleoplasm	23	7.79×10^{-8}	BARD1, POLQ, CDKN1A, MAX, CDCA8, MCM10, BRCA1, MKI67, BRCA2, NDC80, CKS1B, CCNA2, CCNA1, NEIL3, BRIP1, CCNB1, KIF18B, ORC1, EXO1, RAD54L, GTSE1, DTL, E2F7
4	GO:0070532	BRCA1-B complex	3	1.88×10^{-5}	BARD1, BRIP1, BRCA1
5	GO:0000152	Nuclear ubiquitin ligase complex	3	4.69×10^{-5}	BARD1, BRCA1, BRCA2
	MF	Term	Count	P-Value	
1	GO:0003682	Cyclin-dependent protein serine/threonine kinase regulator activity	4	2.98×10^{-5}	CCNA2, CCNA1, CCNB1, CKS1B
2	GO:0005515	Protein binding	36	5.18×10^{-5}	CDKN1A, MAX, NCAPG, CDCA8, MCM10, BRCA1, MKI67, BRCA2, CKS1B, BRIP1, CCNB1, ORC1, EXO1, RAD54L, GTSE1, E2F7, BARD1, PLK4, CDK18, POLQ, HELLS, PTCH1, FN1, NDC80, TGFBR2, GADD45G, CCNA2, CCNA1, KRT19, KIF18B, MELK, ESPL1, BIN1, KRT15, CKS2, DTL
3	GO:0005524	ATP binding	12	1.17×10^{-4}	ACTA2, PLK4, POLQ, CDK18, HELLS, BRIP1, MELK, KIF18B, ORC1, RAD54L, MKI67, TGFBR2
4	GO:0061575	cyclin-dependent protein serine/threonine kinase activator activity	3	6.9×10^{-4}	CCNB1, CKS2, CKS1B
5	GO:0003682	Chromatin binding	6	2.04×10^{-3}	POLQ, HELLS, BRIP1, ORC1, EXO1, CKS2
	KEGG	Term	Count	P-Value	
1	hsa05200	Pathways in cancer	11	1.60×10^{-6}	CCNA2, CCNA1, CDKN1A, MAX, PTCH1, CKS2, FN1, BRCA2, GADD45G, TGFBR2, CKS1B
2	hsa05215	Prostate cancer	7	1.70×10^{-6}	CCNA2, CCNA1, CDKN1A, CCNB1, ESPL1, ORC1, GADD45G
3	hsa04110	Cell cycle	5	6.29×10^{-6}	BARD1, BRIP1, RAD54L, BRCA1, BRCA2
4	hsa04115	p53 signaling pathway	4	1.35×10^{-5}	CDKN1A, CCNB1, GTSE1, GADD45G
5	hsa04068	FoxO signaling pathway	5	2.48×10^{-4}	PLK4, CDKN1A, CCNB1, GADD45G, TGFBR2

Table S2: Enrichment analysis revealed the top five Gene Ontology (GO) terms and KEGG pathways associated with the 37 downregulated genes in Biological Processes (BP), Cellular Components (CC), Molecular Functions (MF), and KEGG pathways for prostate cancer (PCa).

	BP	Term	Count	P-Value	Genes
1	GO:0051301	Cell division	13	2.63×10^{-12}	UBE2C, BUB1B, KIF11, ZWINT, AURKA, CDC20, ASPM, CENPE, CCNB2, CENPF, CDK1, BUB1, SPC25
2	GO:0007094	Mitotic spindle assembly checkpoint	7	2.34×10^{-11}	CDC20, CENPF, BUB1B, TTK, BUB1, ZWINT, SPC25
3	GO:0007052	mitotic spindle organization	6	6.51×10^{-8}	CENPE, TTK, KIF11, DLGAP5, AURKA, SPC25
4	GO:0007059	Chromosome segregation	6	3.19×10^{-7}	TOP2A, CENPE, CENPF, TTK, DLGAP5, SPC25
5	GO:0007051	Spindle organization	4	4.73×10^{-6}	ASPM, TTK, KIF11, AURKA
	CC	Term	Count	P-Value	
1	GO:0005819	Spindle	9	1.43×10^{-10}	CDC20, ASPM, CENPF, BUB1B, TTK, KIF11, HMMR, KIF15, AURKA
2	GO:0000776	Kinetochore	7	2.67×10^{-7}	CENPE, CENPF, BUB1B, TTK, BUB1, ZWINT, SPC25
3	GO:0005813	Centrosome	10	3.63×10^{-7}	CDC20, ASPM, CCNB2, CENPF, DHX9, CDK1, HMMR, HDAC6, KIF15, AURKA
4	GO:0005829	Cytosol	22	6.64×10^{-5}	RRM2, UBE2C, DHX9, BUB1B, KIF11, HMMR, PKMYT1, ZWINT, HDAC6, KIF15, AURKA, CDC20, CENPE, CCNB2, CENPF, INPP5E, CDK1, IL7R, BUB1, DLGAP5, CDKN3, SPC25
5	GO:0030496	Midbody	5	2.97×10^{-4}	ASPM, CENPE, CENPF, CDK1, AURKA
	MF	Term	Count	P-Value	
1	GO:0005524	ATP binding	12	8.90×10^{-5}	TOP2A, CENPE, UBE2C, DHX9, CDK1, BUB1B, TTK, KIF11, PKMYT1, BUB1, KIF15, AURKA
2	GO:0008017	Microtubule binding	6	130×10^{-4}	CENPE, CENPF, KIF11, DLGAP5, HDAC6, KIF15
3	GO:0005515	Protein binding	36	5.00×10^{-4}	TOP2A, DHX9, BUB1B, TTK, HMMR, KIF11, PKMYT1, HDAC6, AURKA, KIF15, ICAM1, CDC20, CCNB2, MYC, CCL5, LYAR, IL6R, BUB1, DLGAP5, TGFB2, RRM2, VWF, UBE2C, IGF1, F3, ZWINT, CENPE, CXCL10, CENPF, LOX, CDK1, IL7R, CDKN3, SPC25
4	GO:0004672	Protein kinase activity	6	7.03×10^{-4}	CCL5, CDK1, BUB1B, PKMYT1, BUB1, AURKA
5	GO:0004674	Protein serine/threonine kinase activity	6	8.77×10^{-4}	CDK1, BUB1B, TTK, PKMYT1, BUB1, AURKA
	KEGG	Term	Count	P-Value	
1	hsa05200	Pathways in cancer	9	1.58×10^{-9}	CDC20, CCNB2, TGFB2, MYC, CDK1, BUB1B, TTK, PKMYT1, BUB1
2	hsa05215	Prostate cancer	7	1.57×10^{-6}	CDC20, CCNB2, CDK1, IGF1, PKMYT1, BUB1, AURKA
3	hsa04110	Cell cycle	6	9.48×10^{-6}	CCNB2, CDK1, IGF1, PKMYT1, BUB1, AURKA
4	hsa04115	p53 signaling pathway	6	3.87×10^{-4}	CDC20, CCNB2, TGFB2, MYC, BUB1B, ICAM1
5	hsa04068	FoxO signaling pathway	4	1.98×10^{-3}	CCNB2, RRM2, CDK1, IGF1

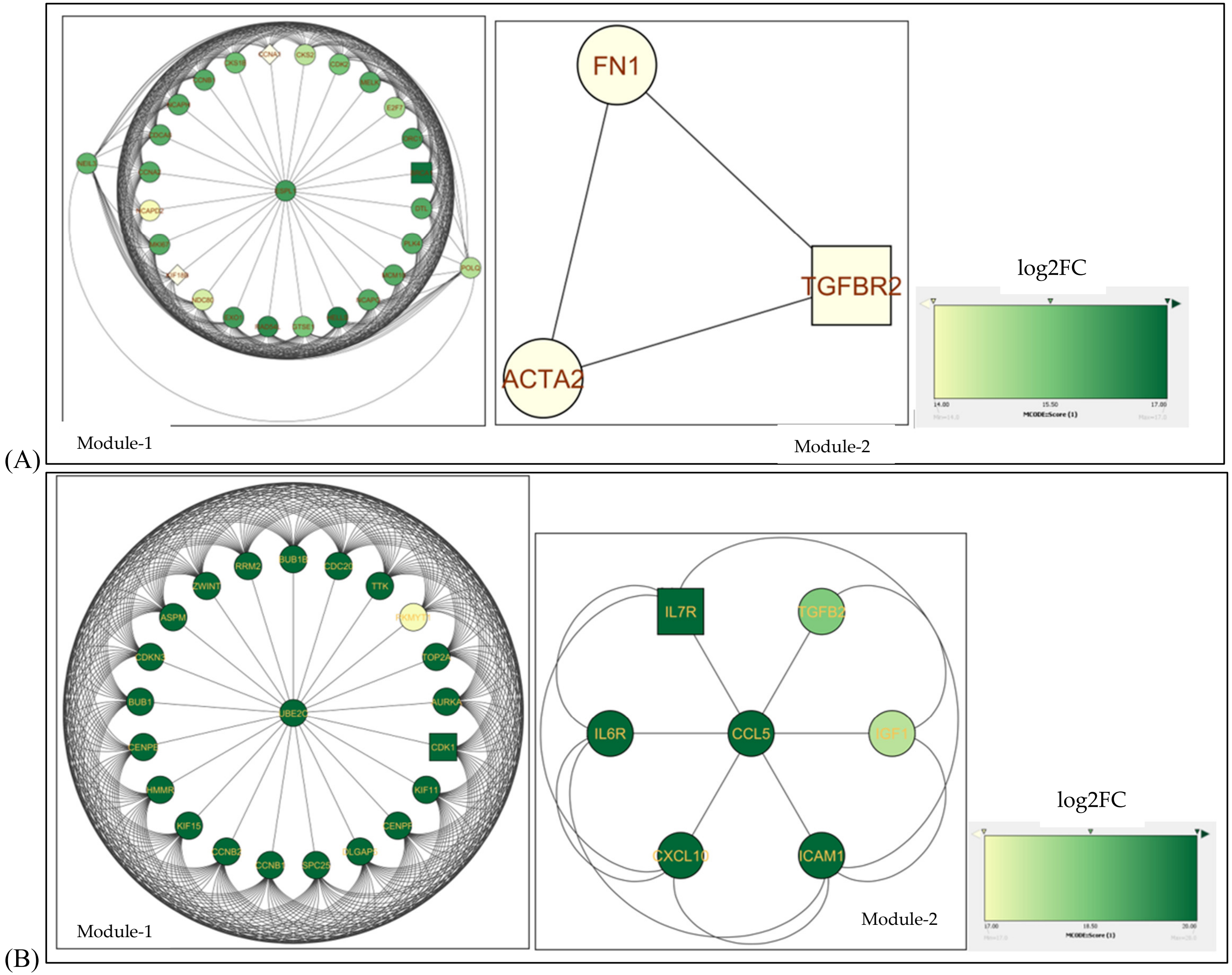

Figure S3. The four modules were developed from the PPI network from RNA-seq DEGs by MCODE. (A) Module-1: upregulated genes were associated with a score of 16.3 and contained 25 nodes/genes and 612 edges, and module-2 was associated with a score of 2.4, including three nodes/genes with three edges. (B): In downregulated, Module-1 was connected with a score of 17.6 and contained 23 nodes/genes and 413 edges, and module-2 was associated with a score of 4.0, including seven nodes/genes

with 18 edges. Seed genes are highlighted in the square shape. The clustered analysis based on the log2FC represents the color code: light green and dark green represent low and high expression, respectively.

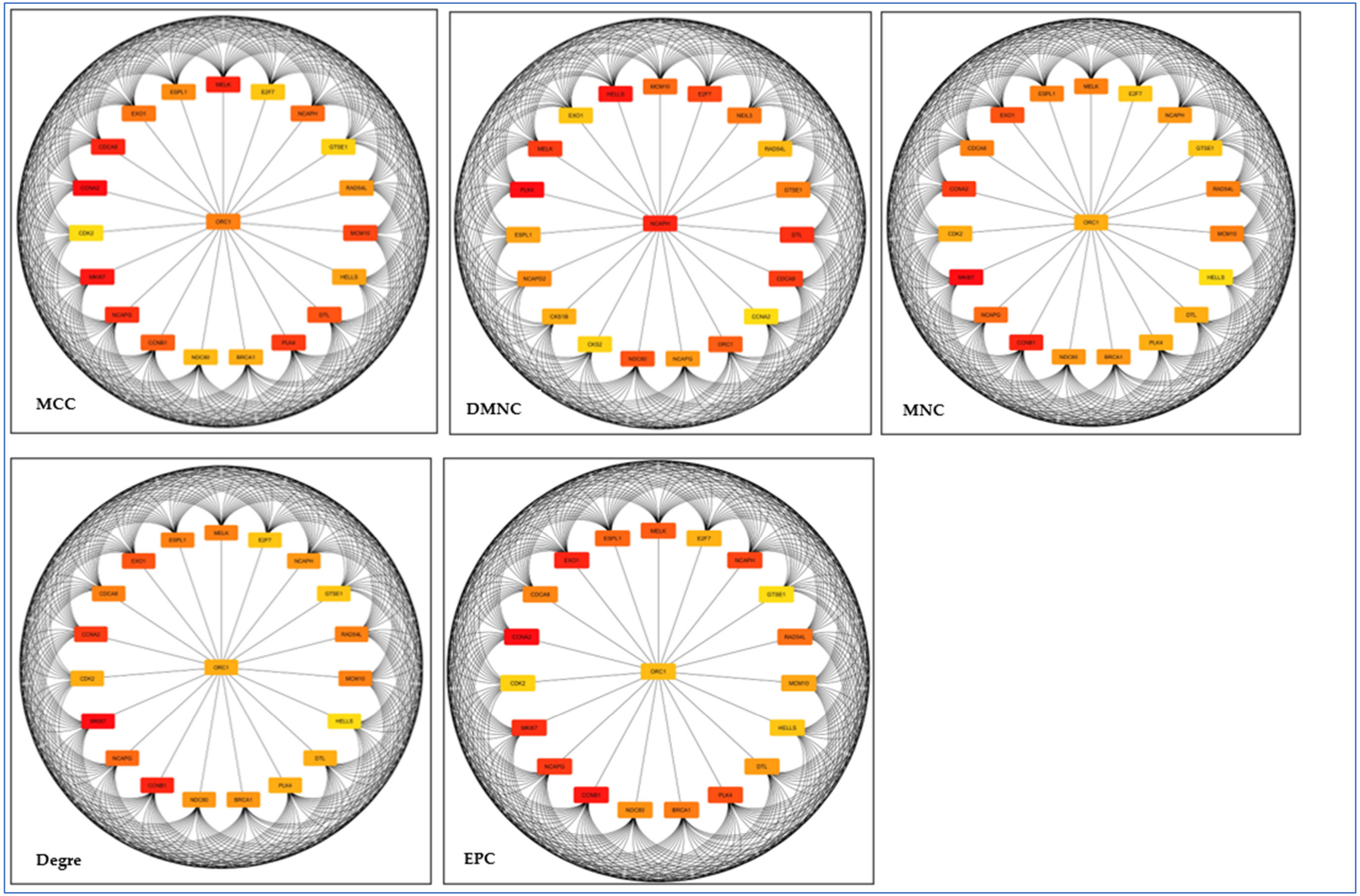

(A)

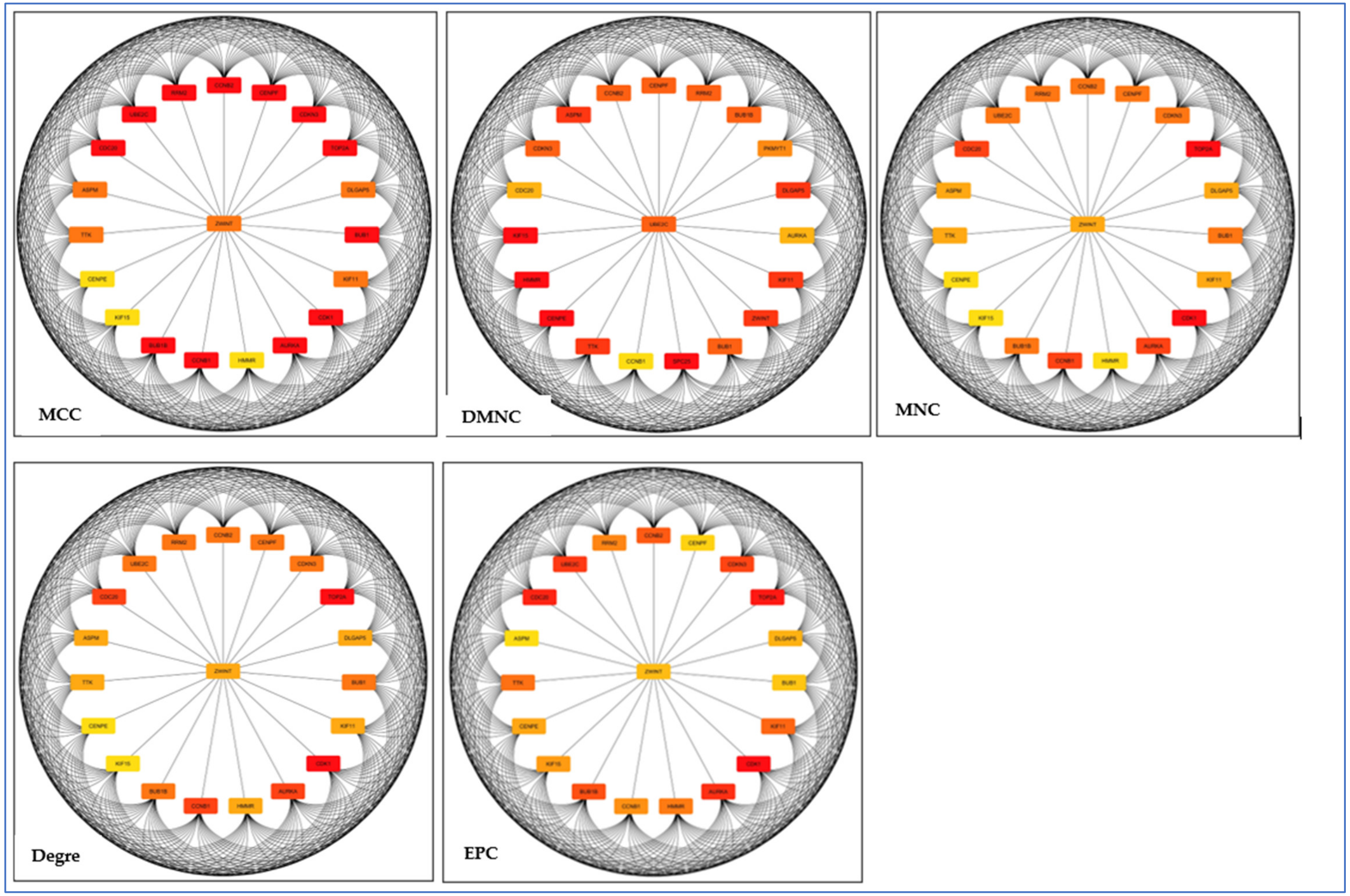

(B)

Figure S4. A summary of the gene networks from the PPI network that are regulated and expressed in PCa samples was evaluated utilizing CytoHubba. The network was categorized based on clustering coefficients, which generated hierarchically high confidence interactions. These hub genes are revealed with the following clustering coefficients: Red: the highest, Orange: medium, and Yellow: low clustering coefficients are shown in five networks. Panel A depicts upregulated, and Panel B depicts downregulated Hub genes, which were identified based on Cytohubba topological measures (MCC ∩ DMNC ∩ MNC ∩ Degree ∩ EPC).

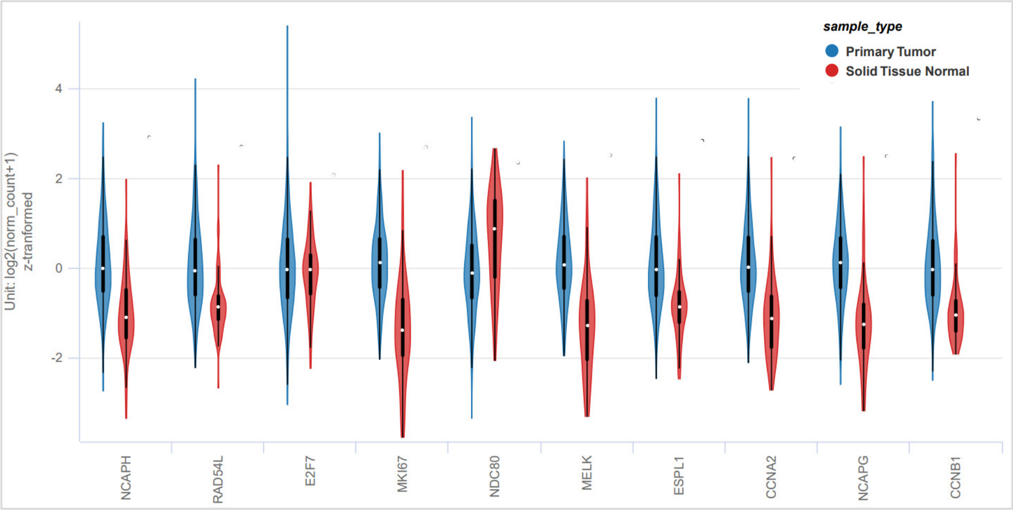

(A)

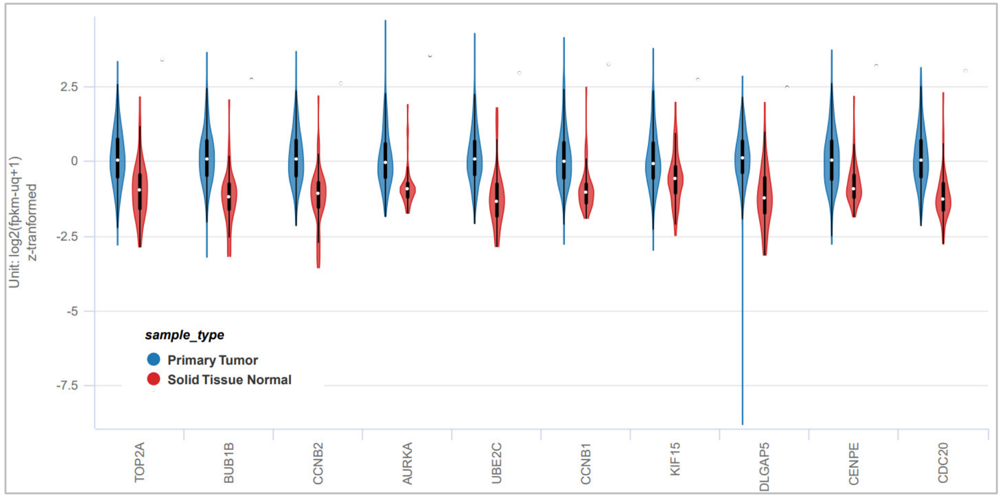

(B)

Figure S5: Differential Expression Analysis of 20 Hub Genes in PCa and Normal Prostate Tissues. Violin plots in this figure compare the expression of 20 hub genes between prostate cancer and normal tissues, utilizing data from TCGA, ICGC, and GTEx via UCSC-Xena. The plots display log2-transformed data (fpkm-uq+1), with gene expression assessed via RNA-Seq - RSEM. The analysis includes a z-transformed y-axis (log2(norm_count+1)), and a p-value threshold of <0.05. Panels A and B represent upregulated and downregulated genes, respectively.

(A)

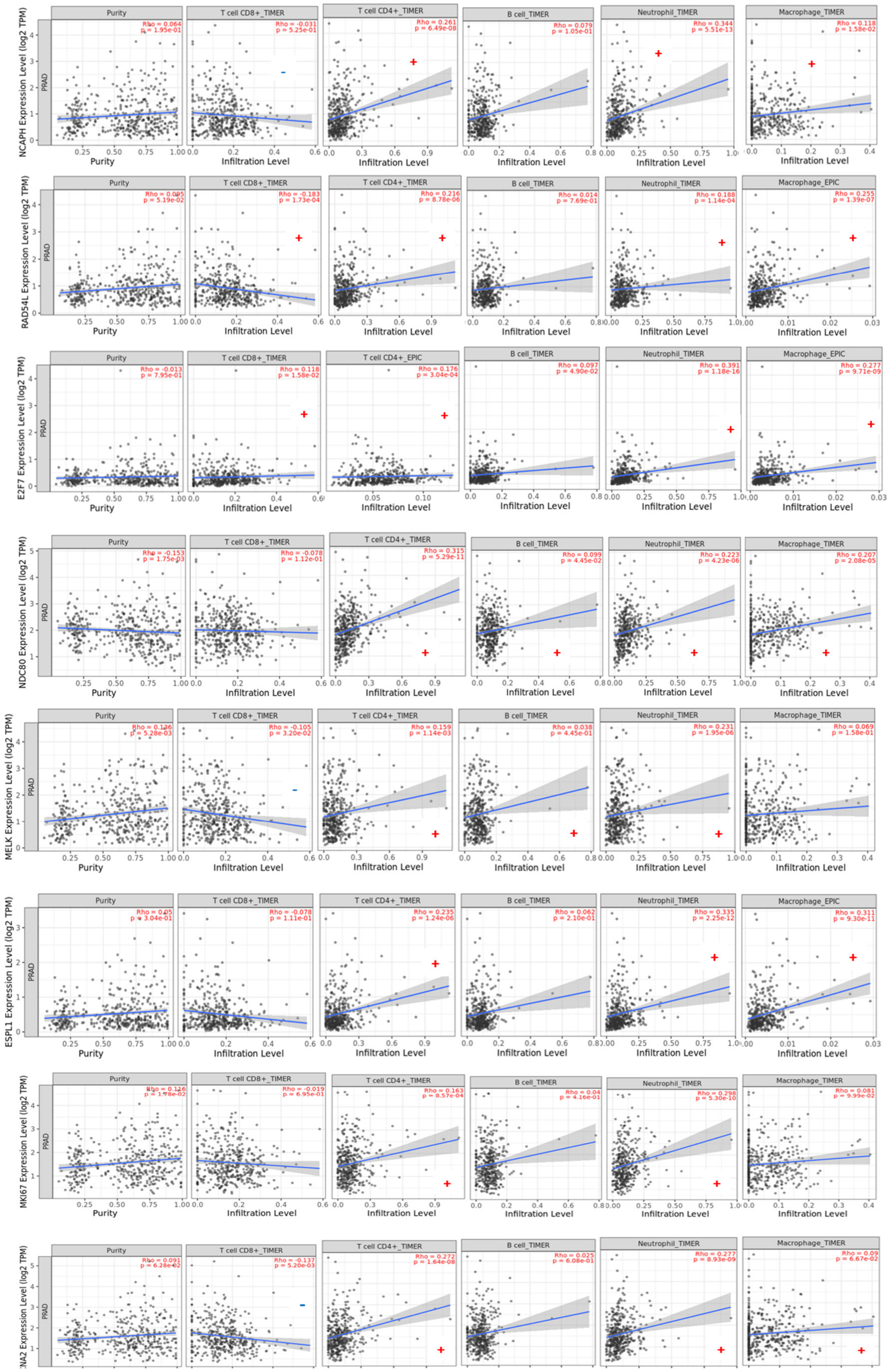

Purity
T cell CD8+_TIMER
T cell CD4+_TIMER
T cell CD4+_EPIC
B cell_TIMER
Neutrophil_TIMER
Macrophage_TIMER
Macrophage_EPIC
Infiltration Level
PRAD
NCAPH Expression Level (log2 TPM)
RAD54L Expression Level (log2 TPM)
E2F7 Expression Level (log2 TPM)
NDC80 Expression Level (log2 TPM)
MELK Expression Level (log2 TPM)
ESPL1 Expression Level (log2 TPM)
MKI67 Expression Level (log2 TPM)

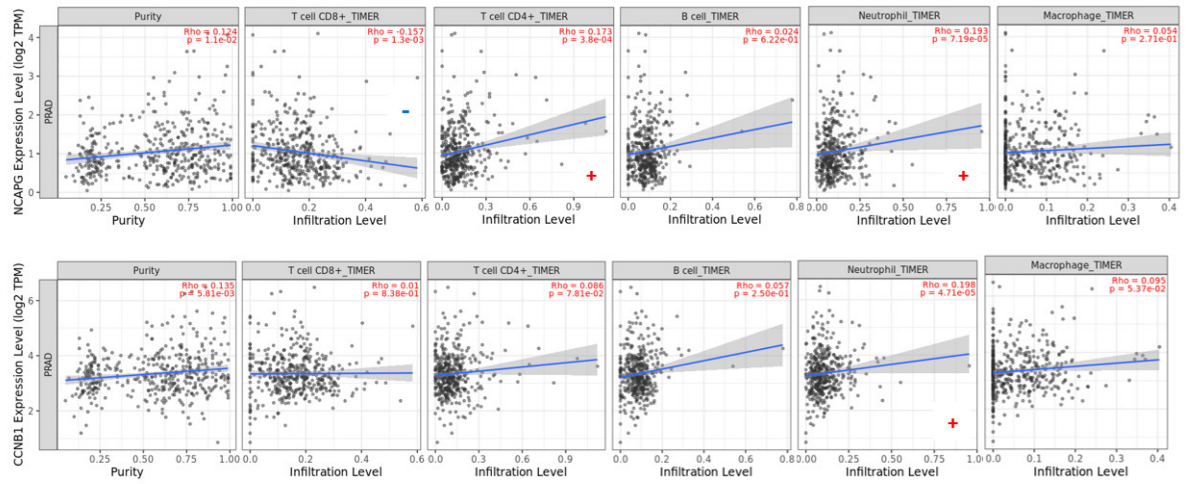
NCAPG Expression Level (log2 TPM)
CCNB1 Expression Level (log2 TPM)
PRAD
Purity
T cell CD8+_TIMER
T cell CD4+_TIMER
B cell_TIMER
Neutrophil_TIMER
Macrophage_TIMER
Rho = 0.124 p = 1.1e-02
Rho = -0.157 p = 1.3e-03
Rho = 0.173 p = 3.8e-04
Rho = 0.024 p = 6.22e-01
Rho = 0.193 p = 7.19e-05
Rho = 0.054 p = 2.71e-01
Rho = 0.135 p = 5.81e-03
Rho = 0.01 p = 8.38e-01
Rho = 0.086 p = 7.81e-02
Rho = 0.057 p = 2.50e-01
Rho = 0.198 p = 4.71e-05
Rho = 0.095 p = 5.37e-02
Purity
Infiltration Level

(B)

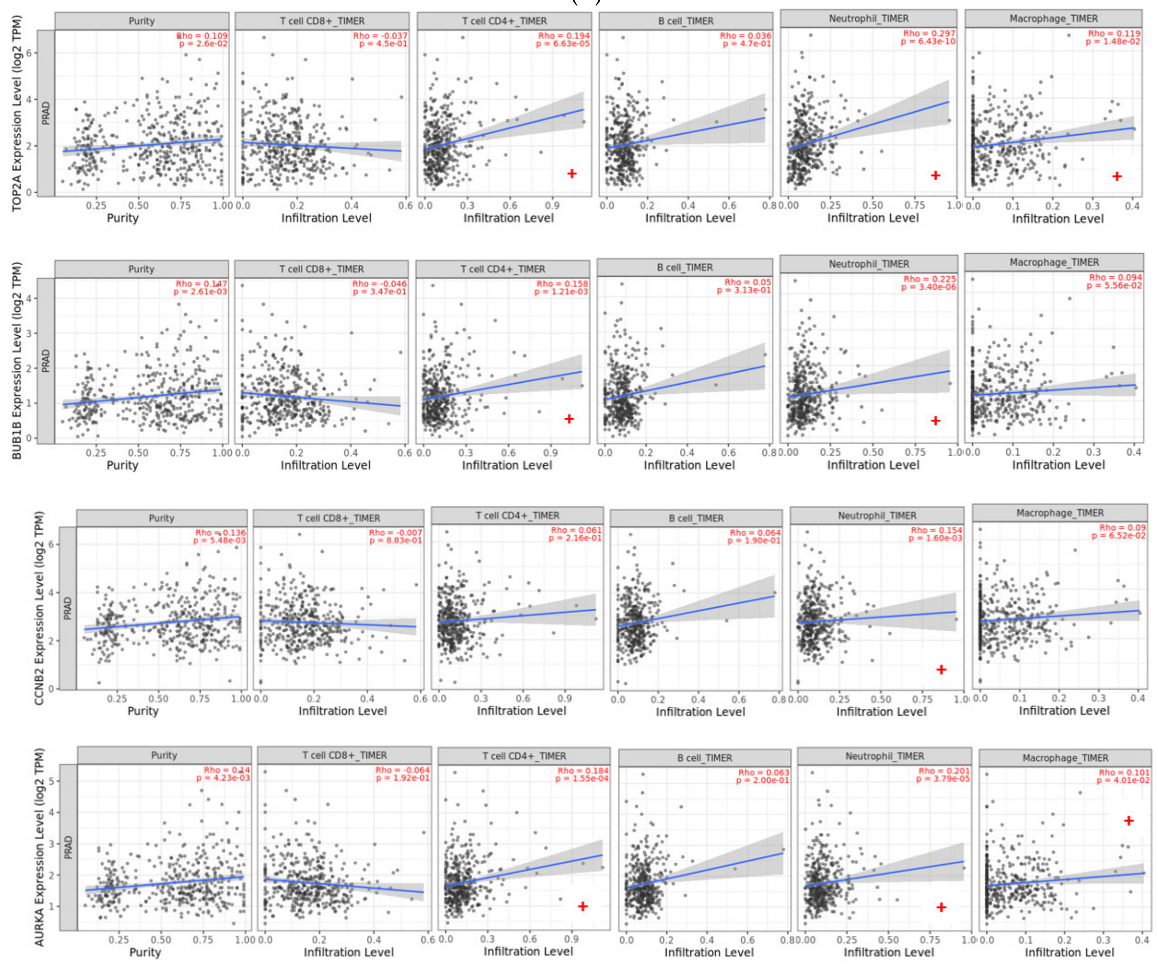
TOP2A Expression Level (log2 TPM)
BUB1B Expression Level (log2 TPM)
CCNB2 Expression Level (log2 TPM)
AURKA Expression Level (log2 TPM)
PRAD
Purity
T cell CD8+_TIMER
T cell CD4+_TIMER
B cell_TIMER
Neutrophil_TIMER
Macrophage_TIMER
Rho = 0.109 p = 2.6e-02
Rho = -0.037 p = 4.5e-01
Rho = 0.194 p = 6.63e-05
Rho = 0.036 p = 4.7e-01
Rho = 0.297 p = 6.43e-10
Rho = 0.119 p = 1.48e-02
Rho = 0.147 p = 2.61e-03
Rho = -0.046 p = 3.47e-01
Rho = 0.158 p = 1.21e-03
Rho = 0.05 p = 3.13e-01
Rho = 0.225 p = 3.40e-06
Rho = 0.094 p = 5.56e-02
Rho = 0.136 p = 5.48e-03
Rho = -0.007 p = 8.83e-01
Rho = 0.061 p = 2.16e-01
Rho = 0.064 p = 1.90e-01
Rho = 0.154 p = 1.60e-03
Rho = 0.09 p = 6.52e-02
Rho = 0.14 p = 4.23e-03
Rho = -0.064 p = 1.92e-01
Rho = 0.184 p = 1.55e-04
Rho = 0.063 p = 2.00e-01
Rho = 0.201 p = 3.79e-05
Rho = 0.101 p = 4.01e-02
Purity
Infiltration Level

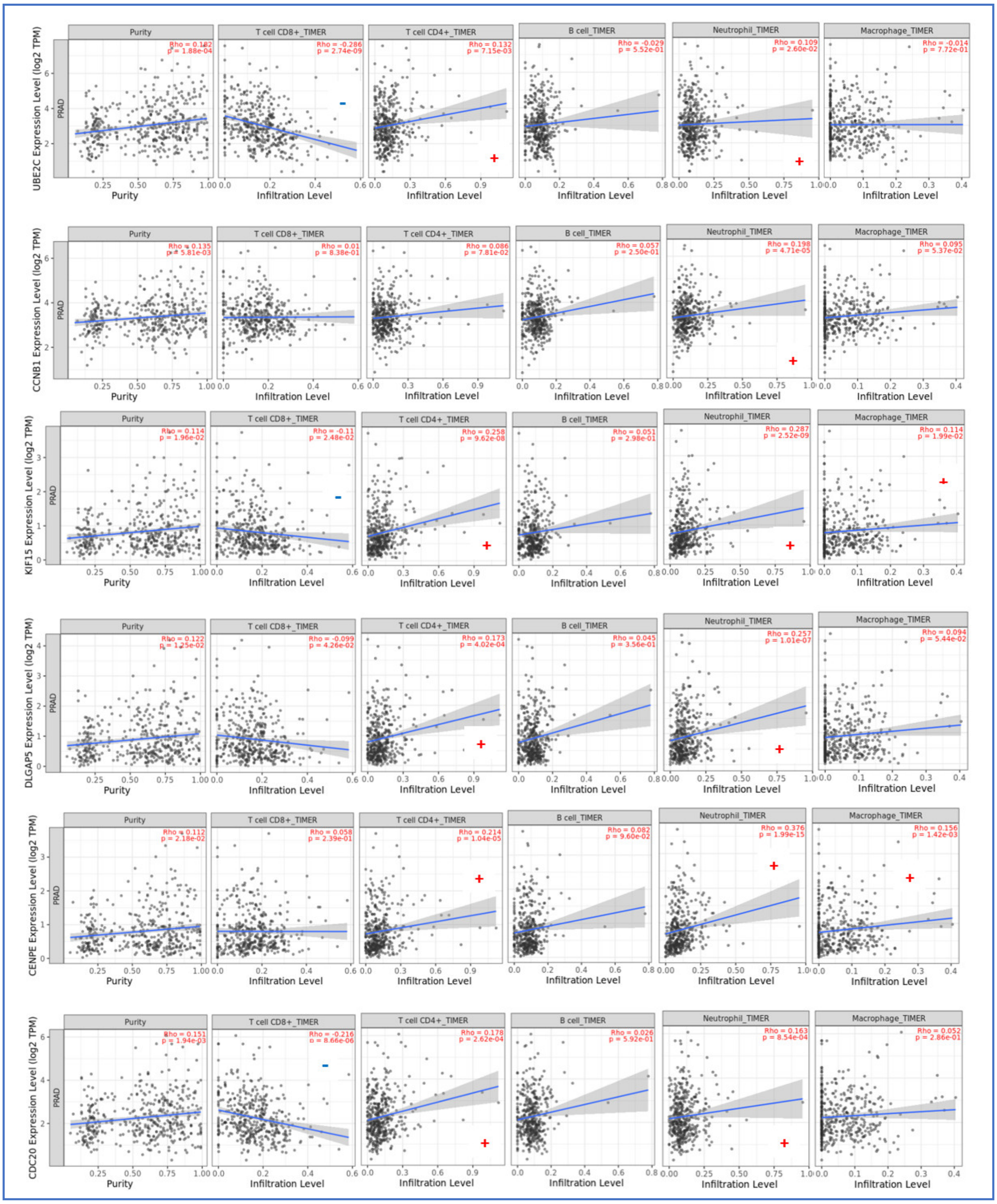

Figure S6: Association between differently expressed hub genes from RNA-seq datasets and immune cell infiltration for PCa from TIMER2.0 database. Immune cells include CD4 + T cells, CD8 + T cells, B cells, macrophages, and Neutrophils. Partial Spearman's Correlation and P-value (P < 0.05) determined the significance for (A) upregulated and (B) downregulated hub genes.

(A)

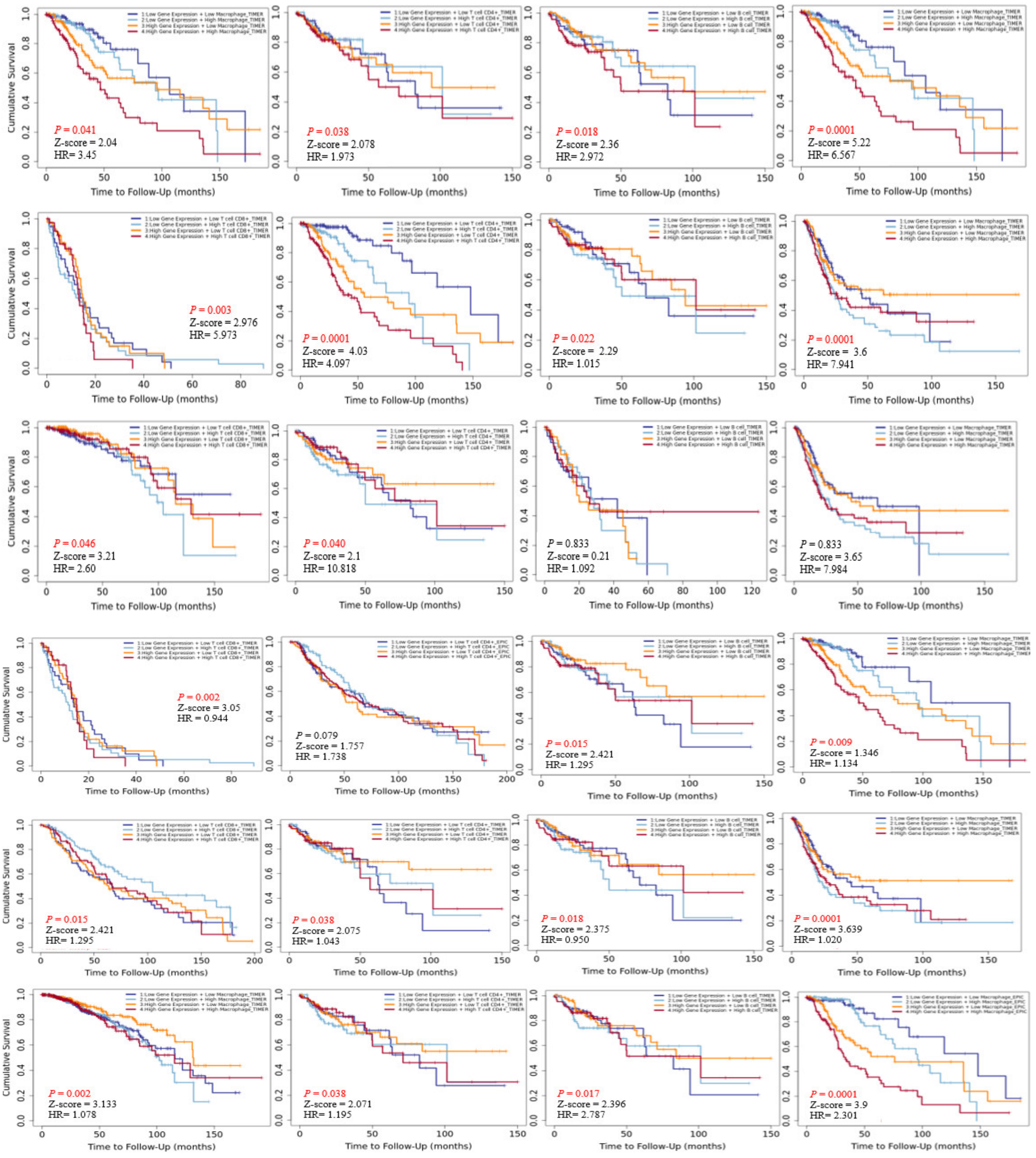
Cumulative Survival
Time to Follow-Up (months)
P = 0.041
Z-score = 2.04
HR= 3.45
P = 0.038
Z-score = 2.078
HR= 1.973
P = 0.018
Z-score = 2.36
HR= 2.972
P = 0.0001
Z-score = 5.22
HR= 6.567
P = 0.003
Z-score = 2.976
HR= 5.973
P = 0.0001
Z-score = 4.03
HR= 4.097
P = 0.022
Z-score = 2.29
HR= 1.015
P = 0.0001
Z-score = 3.6
HR= 7.941
P = 0.046
Z-score = 3.21
HR= 2.60
P = 0.040
Z-score = 2.1
HR= 10.818
P = 0.833
Z-score = 0.21
HR= 1.092
P = 0.833
Z-score = 3.65
HR= 7.984
P = 0.002
Z-score = 3.05
HR = 0.944
P = 0.079
Z-score = 1.757
HR = 1.738
P = 0.015
Z-score = 2.421
HR= 1.295
P = 0.009
Z-score = 1.346
HR= 1.134
P = 0.015
Z-score = 2.421
HR= 1.295
P = 0.038
Z-score = 2.075
HR= 1.043
P = 0.018
Z-score = 2.375
HR= 0.950
P = 0.0001
Z-score = 3.639
HR= 1.020
P = 0.002
Z-score = 3.133
HR= 1.078
P = 0.038
Z-score = 2.071
HR= 1.195
P = 0.017
Z-score = 2.396
HR= 2.787
P = 0.0001
Z-score = 3.9
HR= 2.301

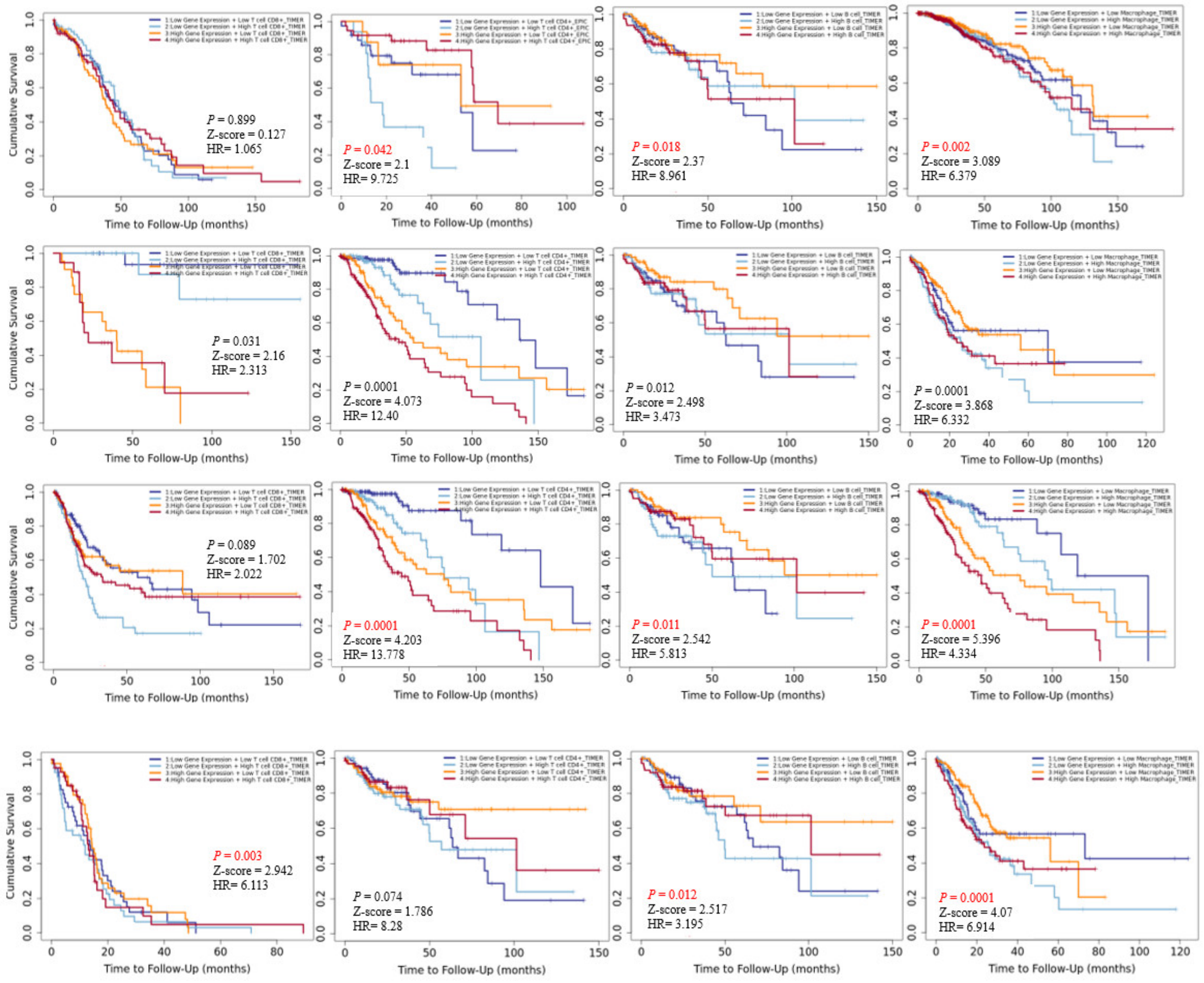
Cumulative Survival
Time to Follow-Up (months)
P = 0.899
Z-score = 0.127
HR= 1.065
P = 0.042
Z-score = 2.1
HR= 9.725
P = 0.018
Z-score = 2.37
HR= 8.961
P = 0.002
Z-score = 3.089
HR= 6.379
P = 0.031
Z-score = 2.16
HR= 2.313
P = 0.0001
Z-score = 4.073
HR= 12.40
P = 0.012
Z-score = 2.498
HR= 3.473
P = 0.0001
Z-score = 3.868
HR= 6.332
P = 0.089
Z-score = 1.702
HR= 2.022
P = 0.0001
Z-score = 4.203
HR= 13.778
P = 0.011
Z-score = 2.542
HR= 5.813
P = 0.0001
Z-score = 5.396
HR= 4.334
P = 0.003
Z-score = 2.942
HR= 6.113
P = 0.074
Z-score = 1.786
HR= 8.28
P = 0.012
Z-score = 2.517
HR= 3.195
P = 0.0001
Z-score = 4.07
HR= 6.914

(B)

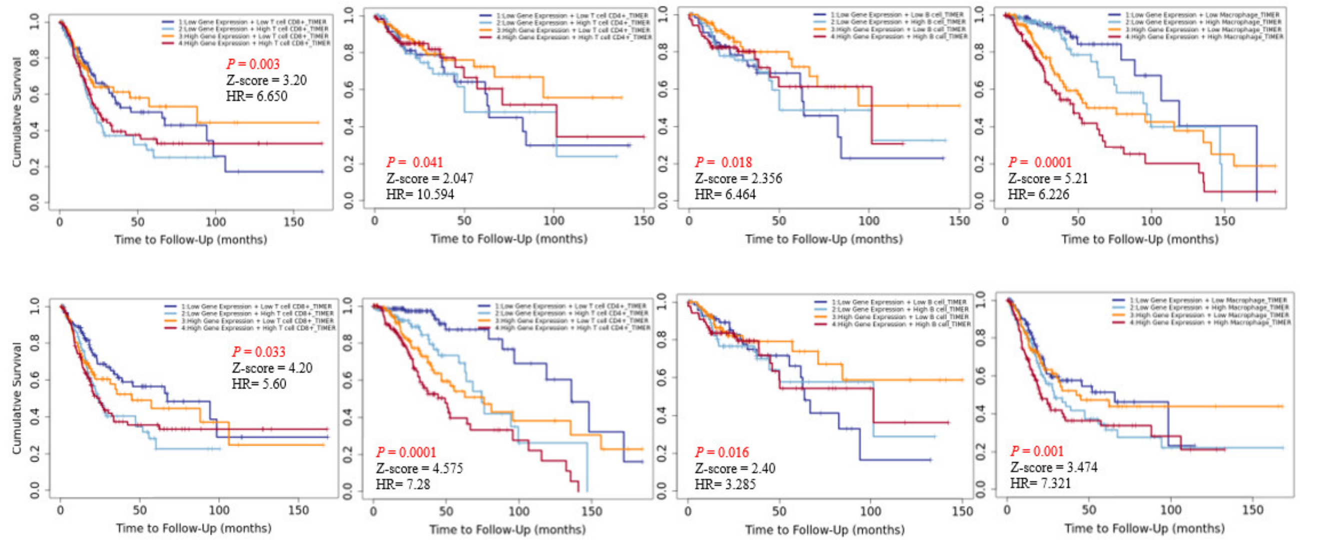
Cumulative Survival
Time to Follow-Up (months)
P = 0.003
Z-score = 3.20
HR= 6.650
P = 0.041
Z-score = 2.047
HR= 10.594
P = 0.018
Z-score = 2.356
HR= 6.464
P = 0.0001
Z-score = 5.21
HR= 6.226
P = 0.033
Z-score = 4.20
HR= 5.60
P = 0.0001
Z-score = 4.575
HR= 7.28
P = 0.016
Z-score = 2.40
HR= 3.285
P = 0.001
Z-score = 3.474
HR= 7.321

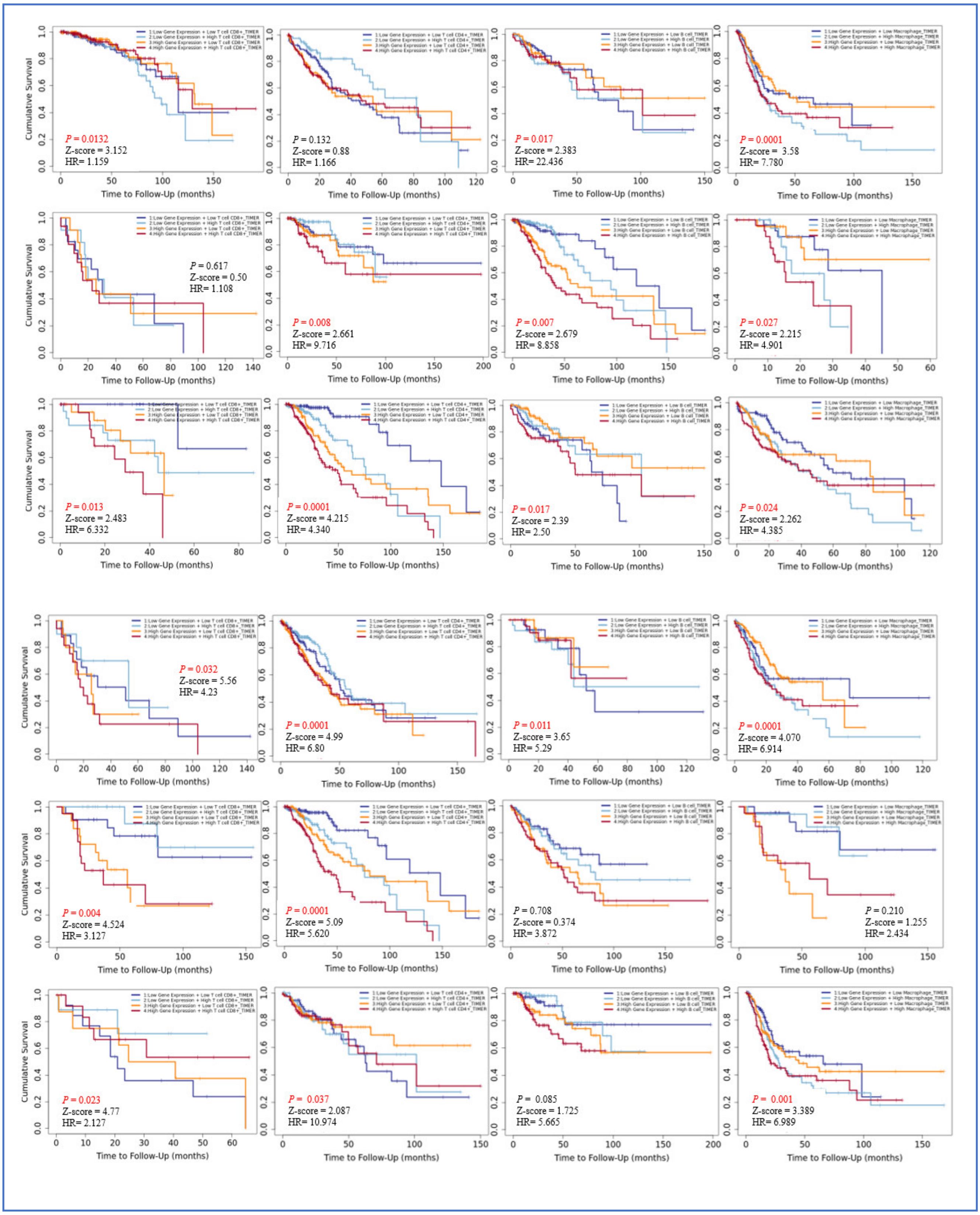

Cumulative Survival
Time to Follow-Up (months)
P = 0.0132
Z-score = 3.152
HR= 1.159
P = 0.132
Z-score = 0.88
HR= 1.166
P = 0.017
Z-score = 2.383
HR= 22.436
P = 0.0001
Z-score = 3.58
HR= 7.780
P = 0.617
Z-score = 0.50
HR= 1.108
P = 0.008
Z-score = 2.661
HR= 9.716
P = 0.007
Z-score = 2.679
HR= 8.858
P = 0.027
Z-score = 2.215
HR= 4.901
P = 0.013
Z-score = 2.483
HR= 6.332
P = 0.0001
Z-score = 4.215
HR= 4.340
P = 0.017
Z-score = 2.39
HR= 2.50
P = 0.024
Z-score = 2.262
HR= 4.385
P = 0.032
Z-score = 5.56
HR= 4.23
P = 0.0001
Z-score = 4.99
HR= 6.80
P = 0.011
Z-score = 3.65
HR= 5.29
P = 0.0001
Z-score = 4.070
HR= 6.914
P = 0.004
Z-score = 4.524
HR= 3.127
P = 0.0001
Z-score = 5.09
HR= 5.620
P = 0.708
Z-score = 0.374
HR= 3.872
P = 0.210
Z-score = 1.255
HR= 2.434
P = 0.023
Z-score = 4.77
HR= 2.127
P = 0.037
Z-score = 2.087
HR= 10.974
P = 0.085
Z-score = 1.725
HR= 5.665
P = 0.001
Z-score = 3.389
HR= 6.989

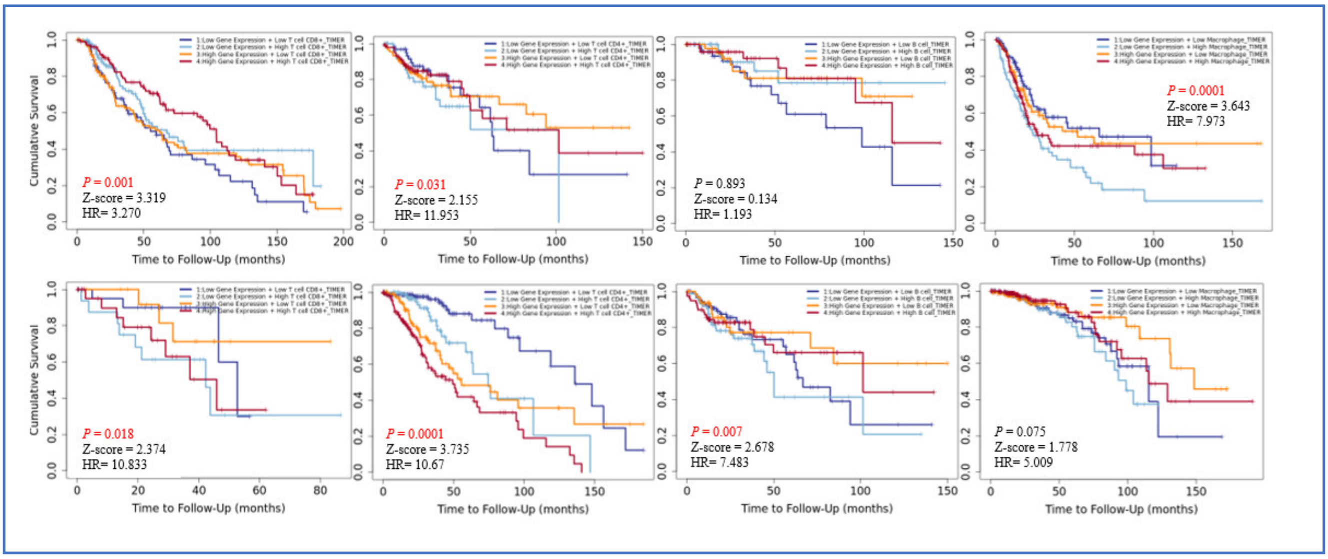

Figure S7: Kaplan-Meier Curves model Survivor analysis for hub genes from RNA-seq from PCa includes Immune Infiltrates, Gene Expression, and TIMMER (algorithm). Immune cells have CD4 + T cells, CD8 + T cells, B cells, and macrophages. Cox regression as the model selection-based P-value (P < 0.05), Hazard Ratio, and Z-score is considered as a statistically significant value for (A) upregulated and (B) downregulated genes. The dark blue line represents low gene expression and low immune cells. The light blue line represents low gene expression and high immune cells. The orange line represents high gene expression and low immune cells. The red line represents high gene expression and high immune cells.

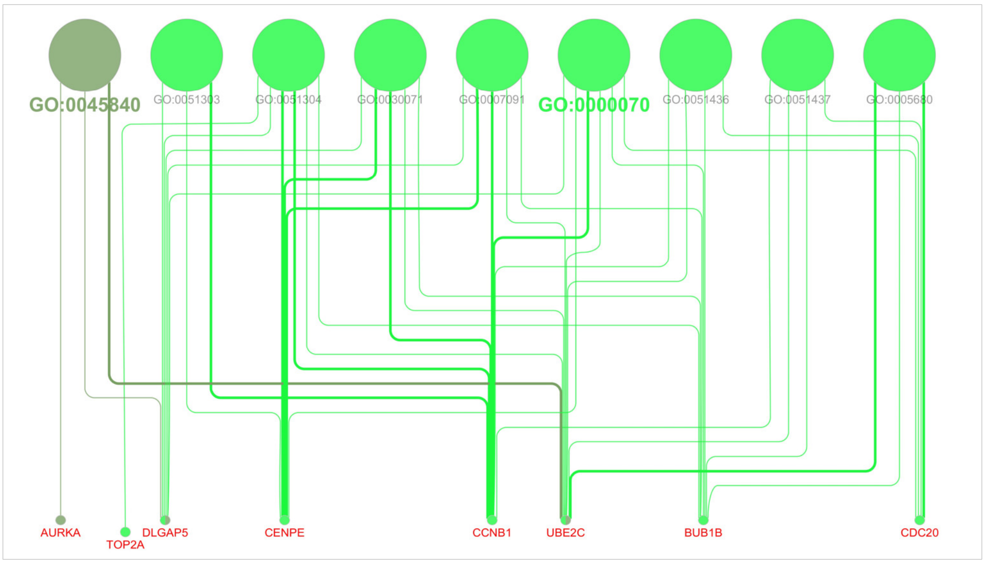
GO:0045840
GO:0051303
GO:0051304
GO:0030071
GO:0007091
GO:0000070
GO:0051436
GO:0051437
GO:0005680
AURKA
TOP2A
DLGAP5
CENPE
CCNB1
UBE2C
BUB1B
CDC20

(A1)

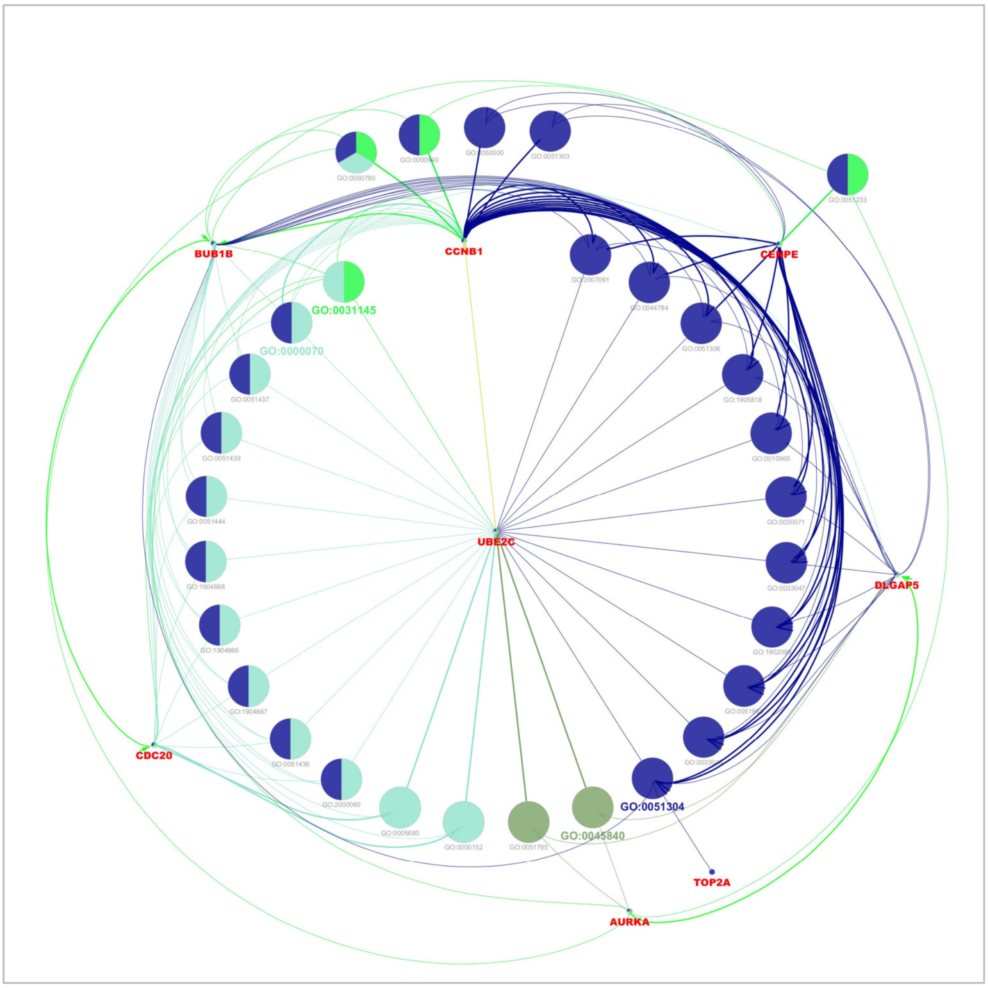
BUB1B
CCNB1
CENPE
GO:0031145
GO:0000070
UBE2C
DLGAP5
CDC20
GO:0051304
GO:0045840
TOP2A
AURKA

(A2)

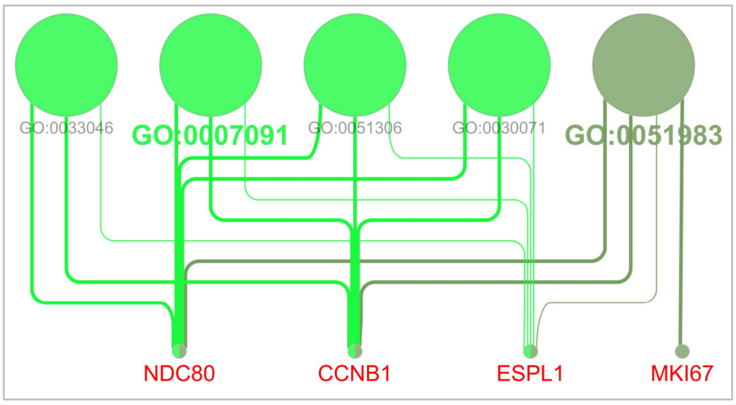

(B1)

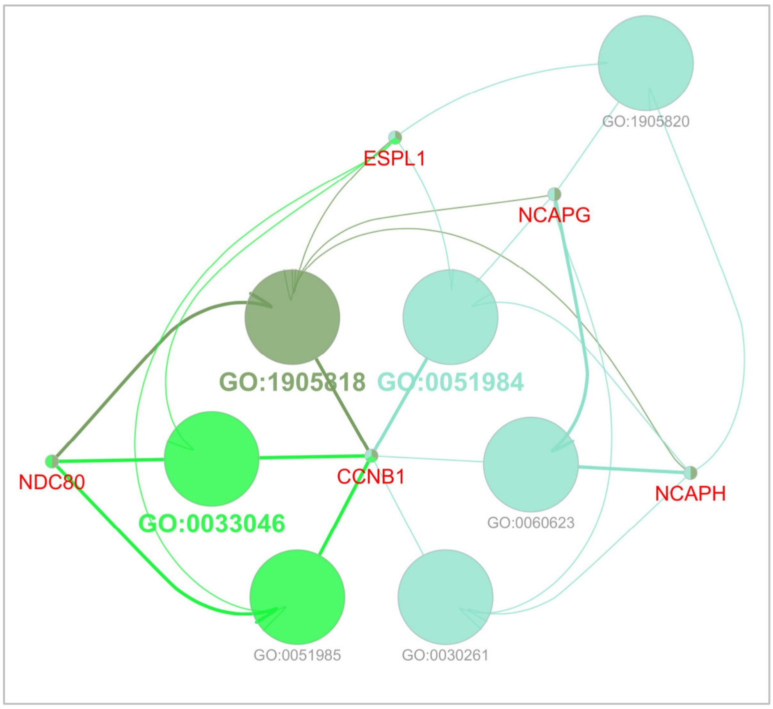

(B2)

Figure S8. The network connection was screened by employing the ClueGO and CluePedia on RNA-seq DEGs hub genes DEGs discovered from the obtained module. ClueGO: (A1) upregulated and (B1) downregulated are GO enrichment analysis network relationships. CluePedia: (A2) upregulated and (B2) downregulated network association of GO pathway enrichment analysis. Pathways with identical colors demonstrate that they have similar functions.

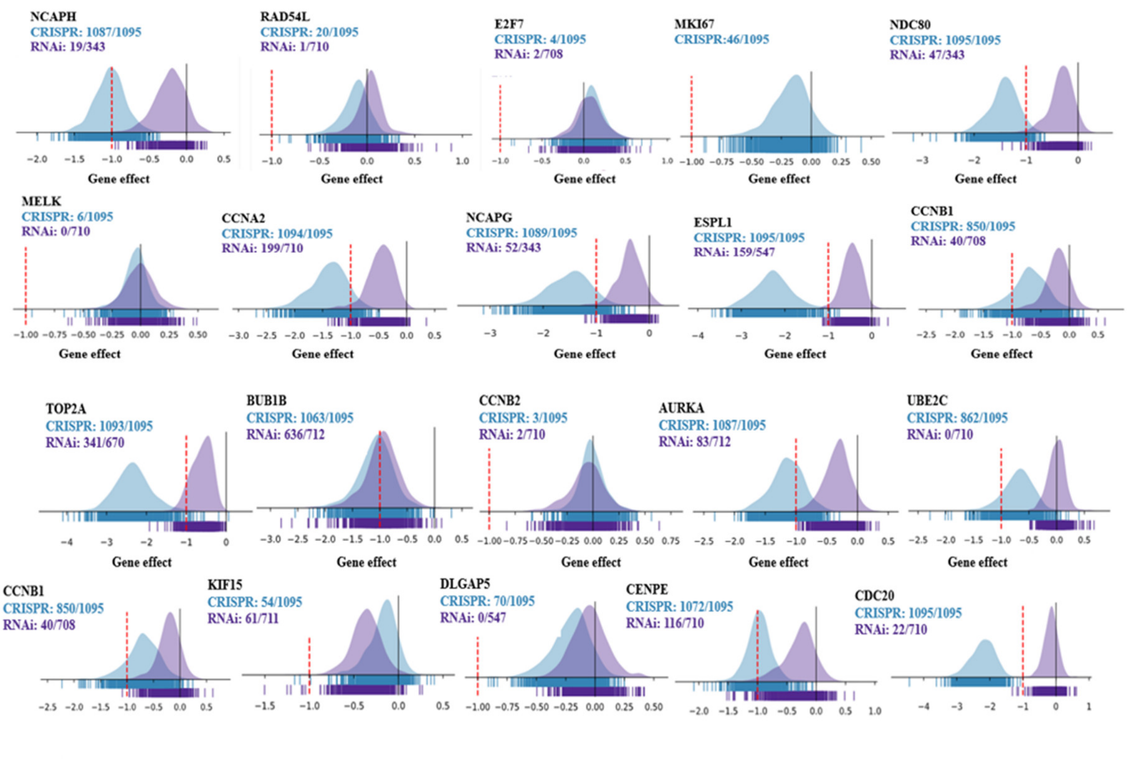
NCAPH
CRISPR: 1087/1095
RNAi: 19/343
RAD54L
CRISPR: 20/1095
RNAi: 1/710
E2F7
CRISPR: 4/1095
RNAi: 2/708
MKI67
CRISPR:46/1095
NDC80
CRISPR: 1095/1095
RNAi: 47/343
MELK
CRISPR: 6/1095
RNAi: 0/710
CCNA2
CRISPR: 1094/1095
RNAi: 199/710
NCAPG
CRISPR: 1089/1095
RNAi: 52/343
ESPL1
CRISPR: 1095/1095
RNAi: 159/547
CCNB1
CRISPR: 850/1095
RNAi: 40/708
TOP2A
CRISPR: 1093/1095
RNAi: 341/670
BUB1B
CRISPR: 1063/1095
RNAi: 636/712
CCNB2
CRISPR: 3/1095
RNAi: 2/710
AURKA
CRISPR: 1087/1095
RNAi: 83/712
UBE2C
CRISPR: 862/1095
RNAi: 0/710
CCNB1
CRISPR: 850/1095
RNAi: 40/708
KIF15
CRISPR: 54/1095
RNAi: 61/711
DLGAP5
CRISPR: 70/1095
RNAi: 0/547
CENPE
CRISPR: 1072/1095
RNAi: 116/710
CDC20
CRISPR: 1095/1095
RNAi: 22/710
Gene effect

(A)

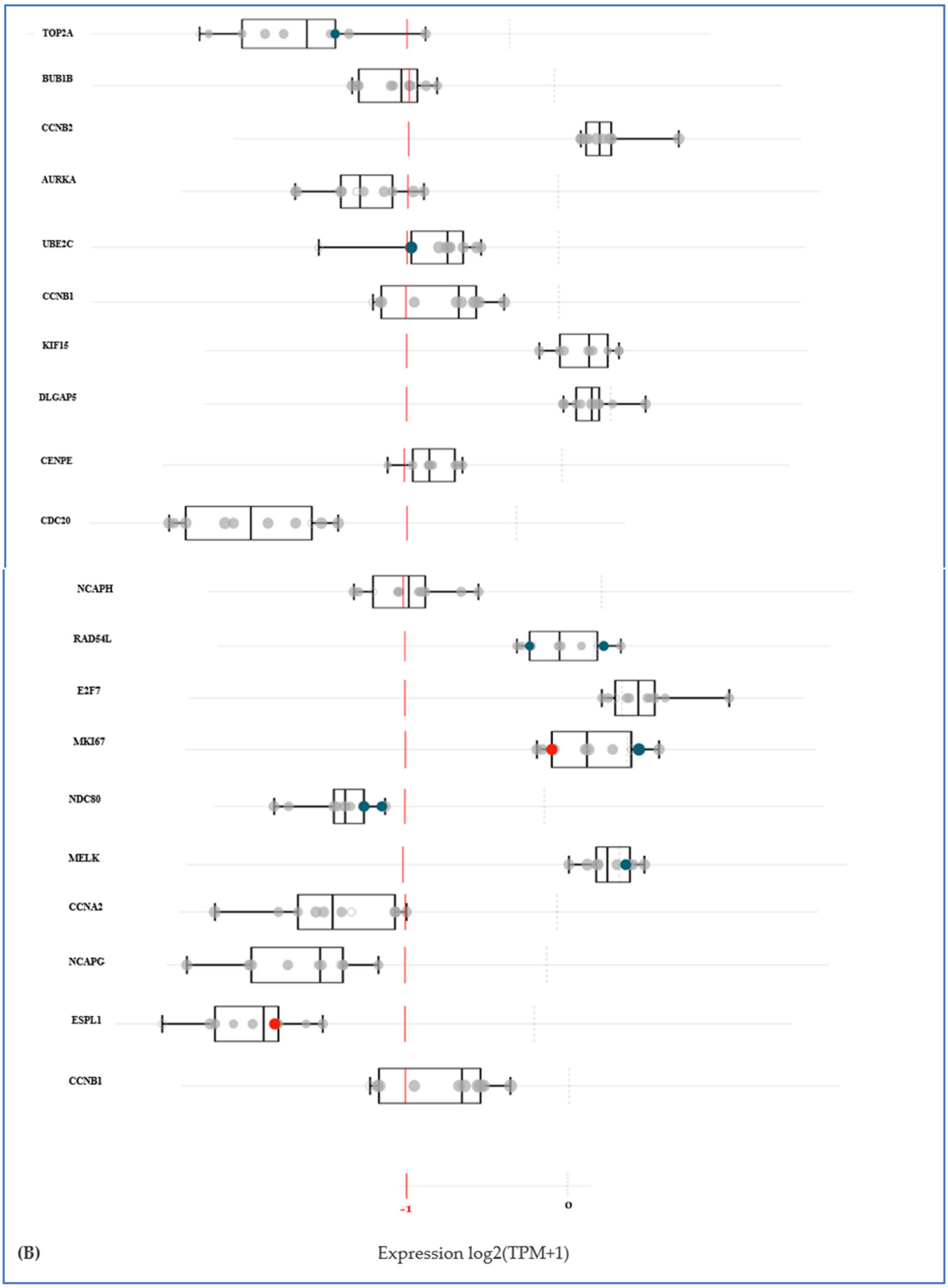

Figure S9: Analysis of Gene Redundancy in Hub Genes. This figure examines gene dependency and predictability in relation to baseline genomic and molecular characteristics of PCa cell lines, utilizing CRISPR/Cas9 and RNAi screening data. The Gene Effect Outcome, calculated from CERES scores, is presented as log2(TPM+1). Lower scores indicate higher gene dependency in a given cell line. A score of 0

suggests non-essential genes, while -1 equates to the median criticality of standard hub genes. Panel A analyzes the redundancy of twenty hub genes across all PCa lines tested in this study. Panel B displays the dependency scores (CERES/Expression log2(TPM+1)) of the twenty identified hub genes in PCa.

CRISPR (DepMap Public 23Q2+Score, Chronos) — NCAPH

MODEL: Core Omics — ACCURACY SCORE (Pearson): 0.382

Feature	Relative Importance	Correlation	Feature Type
SSMD	4.2%	0.08	Confounders
TUBB6	3.6%	0.23	Expression
NAT10	3.2%	0.29	Expression
NCAPH	3.2%	0.33	Expression
NCAPD2	3.1%	0.28	Expression
MED10	1.9%	0.04	Expression
SNW1	1.8%	0.27	Expression
VCP	1.6%	0.16	Expression
H2AC8	1.5%	-0.20	Expression
SUMO1	1.5%	0.22	Expression

CRISPR (DepMap Public 23Q2+Score, Chronos) — RAD54L

MODEL: Core Omics — ACCURACY SCORE (Pearson): 0.382

Feature	Relative Importance	Correlation	Feature Type
SSMD	6.4%	-0.01	Confounders
CCDC61	3.4%	-0.10	Expression
ATP6V1B2	2.5%	-0.06	Copy num.
TUBB3	2.2%	0.14	Expression
ALDH1A2	1.5%	-0.08	Copy num.
WDYHV1	1.4%	-0.08	Copy num.
LIMK1	1.4%	0.04	Expression
TUBB2A	1.3%	0.11	Expression
ATP6V1B2	1.3%	0.05	Expression
SPICE1	1.2%	-0.06	Expression

CRISPR (DepMap Public 23Q2+Score, Chronos) — E2F7

MODEL: Core Omics — ACCURACY SCORE (Pearson): 0.382

Feature	Relative Importance	Correlation	Feature Type
DCP1B	12.2%	0.20	Expression
SSMD	7.8%	0.09	Confounders
E2F8	5.9%	-0.06	Expression
E2F1	5.8%	-0.06	Expression
SMURF1	5.8%	0.09	Expression
CTBP1	5.2%	0.08	Expression
E2F1	5.0%	-0.06	Copy num.
DCP1B	4.8%	0.06	Copy num.
E2F7	4.7%	0.01	Copy num.
CTBP1	4.5%	0.12	Copy num.

CRISPR (DepMap Public 23Q2+Score, Chronos) — MKI67

MODEL: Core Omics — ACCURACY SCORE (Pearson): 0.208

Feature	Relative Importance	Correlation	Feature Type
REG4	1.9%	-0.17	Expression
FUT7	1.2%	-0.13	Expression
CYP2C18	0.8%	-0.17	Expression
MAML3	0.8%	-0.10	Expression
LSP1P5	0.8%	0.03	Copy num.
ASB9	0.7%	0.13	Expression
MSRB3	0.7%	0.14	Expression
DLX2	0.6%	0.12	Expression
UROD	0.6%	0.13	Expression
PPFIBP2	0.6%	-0.12	Expression

CRISPR (DepMap Public 23Q2+Score, Chronos) — NDC80

MODEL: Core Omics — ACCURACY SCORE (Pearson): 0.382

Feature	Relative Importance	Correlation	Feature Type
NDC80	5.9%	0.34	Copy num.
SMCHD1	4.4%	0.32	Copy num.
SMCHD1	3.9%	0.26	Expression
SSMD	2.8%	0.12	Confounders
NDC80	2.7%	0.31	Expression
AMOTL2	2.2%	0.14	Expression
GTF2H1	1.9%	0.20	Expression
SPDL1	1.6%	0.22	Expression
KIF18A	1.1%	0.09	Copy num.
NEK2	1.1%	-0.21	Copy num.

CRISPR (DepMap Public 23Q2+Score, Chronos) — MELK

MODEL: Core Omics — ACCURACY SCORE (Pearson): 0.382

Feature	Relative Importance	Correlation	Feature Type
SSMD	4.7%	-0.02	Confounders
ZNF622	4.2%	-0.15	Copy num.
EIF2A	3.7%	-0.04	Expression
ZNF622	3.2%	-0.01	Expression
PRKAA1	3.2%	-0.15	Copy num.
TRIB2	3.0%	0.04	Expression
SMAD7	3.0%	-0.07	Expression
MBP	2.9%	0.10	Expression
STK16	2.6%	-0.06	Expression
STK16	2.6%	-0.05	Copy num.

CRISPR (DepMap Public 23Q2+Score, Chronos) — ESPL1

MODEL: Core Omics — ACCURACY SCORE (Pearson): 0.382

Feature	Relative Importance	Correlation	Feature Type
KIRREL1	4.3%	0.25	Expression
CLASP1	3.4%	0.23	Expression
NDE1	3.1%	0.19	Expression
NDE1	3.0%	-0.07	Copy num.
SSMD	1.5%	0.10	Confounders
STAG1	1.2%	0.15	Copy num.
MAPRE1	1.1%	0.14	Copy num.
SGTB	1.1%	0.15	Expression
NXF1	1.0%	0.12	Expression
TRIM56	1.0%	0.18	Expression

CRISPR (DepMap Public 23Q2+Score, Chronos) — CCNA2

MODEL: Core Omics — ACCURACY SCORE (Pearson): 0.382

Feature	Relative Importance	Correlation	Feature Type
CDH1	4.3%	-0.24	Expression
SSMD	2.4%	-0.13	Confounders
CDC27	1.8%	0.01	Copy num.
RB1	1.7%	0.17	Expression
KAT2B	1.7%	0.11	Copy num.
USP6NL	1.4%	0.03	Copy num.
CKS1B	1.3%	-0.17	Copy num.
SKP2	1.3%	0.05	Expression
ELP3	1.3%	0.18	Expression
CCNB3	1.2%	0.04	Copy num.

CRISPR (DepMap Public 23Q2+Score, Chronos) — NCAPG

MODEL: Core Omics — ACCURACY SCORE (Pearson): 0.382

Feature	Relative Importance	Correlation	Feature Type
PTP4A3	3.6%	-0.12	Copy num.
EGFR	2.8%	0.20	Expression
TUBB6	2.6%	0.14	Expression
TUBB4B	2.3%	0.09	Expression
NCAPG	2.1%	0.13	Copy num.
TUBA3E	2.1%	-0.19	Expression
PTP4A3	1.7%	-0.14	Expression
HSPA8	1.6%	0.11	Copy num.
ONECUT1	1.6%	-0.16	Copy num.
SSMD	1.6%	-0.10	Confounders

CRISPR (DepMap Public 23Q2+Score, Chronos) — ESPL1

MODEL: Core Omics — ACCURACY SCORE (Pearson): 0.382

Feature	Relative Importance	Correlation	Feature Type
CDCA5	1.3%	-0.14	Copy num.
SPICE1	1.3%	-0.16	Expression
SSMD	1.2%	0.03	Confounders
ITGB3BP	1.2%	-0.09	Copy num.
RHOBTB3	1.1%	-0.13	Expression
AURKC	0.9%	-0.09	Expression
CENPM	0.9%	0.13	Expression
CDKN1A	0.9%	0.10	Expression
KIF22	0.8%	-0.03	Expression
RCC2	0.8%	-0.12	Copy num.

CRISPR (DepMap Public 23Q2+Score, Chronos) — TOP2A

MODEL: Core Omics — ACCURACY SCORE (Pearson): 0.287

Feature	Relative Importance	Correlation	Feature Type
RAG2	3.1%	0.24	Expression
ATP6V0A1	3.0%	-0.20	Expression
CYP4F8	2.9%	0.13	Expression
TOP2B	2.8%	0.21	Expression
DNTT	1.2%	0.25	Expression
MTCP1	1.0%	-0.15	Expression
KLRF2	0.7%	0.19	Expression
BAG3	0.7%	-0.22	Expression
MYB	0.7%	0.16	Expression
NDFIP2	0.6%	-0.15	Expression

CRISPR (DepMap Public 23Q2+Score, Chronos) — BUB1B

MODEL: Core Omics — ACCURACY SCORE (Pearson): 0.389

Feature	Relative Importance	Correlation	Feature Type
ScreenMADEssentials	4.7%	-0.22	Confounders
UBE2C	3.1%	-0.18	Expression
ScreenNNMD	1.9%	0.21	Confounders
WFDC2	1.4%	-0.22	Expression
CDA	1.2%	-0.26	Expression
ATP5F1A	1.0%	0.22	Expression
ScreenMedianNonessen...	1.0%	-0.18	Confounders
ARTN	1.0%	-0.20	Expression
CasActivity	0.9%	-0.22	Confounders
GJB3	0.8%	-0.24	Expression

CRISPR (DepMap Public 23Q2+Score, Chronos) — CCNB2

MODEL: Core Omics — ACCURACY SCORE (Pearson): 0.382

Feature	Relative Importance	Correlation	Feature Type
SSMD	4.5%	-0.07	Confounders
CDCA8	1.9%	0.15	Expression
NAP1L4	1.7%	0.06	Expression
SCUBE1	1.6%	0.08	Expression
EIF5A2	1.1%	-0.08	Copy num.
PRDX1	1.0%	-0.03	Expression
ATE1	0.9%	-0.03	Expression
CCND1	0.8%	-0.04	Expression
MYB	0.7%	-0.07	Expression
SCUBE1	0.7%	-0.02	Copy num.

CRISPR (DepMap Public 23Q2+Score, Chronos) — AURKA

MODEL: Core Omics — ACCURACY SCORE (Pearson): 0.382

Feature	Relative Importance	Correlation	Feature Type
SSMD	3.7%	-0.14	Confounders
DYNC1H1	3.0%	-0.17	Expression
SRSF1	1.8%	-0.11	Expression
TUBA1A	1.6%	-0.18	Expression
AURKA	1.0%	0.19	Copy num.
CNOT9	0.9%	-0.14	Expression
PASK	0.9%	-0.15	Copy num.
DKK3	0.7%	-0.14	Expression
HDAC2	0.7%	-0.16	Expression
USP3	0.6%	-0.05	Expression

CRISPR (DepMap Public 23Q2+Score, Chronos) — UBE2C

MODEL: Core Omics — ACCURACY SCORE (Pearson): 0.382

Feature	Relative Importance	Correlation	Feature Type
ScreenNNMD	8.3%	0.21	Confounders
BAG3	5.2%	-0.22	Expression
UBE2C	2.5%	-0.20	Copy num.
UBE2E1	2.4%	0.17	Expression
FZR1	1.7%	-0.07	Expression
ENO2	1.7%	-0.14	Expression
UBE2V1	1.2%	-0.15	Copy num.
ScreenMADEssentials	1.2%	-0.13	Confounders
ANAPC4	1.2%	0.03	Copy num.
ANAPC2	1.1%	0.05	Copy num.

CRISPR (DepMap Public 23Q2+Score, Chronos) — CCNB1

MODEL: Core Omics — ACCURACY SCORE (Pearson): 0.382

Feature	Relative Importance	Correlation	Feature Type
CCNB2	8.0%	0.21	Copy num.
CENPU	2.7%	0.19	Copy num.
MYB	2.3%	0.19	Expression
ZWILCH	2.0%	0.15	Copy num.
PAPSS1	1.5%	0.18	Copy num.
RELA	1.1%	-0.13	Expression
KLC3	1.0%	-0.18	Expression
NUP42	0.9%	0.10	Expression
ScreenFPR	0.8%	-0.20	Confounders
CCNB3	0.7%	-0.12	Expression

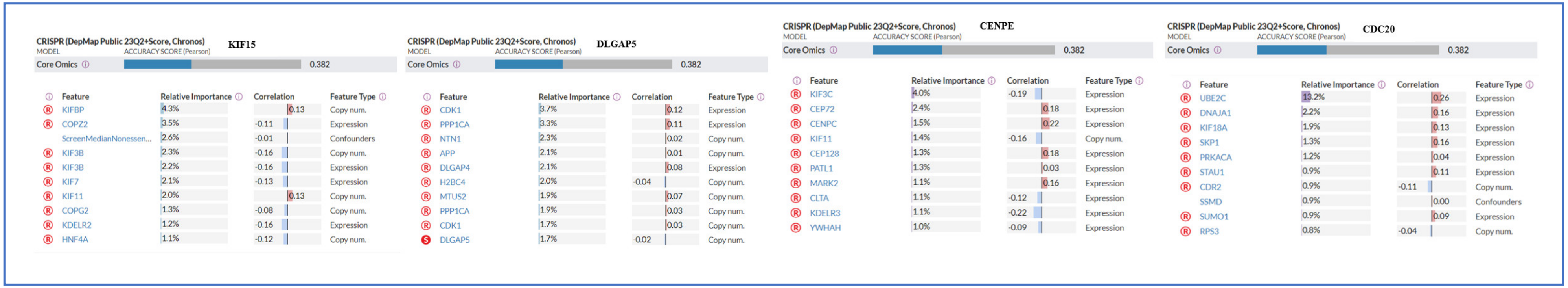

Figure S10. The predictability analysis for 20 hub genes for predicting the PCa cell lines' viability response to a given perturbation / predictive biomarkers. Core Omics include Expression, mutation (including damaging, hotspot, and other categories), fusions, copy numbers, tissue annotations, and screen confounders. Gene effect scores were calculated employing the Chronos algorithm for 20 hub genes and log2[foldchange]. Relative importance shows the effect of a unique feature on prediction accuracy comparable to the other elements known to the model from 0 to 100%. It is computed utilizing Gini Importance and is normalized so the total of all feature priority is 100%.

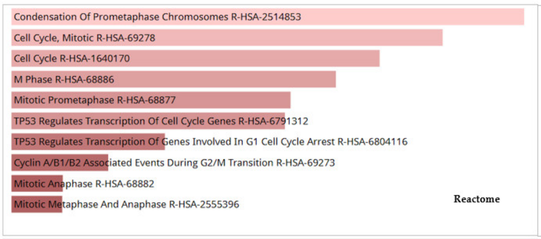

Term	P-value	OR
R-HSA-2514853	1.08^{-8}	1223.4
R-HSA-69278	5.97^{-8}	56.5
R-HSA-1640170	2.24^{-7}	44.8
R-HSA-68886	5.62^{-7}	52.3
R-HSA-68877	1.46^{-6}	72.6
R-HSA-6791312	1.64^{-6}	185.8
R-HSA-6804116	2.04^{-5}	416.2
R-HSA-69273	6.71^{-5}	217.0
R-HSA-68882	1.74^{-4}	36.9
R-HSA-2555396	1.76^{-4}	36.8

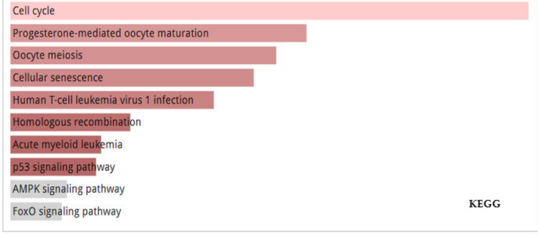

Term	P-value	OR
Cell cycle	2.70^{-5}	70.4
Progesterone-mediated oocyte maturation	1.085^{-3}	50.7
Oocyte meiosis	1.796^{-3}	39.1
Cellular senescence	2.611^{-3}	32.2
Human T-cell leukemia virus 1 infection	5.069^{-3}	22.8
Homologous recombination	0.020	55.4
Acute myeloid leukemia	0.033	33.5
p53 signaling pathway	0.035	30.7
AMPK signaling pathway	0.058	18.6
FoxO signaling pathway	0.063	16.9

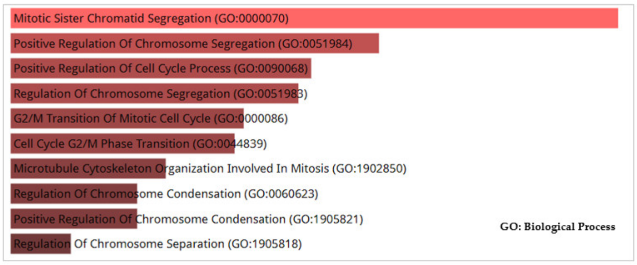

Term	P-value	OR
(GO:0000070)	1.18^{-9}	187.6
(GO:0051984)	7.32^{-8}	570.7
(GO:0090068)	2.35^{-7}	116.2
(GO:0051983)	$2.93E^{-7}$	342.3
(GO:0000086)	7.52^{-7}	244.3
(GO:0044839)	8.81^{-7}	231.1
(GO:1902850)	2.88^{-6}	152.6
(GO:0060623)	4.72^{-6}	999.3
(GO:1905821)	4.72^{-6}	999.3
(GO:1905818)	1.48^{-5}	499.5

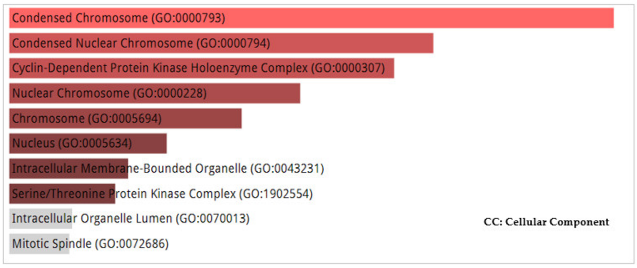

Term	P-value	OR
(GO:0000793)	3.03^{-6}	149.8
(GO:0000794)	8.45^{-5}	191.9
(GO:0000307)	1.74^{-4}	131.2
(GO:0000228)	9.80^{-4}	53.4
(GO:0005694)	0.0028	30.5
(GO:0005634)	0.011	5.2
(GO:0043231)	0.023	4.3
(GO:1902554)	0.029	37.5
(GO:0070013)	0.065	5.6
(GO:0072686)	0.069	15.5

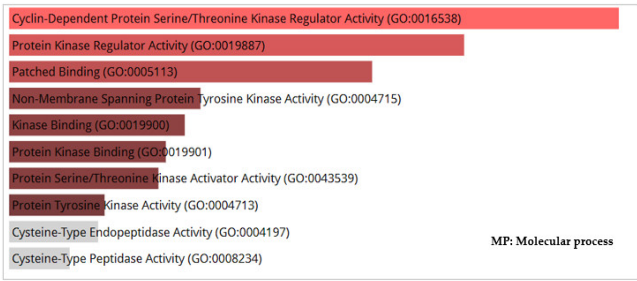

Term	P-value	OR
(GO:0016538)	2.30^{-4}	113.3
(GO:0019887)	1.151^{-3}	49.2
(GO:0005113)	2.997^{-3}	444.1
(GO:0004715)	0.017	63.3
(GO:0019900)	0.021	10.6
(GO:0019901)	0.025	9.56
(GO:0043539)	0.027	40.3
(GO:0004713)	0.048	22.5
(GO:0004197)	0.051	21.0
(GO:0008234)	0.069	15.4

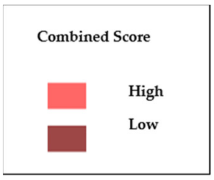

(A)

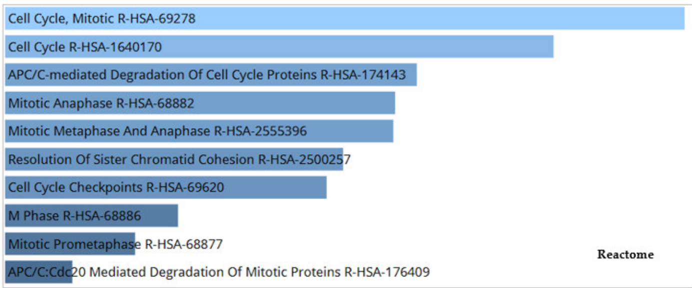

Term	P-value	OR
R-HSA-69278	8.91E-12	151.2
R-HSA-1640170	5.32E-11	119.7
R-HSA-174143	3.43E-10	242.7
R-HSA-68882	4.61E-10	131.2
R-HSA-2555396	4.73E-10	130.6
R-HSA-2500257	9.38E-10	196.9
R-HSA-69620	1.18E-09	111.7
R-HSA-68886	8.91E-09	78.7
R-HSA-68877	1.60E-08	109.4
R-HSA-176409	3.76E-08	187.1

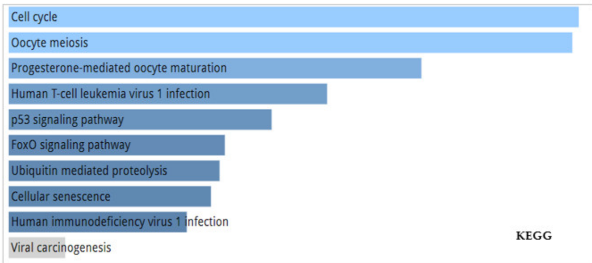

Term	P-value	OR
Cell cycle	2.87^{-7}	110.4
Oocyte meiosis	3.37^{-7}	105.9
Progesterone-mediated oocyte maturation	1.42^{-4}	87.9
Human T-cell leukemia virus 1 infection	1.47^{-4}	39.2
p53 signaling pathway	5.80^{-4}	70.1
FoxO signaling pathway	1.85^{-3}	38.5
Ubiquitin mediated proteolysis	2.11^{-3}	35.9
Cellular senescence	2.61^{-3}	32.2
Human immunodeficiency virus 1 infection	4.75^{-3}	23.5
Viral carcinogenesis	0.097	10.9

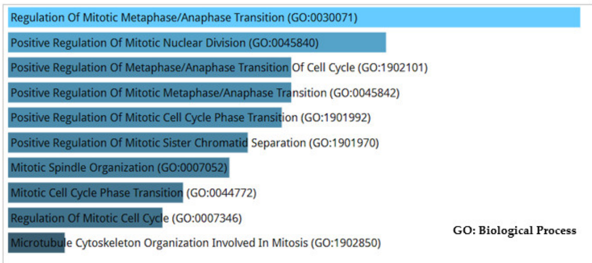

Term	P-value	OR
(GO:0030071)	1.79^{-11}	453.3
(GO:0045840)	1.64^{-9}	429.2
(GO:1902101)	1.48^{-8}	1070.5
(GO:0045842)	1.48^{-8}	1070.5
(GO:1901992)	1.85^{-8}	225.2
(GO:1901970)	4.08^{-8}	713.5
(GO:0007052)	6.26^{-8}	163.9
(GO:0044772)	1.84^{-7}	123.9
(GO:0007346)	2.97^{-7}	109.5
(GO:1902850)	2.88^{-6}	152.6

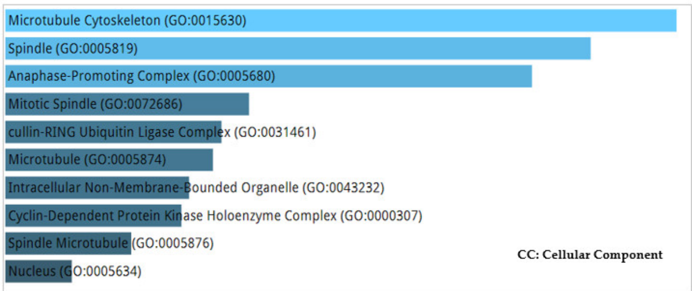

Term	P-value	OR
(GO:0015630)	4.74^{-9}	87.7
(GO:0005819)	2.94^{-8}	96.5
(GO:0005680)	1.02^{-7}	503.5
(GO:0072686)	4.14^{-5}	60.8
(GO:0031461)	7.43^{-5}	49.7
(GO:0005874)	8.91^{-5}	46.6
(GO:0043232)	1.48^{-4}	15.8
(GO:0000307)	1.74^{-4}	131.3
(GO:0005876)	5.04^{-4}	75.5
(GO:0005634)	1.78^{-4}	8.1

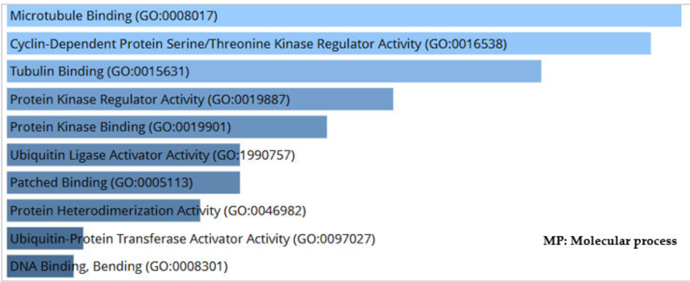

Term	P-value	OR
(GO:0008017)	1.90^{-4}	35.87
(GO:0016538)	2.30^{-4}	113.33
(GO:0015631)	4.56^{-4}	26.43
(GO:0019887)	1.15^{-3}	49.23
(GO:0019901)	1.74^{-3}	16.44
(GO:1990757)	2.99^{-3}	444.11
(GO:0005113)	2.99^{-3}	444.11
(GO:0046982)	3.84^{-3}	26.33
(GO:0097027)	7.90^{-3}	147.96
(GO:0008301)	8.45^{-3}	138.71

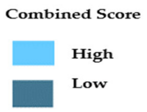

(B)

Figure S11. GO and pathways enrichment analysis terms of (A) upregulated group of genes and (B) downregulated group of hub genes from RNA-seq datasets associated with PCa cell lines.

Table S3. TFs that regulate genes of upregulated group are indicated by utilizing the TRRUST database.

Key TF	Description	P-value	Q-value
E2F4	E2F transcription factor 4, p107/p130-binding	8.82^{-11}	9.70^{-10}
MYC	MYC Proto-Oncogene, BHLH Transcription Factor.	6.06^{-8}	3.33^{-7}
RBL1	retinoblastoma-like 1 (p107)	1.68^{-5}	6.16^{-5}
E2F3	E2F transcription factor 3	1.3^{-4}	3.58^{-4}
ARNT	aryl hydrocarbon receptor nuclear translocator	3.16^{-4}	5.79^{-4}
YBX1	Y box binding protein 1	7.17^{-4}	1.13^{-3}
IRF1	interferon regulatory factor 1	2.06^{-3}	2.84^{-3}
ESR1	estrogen receptor 1	4.52^{-3}	5.53^{-3}
E2F1	E2F transcription factor 1	0.014	0.015
SP1	Sp1 transcription factor	0.029	0.022
TP53	tumor protein p53	0.033	0.042

Table S4. TFs that regulate genes of downregulated groups are indicated by utilizing the TRRUST database.

Key TF	Description	P-value	Q value
YBX1	Y box binding protein 1	4.32^{-7}	2.59^{-6}
MYC	MYC Proto-Oncogene, BHLH Transcription Factor	1.22^{-6}	1.46^{-3}
E2F4	E2F transcription factor 4	3.10^{-5}	0.037
MED1	mediator complex subunit 1	2.53^{-4}	7.58^{-6}
E2F3	E2F transcription factor 3	1.97^{-5}	3.93^{-5}
E2F1	E2F transcription factor 1	4.05^{-4}	6.08^{-5}
TP53	tumor protein p53	3.23^{-3}	3.23^{-3}
OTX2	orthodenticle homeobox 2	0.016	0.025
PTTG1	pituitary tumor-transforming 1	0.025	0.034
IRF1	interferon regulatory factor 1	0.039	0.042

Table S5. Disease Ontology Identifiers (DOIDs) and pathways associated with E2F4, MYC, and YBX1.

TFs	DO term	Term ID*	P-value	FDR
	Malignant neoplasm of prostate	DOID:10283	6.53^{-11}	2.03^{-12}
	Prostate carcinoma	DOID:10286	5.20^{-11}	1.13^{-12}
E2F4	Embryoma	DOID:4766	3.41^{-10}	2.16^{-11}
	Breast Carcinoma	DOID:3459	1.81^{-9}	1.37^{-10}
	Renal Cell Carcinoma	DOID:4450	9.17^{-9}	8.29^{-10}
	Malignant Neoplasms	DOID:462	6.12^{-27}	3.94^{-29}
	Malignant neoplasm of prostate	DOID:10283	5.05^{-26}	2.63^{-26}
MYC	Prostate carcinoma	DOID:10286	6.82^{-25}	4.41^{-25}
	cancer	DOID:162	2.72^{-22}	2.88^{-24}
	Embryoma	DOID:4766	5.61^{-20}	7.55^{-22}
	Malignant neoplasm of prostate	DOID:10283	2.05^{-12}	1.10^{-13}
	Malignant neoplasm of breast	DOID:4241	3.84^{-12}	1.55^{-13}
YBX1	cancer	DOID:162	5.94^{-12}	2.49^{-13}
	Carcinoma	DOID:305	3.92^{-11}	2.00^{-12}
	Malignant Neoplasms	DOID:462	7.47^{-11}	4.02^{-11}

Table S6. KEGG pathway and disease terms pathways associated with E2F4, MYC, and YBX1.

TFs	KEGG term	Term ID	P-value	FDR
	Cell cycle	hsa04110	2.20^{-9}	1.23^{-10}
	Pathways in cancer	hsa05200	9.05^{-9}	5.95^{-10}
E2F4	FoxO signaling pathway	hsa04068	3.95^{-6}	5.71^{-8}
	Prostate cancer	hsa05215	2.14^{-6}	4.22^{-7}
	PI3K-Akt signaling pathway	hsa04151	5.68^{-6}	8.71^{-7}
	p53 signaling pathway	hsa04115	1.92^{-5}	3.69^{-6}
	p53 signaling pathway	hsa04115	5.03^{-17}	8.23^{-19}
	Prostate cancer	hsa05215	$4.72E^{-16}$	1.83^{-18}
MYC	PI3K-Akt signaling pathway	hsa04151	1.07^{-11}	4.47^{-17}
	Pathways in cancer	hsa05203	3.94^{-10}	4.14^{-16}
	MicroRNAs in cancer	hsa05206	3.07^{-09}	3.56^{-14}
	Prostate cancer	hsa05215	4.60^{-11}	1.03^{-13}
	Pathways in cancer	hsa05200	1.90^{-10}	8.37^{-12}
YBX1	Adherens junction	hsa04520	5.78^{-08}	$4.^{79-09}$
	Proteoglycans in cancer	hsa05205	3.91^{-06}	5.65^{-07}
	Cell cycle	hsa04110	7.48^{-06}	1.21^{-06}

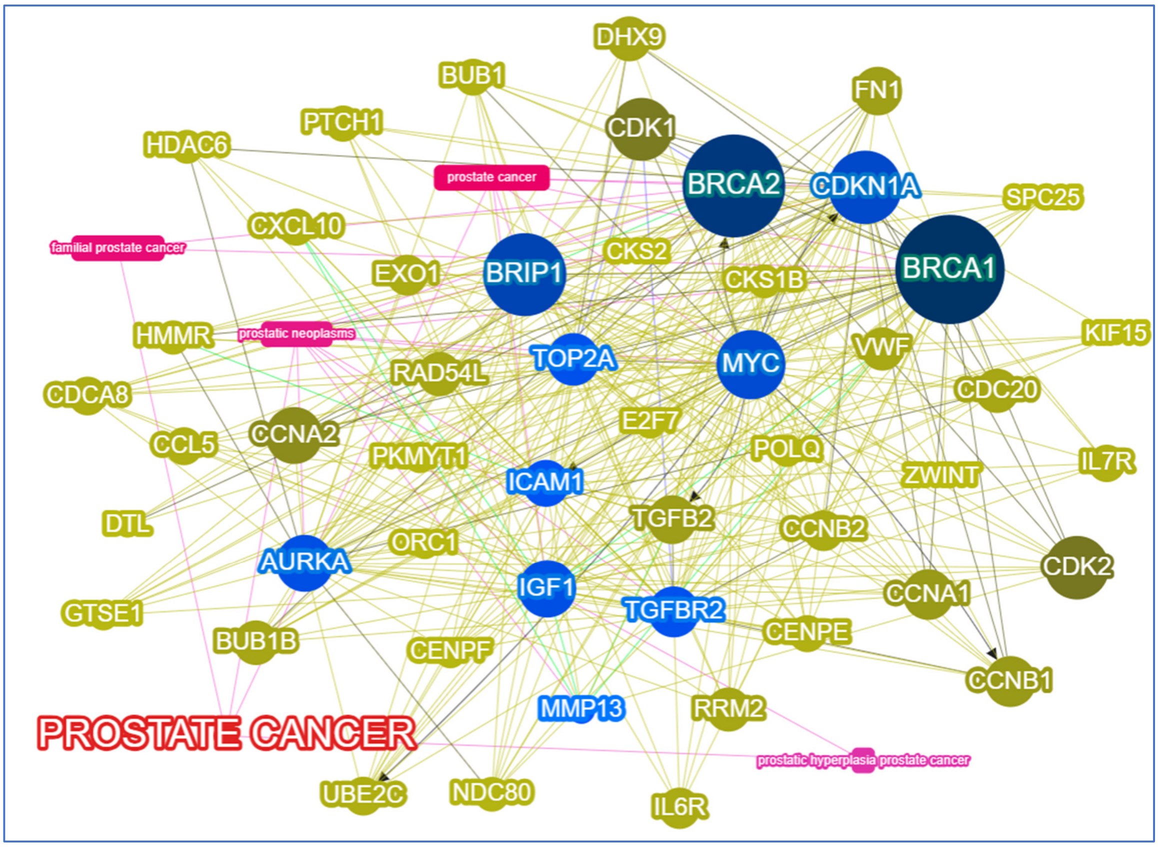

Figure S12: Phenolyzer analysis to associate hub genes with clinical terms related to prostate cancer, including 'prostate cancer,' 'prostate neoplasia,' 'prostatic neoplasia,' and 'prostate hyperplasia.' The color coding ranges from dark blue (indicating the highest significance) to yellow (indicating the most negligible significance), with each term's size corresponding to its significance level.

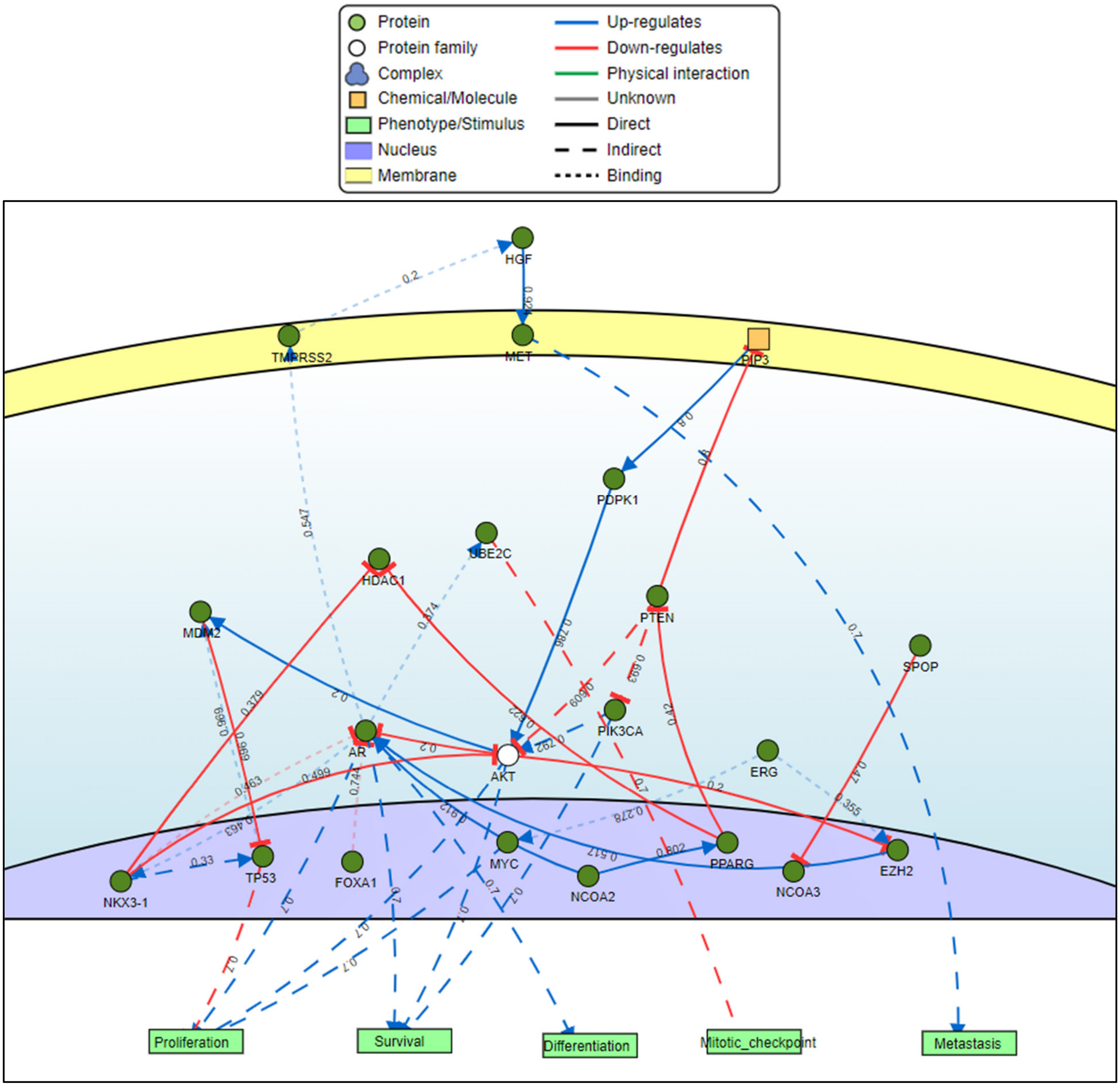

Figure S13: Functional Analysis and Causal Interactions of Genes/Proteins. This figure presents a causal interaction analysis of hub genes using SIGNOR 3.0, integrating resources like DisNor, Myo-REG, and CancerGeneNet. The analysis includes 27 seed entities encompassing proteins/genes, protein families, molecules, and phenotypic stimulations. It exhibits connections among upregulated & downregulated groups of genes and physical interactions occurring within the membrane or nucleus.
